# The effector AvrRxo1 phosphorylates NAD *in planta*

**DOI:** 10.1371/journal.ppat.1006442

**Published:** 2017-06-19

**Authors:** Teja Shidore, Corey D. Broeckling, Jay S. Kirkwood, John J. Long, Jiamin Miao, Bingyu Zhao, Jan E. Leach, Lindsay R. Triplett

**Affiliations:** 1 Department of Plant Pathology and Ecology, The Connecticut Agricultural Experiment Station, New Haven, CT, United States of America; 2 Proteomics and Metabolomics Facility, Colorado State University, Fort Collins, CO, United States of America; 3 Department of Bioagricultural Sciences and Pest Management, Colorado State University, Fort Collins, CO, United States of America; 4 Department of Horticulture, Virginia Polytechnic Institute and State University, Blacksburg, VA, United States of America; University of California Riverside, UNITED STATES

## Abstract

Gram-negative bacterial pathogens of plants and animals employ type III secreted effectors to suppress innate immunity. Most characterized effectors work through modification of host proteins or transcriptional regulators, although a few are known to modify small molecule targets. The *Xanthomonas* type III secreted avirulence factor AvrRxo1 is a structural homolog of the zeta toxin family of sugar-nucleotide kinases that suppresses bacterial growth. AvrRxo1 was recently reported to phosphorylate the central metabolite and signaling molecule NAD *in vitro*, suggesting that the effector might enhance bacterial virulence on plants through manipulation of primary metabolic pathways. In this study, we determine that AvrRxo1 phosphorylates NAD *in planta*, and that its kinase catalytic sites are necessary for its toxic and resistance-triggering phenotypes. A global metabolomics approach was used to independently identify 3’-NADP as the sole detectable product of AvrRxo1 expression in yeast and bacteria, and NAD kinase activity was confirmed *in vitro*. 3’-NADP accumulated upon transient expression of AvrRxo1 in *Nicotiana benthamiana* and in rice leaves infected with *avrRxo1*-expressing strains of *X*. *oryzae*. Mutation of the catalytic aspartic acid residue D193 abolished AvrRxo1 kinase activity and several phenotypes of AvrRxo1, including toxicity in yeast, bacteria, and plants, suppression of the flg22-triggered ROS burst, and ability to trigger an R gene-mediated hypersensitive response. A mutation in the Walker A ATP-binding motif abolished the toxicity of AvrRxo1, but did not abolish the 3’-NADP production, virulence enhancement, ROS suppression, or HR-triggering phenotypes of AvrRxo1. These results demonstrate that a type III effector targets the central metabolite and redox carrier NAD *in planta*, and that this catalytic activity is required for toxicity and suppression of the ROS burst.

## Author summary

Infectious bacteria have many strategies to weaken the defenses of their hosts. One common strategy is to inject proteins called effectors into the host cells. Many effectors inactivate proteins that transmit defense signals, disabling the alarm systems that alert the plant to defend against the pathogen. In this paper, we show that pathogens can also target very important small molecules in eukaryotes. We found that the plant pathogen effector AvrRxo1 adds a modification to NAD, a molecule required for hundreds of respiratory and signaling reactions, when expressed inside yeast or plant cells. This modification produces an unusual molecule, 3’-NADP, that is not able to be used efficiently by the cell. This finding demonstrates a new way that effectors can target essential central metabolites to manipulate the host.

## Introduction

A central theme in gram-negative bacterial pathogenesis is the injection of Type III-secreted effectors (T3E) into host cells. Plant pathogen effectors may suppress two tiers of innate immunity: PAMP-Triggered Immunity (PTI), triggered by perception of pathogen-associated molecular patterns (PAMPs) by pattern recognition receptors, or Effector-Triggered Immunity (ETI), triggered by perception of specific T3E by cognate nucleotide binding-leucine rich repeat resistance (R) genes [1]. With the exception of the DNA-binding Transcriptional Activator-Like effectors of *Xanthomonas*, the known molecular functions of T3E were once thought to be limited to the mimicry or covalent modification of host proteins involved in signaling [2]. Recently, the *Ralstonia solanacearum* effectors RipTPS and RipAY were reported to synthesize the sugar trehalose-6-phosphate and degrade the small peptide glutathione, respectively, demonstrating that small molecules might also be strategic targets of T3E in plants [3, 4].

AvrRxo1 (also called XopAJ) is a T3E produced by several species of *Xanthomonas*, *Acidovorax*, and *Burkholderia* plant pathogens of monocot and dicot plants. Putative homologs lacking the T3 secretion signal are also found in a variety of environmental bacteria with no known pathogenic role [5]. AvrRxo1 has been implicated in several different T3E functions; it triggers a type III secretion-dependent hypersensitive resistance response (HR) in maize or transgenic rice plants expressing the resistance protein Rxo1 [6, 7], enhances virulence of *Xanthomonas oryzae* on rice [8], and suppresses nonhost resistance to *X*. *oryzae* on tobacco [9]. Homologs of AvrRxo1 are always encoded upstream of a gene encoding a small protein binding partner, Arc1.

The solved structure of the *X*. *oryzae* homolog of AvrRxo1 revealed a T4 polynucleotide kinase (T4pnk) domain [8], typically involved in phosphorylation of polynucleotides and nucleoside small molecules [10]. Among T4pnk domain proteins, AvrRxo1 shares the strongest structural homology with a zeta (ζ) toxin, part of a toxin-antitoxin (TA) system from *Streptococcus pyogenes* [8, 11]. TA systems are ubiquitous gene modules comprised of a bacterial growth-suppressing toxin and a neutralizing antitoxin, which function in metabolic stress management and DNA maintenance [12]. Consistent with this structural homology, AvrRxo1 is bacteriostatic to *Escherichia coli* in the absence of Arc1, demonstrating that AvrRxo1 functions as TA system toxin in addition to a T3E [5]. AvrRxo1 also triggers growth suppression and watersoaked tissue collapse when expressed in yeast and plant cells, respectively [8, 13]. The bacteriostatic function of AvrRxo1 is dependent on two key functional residues conserved among T4pnk domain proteins. A catalytic aspartic acid (D193) is predicted to coordinate the hydroxyl group on the target carbon of the substrate, activating it for nucleophilic attack, and a Walker A threonine (T167) is predicted to coordinate the ATP [8, 14].

AvrRxo1’s toxic effects in prokaryotes and eukaryotes suggests that it has a universally essential target. While many TA toxins work by disrupting aspects of polynucleotide replication or translation, zeta toxin phosphorylates a product of central metabolism, the peptidoglycan precursor uridine diphosphate-N acetylglucosamine (UNAG), to generate a nonfunctional and inhibitory analog [15]. Despite its structural similarity to zeta toxin, AvrRxo1 differs at most predicted substrate-coordinating residues and does not phosphorylate UNAG [8]. Recently, Schuebel and colleagues used a rational bottom-up approach to demonstrate that AvrRxo1 acts as a nucleotide kinase that phosphorylates NAD and its derivative NAAD at the 3’ hydroxyl position *in vitro*, and also generates the product 3’-NADP in targeted assays in *E*. *coli* [16]. However, the presence and broader implications of this kinase activity in eukaryotic and plant host systems were not investigated, and the relevance of NAD phosphorylation to toxicity or immune suppression remains unclear.

Here, we used a top-down global metabolomics approach to identify molecular targets of AvrRxo1. Accumulation of 3’-NADP was the most substantial detected metabolic consequence of AvrRxo1 expression in yeast and bacteria. Targeted assays revealed 3’-NADP accumulation in plant tissue upon transient expression of *avrRxo1* in leaves of *N*. *benthamiana*, and during infection of rice with an *avrRxo1*-containing strain of *X*. *oryzae*. The catalytic aspartic acid D193 was essential for NAD phosphorylation and for the AvrRxo1 phenotypes of toxicity, Rxo1-triggered immunity, and ROS suppression. A mutation in the Walker A threonine strongly reduced 3’-NADP accumulation and abolished AvrRxo1 toxic phenotypes, but did not eliminate the ability of AvrRxo1 to trigger Rxo1 or suppress the flg22-mediated ROS burst in plants. These results demonstrate that T3E can directly modify a central primary metabolite in eukaryotic cells, representing a novel strategy for immune triggering and suppression by secreted effectors.

## Results

### Involvement of predicted key catalytic sites in toxic and resistance gene-triggering effects of AvrRxo1

Structural analysis of AvrRxo1 identified several conserved residues predicted to be required for catalytic activity. Mutation of two of these, the catalytic aspartate D193 and the Walker A threonine T167, abolished growth-suppressive activity of AvrRxo1 in *E*. *coli* [8]. Here, we asked whether the predicted sites D193 and T167 are necessary for other reported phenotypes of AvrRxo1, including yeast growth suppression, watersoaking upon transient expression in plants, and triggering the Rxo1-dependent HR.

**Fig 1:**
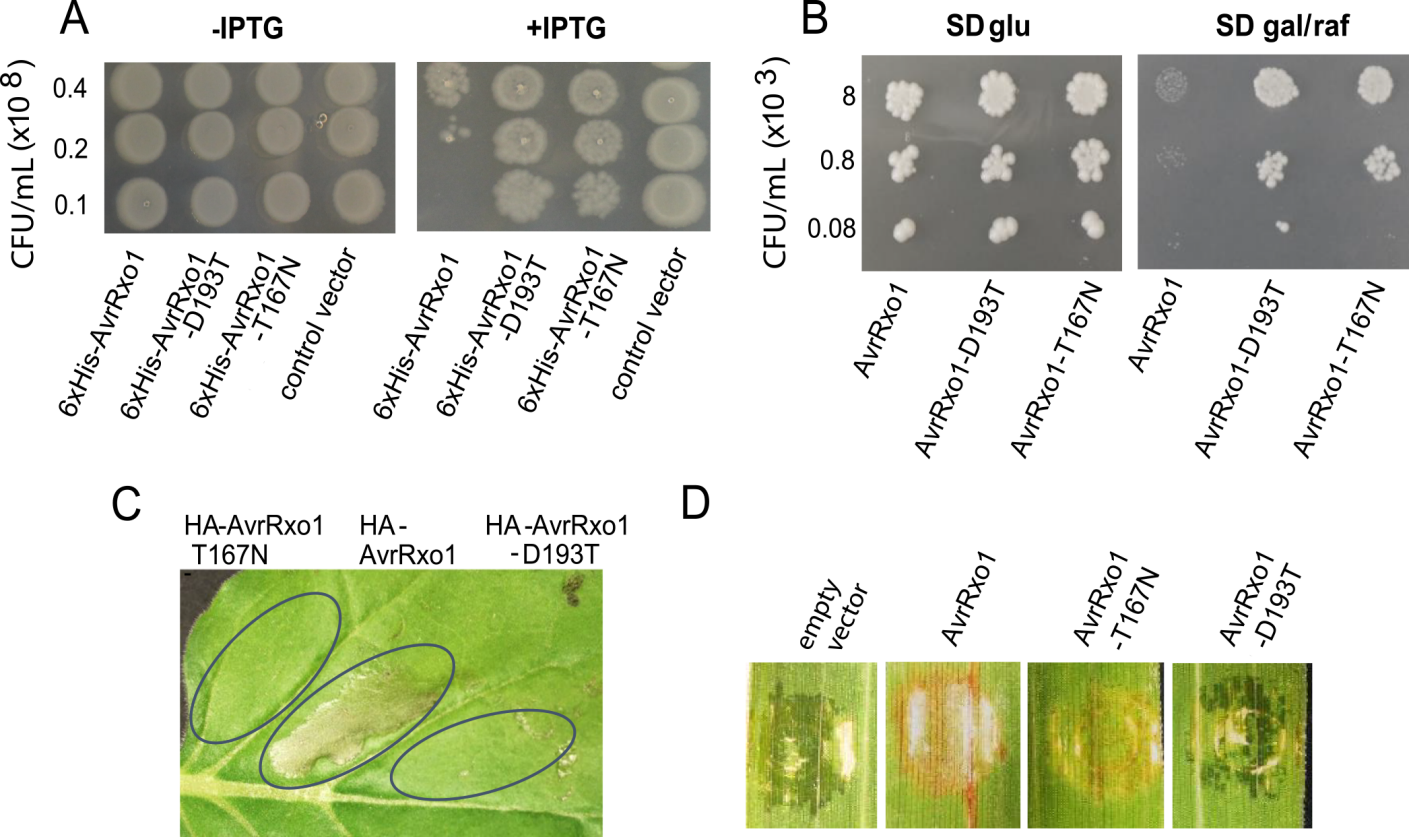
Requirement of predicted catalytic residues of AvrRxo1 for toxic or growth-suppressive effects in prokaryotes and eukaryotes and for induction of HR in *Rxo1* rice. (A) *E*. *coli* transformed with pDEST-AvrRxo1, pDEST-AvrRxo1-D193T, pDEST-AvrRxo1-T167N, and pDESTcv grown on (-IPTG) and inducing (+IPTG) media. (B) *S*. *cerevisiae* transformed with pESC-Trp-DEST-AvrRxo1 and catalytic site mutant derivatives pESC-Trp-DEST-D193T and -T167N on repressing (SD media amended with 2% glucose) and inducing (SD media amended with 2%/1% galactose/raffinose) media. (C) *N*. *benthamiana* leaf during transient *Agrobacterium*-mediated expression of HA-AvrRxo1, HA-AvrRxo1-D193T and HA-AvrRxo1-T167N. Leaf was imaged 50 hours after infiltration. Expression analysis shown in S1 Fig. (D) Leaves of transgenic rice variety Kitaake expressing Rxo1 were inoculated with derivatives of *X*. *oryzae* strain X11-5A carrying pHM1-AvrRxo1, pHM1-AvrRxo1-D193T, pHM1-AvrRxo1-T167N, or the empty vector pHM1. Appearance of water-soaking lesions or HR were recorded 5 days post infiltration.

We previously found that growth suppression by AvrRxo1 was more pronounced in *E*. *coli* liquid cultures induced from a lower initial density than from a higher density [5], suggesting that toxicity is reduced above a threshold cell density, as was observed in a zeta toxin [15]. For a sensitive comparison of bacteriostasis by site-directed AvrRxo1 mutants in *E*. *coli*, we assayed for AvrRxo1 and catalytic site mutant growth suppression at multiple cell densities on solid media. 6xHis-AvrRxo1, but not T167N and D193T mutant derivatives, abolished *E*. *coli* growth when expression was induced below the density of 2X10^7^ CFU/mL, but not at 4X10^7^ CFU/mL or above (Fig 1A). AvrRxo1 expression similarly inhibited growth of *Saccharomyces cerevisiae* in a manner dependent on the two catalytic residues; here growth suppression was obvious at all densities tested (Fig 1B). Previous work demonstrated that transient expression of YFP-tagged AvrRxo1 induced a toxic-like watersoaking and cell collapse phenotype in several plant species, and that this was abolished by the T167N mutation [8]. We found that transient expression of HA-tagged AvrRxo1 triggered toxic cell collapse by 50 hours post-infiltration (hpi) in leaves of *N*. *benthamiana*, and both the T167 and D193 residues were necessary for this phenotype (Fig 1C). Expression of the catalytic mutants was confirmed using Western blot analysis (S1 Fig); although expression of HA-AvrRxo1 could not be detected, possibly due to cell death associated with expression.

We next investigated whether residues D193 and T167 were required for triggering Rxo1-mediated resistance in rice. We and others have been unable to obtain a knockout mutant of *avrRxo1* from the model *X*. *oryzae* pv. *oryzicola* strain BLS256 in repeated attempts over many years [7], perhaps because of its function as part of a TA system and addiction module. Therefore, *avrRxo1*:*arc1* gene module and mutant derivatives were introduced into the weakly pathogenic *X*. *oryzae* strain X11-5A on the low-copy cosmid vector pHM1. X11-5A has an *arc1* antitoxin gene in its chromosome, allowing *avrRxo1*:*arc1* to be introduced without detrimental growth effects. At five days post infiltration (dpi) into leaves of Kitaake *Rxo1* transgenic rice, vector control X11-5A (pHM1) caused watersoaking, while X11-5A (p*avrRxo1/* pHM1-AvrRxo1) inoculation sites were characterized by brown color development and absence of watersoaking, characteristic of the Rxo1 resistance response (Fig 1D). X11-5A (pHM1-AvrRxo1-D193T) caused a watersoaking phenotype similar to the control, indicating that the catalytic aspartate is necessary for triggering Rxo1. However, the X11-5A (pHM1-AvrRxo1-T167N) also exhibited a browing phenotype and did not show signs of watersoaking (Fig 1D). This finding, in addition to our previous observation that the T167N mutant retains virulence enhancement of *X*. *oryzae* in rice [8], suggests that the T167N mutation uncouples AvrRxo1 toxicity from immune triggering or suppressing functions. A western blot confirmed expression of AvrRxo1 and catalytic site mutants during rice infection (S2 Fig). Unlike during expression in tobacco, AvrRxo1-T167N accumulated to lower levels than AvrRxo1-D193T or AvrRxo1, which may affect the phenotype of AvrRxo1-T167N in rice.

### Accumulation of 3’-NADP is the major metabolic change induced by expression of AvrRxo1 in *E*. *coli* and *Saccharomyces cerevisiae*

**Fig 2:**
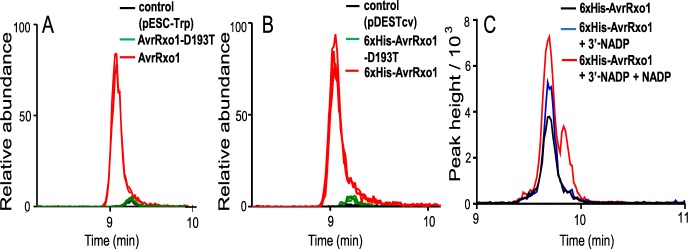
Accumulation of 3’-NADP is the major metabolite change elicited by AvrRxo1 expression in *E*. *coli* and *S*. *cerevisiae*. (A-B) Extracted ion LC-MS chromatograms of the NADP parent ion, m/z 742.067, in negative ion mode in yeast (A) and bacteria (B) expressing either AvrRxo1, AvrRxo1-D193T, or the relevant control. *S*. *cerevisiae* strains analyzed in (A) carried pESC-TRP-DEST-AvrRxo1, -AvrRxo1-D193T, or pESC-TRP. *E*. *coli* strains analyzed in (B) carried pDEST-AvrRxo1, pDEST-AvrRxo1-D193T, or pDESTcv. Biological replicates, n = 3, are overlaid. (C) Extracted ion LC-MS chromatogram of NADP in a representative AvrRxo1-expressing bacterial extract from (B), unspiked, spiked with 3’-NADP, or spiked with both 3’-NADP and NADP.

We used untargeted metabolomics to uncover small molecule targets of ectopically expressed AvrRxo1 in bacteria and yeast. Having demonstrated that a substitution in the predicted substrate-binding residue D193T abolishes diverse known phenotypes of AvrRxo1, we selected AvrRxo1-D193T as the inactivated AvrRxo1 control for the study along with the control vectors used in Fig 1A and 1B. AvrRxo1 expression in *E*. *coli* cultures was induced at a density well above the threshold for discernible growth repression as observed in Fig 1A, to minimize off-target effects of AvrRxo1 toxicity on the metabolome. Triplicate cultures were subjected to metabolic profiling using zwitterionic-hydrophilic interaction liquid chromatography (ZIC-HILIC) coupled to time-of-flight mass spectrometry (TOF-MS). Analysis revealed that AvrRxo1 expression affected bacterial and yeast central metabolism in distinct but overlapping ways. In both organisms, the most dramatic AvrRxo1-dependent change was the strong accumulation of a single peak annotated as NADP (Fig 2A and 2B. S1 File). Other effects, some of which were specific to *E*. *coli*, included modest (1.5 to 2-fold) increases in relative abundance of predicted metabolites involved in the TCA cycle (citric acid, succinic acid, malic acid, and pantothenic acid), in some amino acids (aspartic acid, lysine, leucine/isoleucine, phenylalanine, tyrosine, and tryptophan), in glutathione, and in pyrimidine nucleotides. Heatmaps summarizing relative changes in abundance of 79 annotated metabolites are found in Supplemental S3 and S4 Figs, and boxplots of individual metabolites are found in Supplemental s1 File. In both organisms, some metabolite changes were observed in both AvrRxo1 and AvrRxo1-D193T (S3 Fig and S4 Fig), suggesting that AvrRxo1 can exert some metabolic changes in a manner independent of the catalytic aspartic acid. For example, the NADP precursor NAD decreased in relative abundance as a result of expression of both AvrRxo1 and AvrRxo1-D193T in *E*. *coli*, but increased slightly in abundance during the expression of both treatments in yeast (pg. 12 of S1 File). We also profiled tissues of Kitaake rice 48 hours after inoculation with *X*. *oryzae* strain X11-5A carrying *avrRxo1*:*arc1* and the empty vector pHM1, but no significant differences were observed (S1 File).

Given that a large accumulation of NADP observed upon AvrRxo1 expression in yeast and bacteria would be unlikely due to metabolic turnover, spiking experiments were performed to determine whether the annotation was accurate. Lysates from AvrRxo1-expressing *E*. *coli* cells were amended with commercial NADP and subjected to TOF-MS/MS analysis. NADP formed a distinct peak from the AvrRxo1-dependent metabolite, with a retention time roughly 10s longer (S5A and S5B Fig). However, the mass spectra of NADP and the AvrRxo1-dependent metabolite were identical (S5C and S5D Fig) indicating that the peak represented a close analog of NADP. Interpretation of the MS/MS fragmentation pattern suggested that the most probable NADP analog that would generate a nearly identical spectrum would be 3’-NADP. Spiking of AvrRxo1-expressing *E*. *coli* lysates with commercially available 3’-NADP standard or simultaneously with both NADP isomers confirmed that the AvrRxo1-dependent compound has an identical retention time to 3’-NADP (Fig 2C). In addition, the AvrRxo1-dependent molecule was a spectral match to the 3’-NADP standard, confirming its identity as 3’-NADP (S6 Fig). Thus, AvrRxo1 expression in yeast and *E*. *coli* results in a strong accumulation of 3’-NADP, and not in other polar metabolites detectable through the ZIC-HILIC method. To determine whether AvrRxo1 mediates 3’-NADP accumulation through direct phosphorylation of NAD, 6xHis-AvrRxo1 and 6xHis-AvrRxo1-D193T were purified and tested for NAD kinase activity in an *in vitro* assay (S7 Fig). 3’-NADP accumulated in the presence of 6xHis-AvrRxo1 (S7C Fig) but not in the presence of 6xHis-AvrRxo1-D193T (S7D Fig), confirming that AvrRxo1 is an NAD kinase dependent on the D193 catalytic site.

### 3’-NADP accumulates upon AvrRxo1 expression *in planta* and during infection with *X*. *oryzae* pv. *oryzicola*

**Fig 3:**
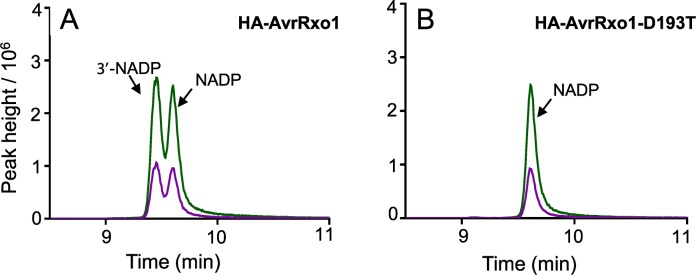
AvrRxo1 produces 3’-NADP upon transient expression in *N*. *benthamiana*. (A-B) LC-MS/MS analysis of NADP from leaves of *N*. *benthamiana* transiently expressing HA-AvrRxo1 (A) or HA-AvrRxo1-D193T (B). *N*. *benthamiana* leaf samples were collected 36 hours post infiltration (hpi), prior to visible cell collapse symptoms. Similar results obtained for 3 independent experiments.

AvrRxo1 is a T3E that has been associated with virulence enhancement and suppression of nonhost resistance when expressed from *X*. *oryzae*, and with phytotoxic cell collapse when transiently expressed *in planta* [8, 9]. To determine whether AvrRxo1 phosphorylates NAD in plant cells, HA-AvrRxo1 and HA-AvrRxo1-D193T were transiently expressed in leaves of *N*. *benthamiana*. Leaf discs were collected at 36 h after Agroinfiltration, prior to the initial onset of watersoaking symptoms in AvrRxo1-expressing leaves. Both NADP and 3’-NADP were detected by LC-MS/MS in the AvrRxo1-expressing leaves (Fig 3A), but only NADP was detected in leaves infiltrated with the AvrRxo1-D193T construct (Fig 3B).

**Fig 4:**
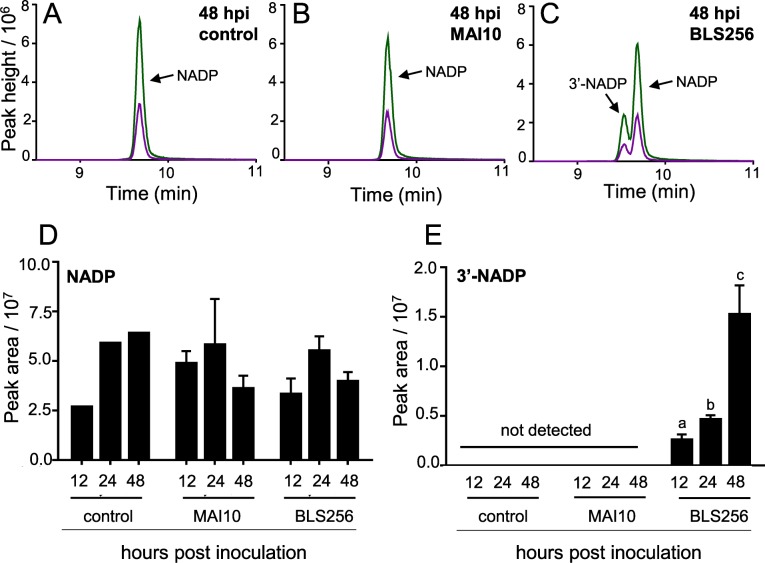
3’-NADP accumulates in rice leaves upon inoculation with a naturally *avrRxo1*-expressing strain of *X*. *oryzae*, but not with an *avrRxo1*-deficient strain. (A-C) LC-MS/MS analysis of rice leaves 48 h after infiltration with water (A), the *avrRxo1-*negative strain *Xanthomonas oryzae* pv. *oryzicola* MAI10 (B), or the *avrRxo1-*positive strain BLS256 (C). (D-E) Relative peak area of NADP (D) and 3’-NADP (E) in three biological replicates (means ± SEM, n = 3). Letters denote treatments statistically different from the 12h timepoint (p<0.05).

We next tested whether *X*. *oryzae* pv. *oryzicola* (*Xoc*), the pathogen that produces AvrRxo1, can generate 3’-NADP during the infection of rice by wild-type *X*. *oryzae*. Because of our aforementioned difficulty in inactivating *avrRxo1*, we were unable to generate a deletion mutant in BLS256 mutant to conclusively determine that *avrRxo1* is the sole source of 3’-NADP during infection. However, some leaf streak-causing *Xoc* isolates from Africa naturally lack *avrRxo1*. Profiling of diverse *Xoc* strains has shown that *avrRxo1* gene presence or absence is tightly linked to the phenotypes of Rxo1 activation and suppression of tobacco nonhost resistance [17]. The *Xoc* strain MAI10 was used as an *avrRxo1*-negative leaf streak pathogen in this study. MAI10 is a close genetic relative of BLS256 that produces similar symptoms and encodes a nearly identical complement of non-TAL effector genes other than *avrRxo1*, although it differs in TAL effector composition [17–19]. Leaves of rice variety Kitaake were infiltrated with strain MAI10, BLS256 or sterile water, and samples were collected and analyzed at 12, 24 and 48 hpi. 3’-NADP was detected in samples inoculated with BLS256, but not with MAI10 or a water control (Fig 4A–4C). To confirm that *avrRxo1* is the key genetic factor conferring the difference in 3’-NADP accumulation between BLS256 and MAI10, we introduced pHM1-AvrRxo1 and pHM1 into MAI10 via triparental mating. A very low but distinct 3’-NADP signal was detected in three out of four replicates of the inoculations with MAI10 (pHM1-AvrRxo1), but not in inoculations with MAI10 alone or MAI10 (pHM1) (S8 Fig). These results demonstrate that AvrRxo1 NAD kinase activity occurs during *Xoc* infection of rice.

3’-NADP was detectable within 12 hpi of infection with BLS256, with relative abundance increasing through 48 hpi (Fig 4D and 4E, S9 Fig). Unlike the heterologous expression conditions in which 3’-NADP accumulates to a very high relative abundance, relative peak areas of 3’-NADP were far smaller than those of native NADP in rice tissues within 48h. This is consistent with the lack of observable increase in the NADP/3’-NADP peak identified in our initial metabolomic profile in infected rice. Although this experiment does not conclusively prove that the source of the 3’-NADP was the rice and not and intercellular *X*. *oryzae*, it is well-established that AvrRxo1 is introduced into and active in rice cells during infection [20], and that AvrRxo1 activity is strongly inhibited in bacteria, *in vitro*, and *in planta* in the presence of the antitoxin Arc1 [8, 16]. We have not been able to introduce an expressed *avrRxo1* gene into *X*. *oryzae* strains in the absence of the *arc1* gene, thus it is presumed that free AvrRxo1 is toxic to bacteria and thus is likely inactivated in BLS256 cells. Although NADP and 3’-NADP are both synthesized from NAD, there was no significant difference in total (2’)-NADP levels associated with the presence of *avrRxo1* during *X*. *oryzae* pathogenesis on rice (Fig 4A–4C). Together, these results demonstrate that AvrRxo1 catalyzes 3’-NADP production in plants and that 3’-NADP is produced early in bacterial leaf streak infection.

### The toxicity-deficient Walker motif mutant AvrRxo1-T167N generates 3’-NADP in yeast and retains ability to suppress the flg22-induced oxidative burst in plants

In previous work, we demonstrated that AvrRxo1 enhances early proliferation of pathogenic *Xanthomonas* in rice leaves. The T167N substitution in the Walker motif abolished growth suppression in *E*. *coli* and cell collapse in plants, but still retained AvrRxo1 virulence enhancement on rice [8] and recognition by Rxo1 (Fig 1D). Because the Walker A threonine has been reported essential for the activity of other Pnk domain kinases [21], we initially hypothesized that the Walker site mutant may be catalytically inactive but retain selected phenotypes due to retention of an intact substrate-binding site, perhaps due to sequestration of the hypothetical substrate in the cell [8]. Alternatively, AvrRxo1 could be recognized structurally by Rxo1. In light of AvrRxo1 3’ NAD kinase activity, we decided to revisit the activity of the T167N mutant. Specifically, we wanted to determine whether the T167N substitution uncouples NAD kinase activity from immune suppression or recognition in the plant.

**Fig 5:**
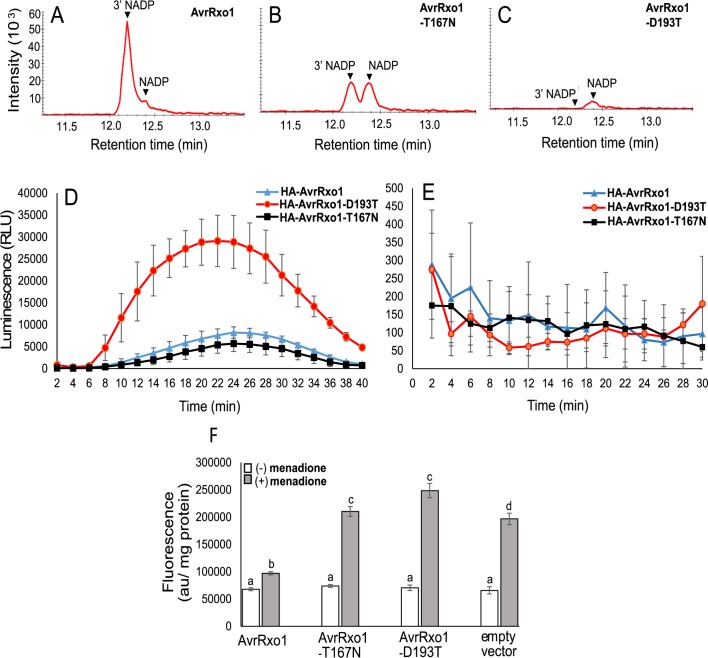
AvrRxo1-T167N retains low NAD kinase activity that allows suppression of flg22-induced oxidative burst in *N*. *benthamiana*, but not menadione induced oxidative burst in yeast. (A-C) LC-MS/MS analysis of NADP from yeast cells expressing either AvrRxo1 (A), AvrRxo1-T167N (B) or AvrRxo1-D193T (C). (D-E) Flg22 (1 μM) (D) or water (mock) (E) induced ROS burst measured in leaf discs of *N benthamiana* transiently expressing either HA-AvrRxo1, HA-AvrRxo1-D193T or HA-AvrRxo1-T167N. Figure represents mean data of one independent experiment with three technical replicates (mean ±SD; n = 3). Similar results were seen in three independent experiments. Statistical analysis was performed using *t*-test for values corresponding to maximum response. (F) Menadione-induced intracelluar ROS production measured in yeast cells carrying pESC-TRP-AvrRxo1, -AvrRxo1-T167N, -AvrRxo1-D193T or the vector pESC-TRP in inducing media. Values were normalized to protein concentration. Figure represents mean data of one independent experiment with four technical replicates (mean ±SD; n = 4). Similar results were seen in three independent experiments.

We first determined whether AvrRxo1-T167N could produce 3’-NADP in yeast. Induced culture lysates of yeast strains depicted in Fig 1B were subjected to metabolic profiling using HILIC-LC-MS/MS. AvrRxo1-T167N generated detectable 3’-NADP in yeast, although the level was low compared to AvrRxo1 (Fig 5A–5C), accumulating roughly in proportion to NADP. Thus, yeast expression of AvrRxo1-T167N retains a level of kinase activity that is insufficient to cause visible toxicity.

In addition to enhancing virulence, AvrRxo1 is a suppressor of flg22-mediated innate immunity in Arabidopsis [9, 22]. We determined the impact of transient AvrRxo1 expression on generation of the PAMP-triggered burst of reactive oxygen species (ROS), a critical early event in plant defense signaling. The initial apoplastic oxidative burst is largely generated by plasma membrane NADPH oxidases from NADPH or NADH in the cytoplasm [23], where AvrRxo1 is also localized [22]. NAD and NADP are also thought to have signaling roles in generation of the oxidative burst [24, 25], and therefore we hypothesized that AvrRxo1 activity might suppress ROS formation. In a luminol-HRP based chemiluminescence assay performed on leaves prior to the onset of cell collapse, HA-AvrRxo1 transient expression in *N*. *benthamiana* strongly suppressed flg22-induced ROS accumulation in comparison with HA-AvrRxo1-D193T (Fig 5D). AvrRxo1-T167N also strongly suppressed ROS accumulation (Fig 5D), while no signal was detected in mock-induced leaves (Fig 5E). While this is not a quantitative comparison of HA-AvrRxo1 and HA-AvrRxo1-T167N ROS suppression efficacy- HA-AvrRxo1 was not detected by Western Blot, preventing comparison of expression levels with HA-AvrRxo1-T167N (S1 Fig)- these results demonstrate that AvrRxo1 suppresses the PAMP-triggered ROS burst even in a mutant that generates subtoxic levels of AvrRxo1 activity.

Finally, we asked whether AvrRxo1 and the catalytic site mutants were able to suppress ROS generation in other eukaryotes, or whether ROS suppression was specific to the flg22-induced burst in plants. Menadione is a redox cycling agent that stimulates ROS production from mitochondrial NAD(P)H in yeast [26]. In a fluorescence assay, AvrRxo1 expression reduced levels of menadione-induced ROS compared with AvrRxo1-D193T. AvrRxo1-T167N did not suppress yeast ROS levels (Fig 5F). These findings demonstrate that AvrRxo1 is a suppressor of multiple mechanisms of ROS generation in diverse eukaryotes, and determined that although the mutant AvrRxo1-T167N can suppress the flg22-mediated ROS burst in plants, it does not suppress mitochondrial ROS accumulation in yeast.

## Discussion

AvrRxo1 is part of an ancient family of bacterial toxins co-opted by the type III secretion system for secretion into plants. This study demonstrates that AvrRxo1 phosphorylates NAD in eukaryotic cells, and that a catalytic site necessary for this kinase function is required for the toxic and immune-triggering functions of AvrRxo1. NAD is a coenzyme and redox carrier universally essential for metabolic function, and host metabolism is a common target for pathogens. In human pathogenic bacteria, glycohydrolase domain toxins metabolically poison target host or microbial cells by degrading NAD, gaining access via cytolysin-mediated translocation or through the type VI secretion system [27, 28]. Pathogens of plants and animals are known to affect diverse host primary metabolites through metabolic crosstalk or manipulation of host transcription and signaling [29, 30]. A few type III secreted effectors directly target host small molecules, including sugars and glutathione [3, 4], challenging the former paradigm of effectors as protein modifiers. This work demonstrates that a type III secreted effector can phosphorylate a universally essential cofactor in the host. The direct modification of a universal metabolic player and redox carrier such as NAD represents a novel pathogen strategy for manipulation of host function.

AvrRxo1 exhibits toxicity upon ectopic (*i*.*e*., high-level) expression in bacteria, yeast, and plants, and these phenotypes are abolished upon mutation of the D193 or T167 catalytic sites. The drastic accumulation of 3’-NADP in yeast and bacteria, and the lack of detection of any other novel AvrRxo1-dependent metabolites in the global metabolomic profile, suggests that NAD is a primary small molecule target of AvrRxo1 function. One putative avenue for AvrRxo1 toxicity is enzyme inhibition by its product 3’-NADP. The AvrRxo1 structural homolog PezT, which phosphorylates UDP-GlcNac to generate a non-native analog, exerts toxicity both through UDP-GlcNac depletion and through inhibition of the UNAG-utilizing enzyme MurA by the phosphorylated product [31]. Unlike UNAG, NAD and NADP are required for hundreds of essential reactions, and therefore determining the *in planta* enzyme inhibition effects of 3’-NADP may not be as straightforward. Accumulation of 3’-NADP upon AvrRxo1 expression in bacterial and yeast cells suggests that 3’-NADP is not an enzyme substrate, nor is it turned over rapidly in these organisms. Early biochemical studies found little indication of 3’-NADP utilization or competitive inhibition of numerous central NADP-specific enzymes from various organisms, including the dehydrogenases of the pentose phosphate pathway, isocitrate dehydrogenase, glutathione reductase, and NADP phosphatases and transhydrogenases [32–35].

AvrRxo1 might also confer toxicity through depletion or sequestration of its substrate, NAD, and a resulting alteration in NAD(H) homeostasis and redox environment. We did not observe changes in relative NAD abundance that were dependent on catalytically active AvrRxo1 in bacteria, yeast, or infected rice. However, due to tightly maintained homeostasis of NAD levels, experimental manipulation of NAD synthesis or turnover often does not elicit measurable changes in NAD abundance, but often rather affects the rate of NAD metabolism [36, 37]. Therefore, the lack of association of NAD levels with AvrRxo1 activity does not rule out NAD depletion as a mechanism of immune suppression. Profiling of diverse organisms chemically or genetically manipulated to alter NAD production have frequently reported effects on abundance of metabolites pertinent to NAD-related functions, notably TCA cycle intermediates, nucleotides, and glutathione species [36–40]. These trends are consistent with the profile of metabolites modestly affected by AvrRxo1 expression in yeast and bacteria (S1 File, S3 Fig, S4 Fig). Enhancement of NAD consumption or inhibited NAD production is usually associated with a decreased abundance of these metabolites, and vice-versa [36–39], so it is interesting that the NAD-modifying enzyme AvrRxo1 caused them to increase in our study. AvrRxo1 metabolic effects were studied here in conditions under which no toxic effects were observable (i.e., early timepoints or high cell densities), and may only reflect early events in AvrRxo1 metabolic modification. NAD(H) quantification and assessment of metabolic flux and redox changes during the process of AvrRxo1 intoxication, as well as during immune suppression, will be needed to determine how metabolic perturbation by AvrRxo1 affects growth suppression or cell collapse.

Expressed from a low-copy vector in *X*. *oryzae* on rice, AvrRxo1 shows no signs of toxicity or of major metabolic remodeling (S1 File), but it does exhibit several classic phenotypes of effectors in the plant infection process, including suppression of PTI, triggering of ETI, and suppression of a nonhost HR [8, 9]. The Walker A site mutant AvrRxo1-T167N, which has no growth suppressing or cell collapse phenotype, retains an ability to trigger HR, suppress ROS, and enhance pathogen proliferation (summarized in Table 1). In *N*. *benthamiana*, this mutation uncouples the toxic effects of AvrRxo1 from plant immune functions, suggesting that the latter may be more sensitive to subtoxic levels of AvrRxo1 activity. As with toxicity, plant immune functions could be modulated through NAD utilization and/or through generation of 3’-NADP. AvrRxo1 is localized to the plant cytoplasm [9, 22], which is thought to contain a small fraction of the tightly compartmentalized NAD in the eukaryotic cell [41, 42]. Cytoplasmic NAD is an critical early signal in plant defense against diverse pathogens that is required for the PAMP-induced ROS burst and activation of stomatal immunity [43], salicyclic acid signalling, and callose deposition [25]. Manipulation of NAD metabolism by AvrRxo1 could disrupt a delicate redox balance key for NAD function in early defense signaling [24]. The observation that AvrRxo1-T167N inhibited the cytoplasmic NAD(P)H-derived ROS burst in plants, but not the mitochondria-localized ROS burst in yeast, would be consistent with the hypothesis that the smaller cytoplasmic pool of pyridine nucleotides is especially sensitive to subtoxic levels of AvrRxo1 activity. NAD is also required for production of the NADPH and cyclic ADP-ribose that function in the oxidative burst and calcium signaling, and for posttranslational defense signaling by sirtuins and poly-ADPribosyltransferases [24].

**Table 1:** Phenotypes of AvrRxo1 and catalytic site mutants.

	Phenotype	AvrRxo1	AvrRxo1 (D193T)	AvrRxo1 (T167N)
Activity	3’-NADP accumulation *in vitro*	+	-	NT
	3’-NADP accumulation in *E*. *coli*	+ (>>NADP)^a^	-	NT
	3’-NADP accumulation in yeast	+ (>>NADP)	-	+ (≈ NADP)
	3’-NADP accumulation in transient *Nb* expression	+ (≈ NADP)	-	NT
	3’-NADP accumulation during *Xoc* infection	+ (<NADP)	-	NT
Toxic Effect	*E*. *coli* growth suppression	+	-	-
	Yeast growth suppression	+	-	-
	Plant cell collapse upon transient expression	+	-	-
Host Immune Effects	Activation of Rxo1-mediated defense	+	-	+
	Enhancement of *Xo* proliferation on rice	+	NT	+ [8]
	Suppression of flg22-mediated ROS burst in plants	+	-	+
	Suppression of menadione-induced ROS in yeast	+	-	-

+: exhibits the respective phenotype; -: lacks the respective phenotype; NT: not tested

^a^ indicates the relative abundance of 3’-NADP in comparison with NADP

3’-NADP could also be a critical inhibitor of the flg22-mediated ROS burst. 3’-NADP did not inhibit many central respiratory enzymes in older studies [32], but was later found to effectively inhibit maize NADP-malic enzyme in a noncompetitive fashion [44]. NADP-malic enzyme is a cytosolic electron donor that donates the electrons necessary for the defense-related oxidative burst in plants; the *Magnaporthe oryzae* effector AVR-Pii targets the rice malic enzyme Os-NADP-ME2 to inhibit the ROS burst [45]. Another logical ROS-blocking mechanism of 3’-NADP could be the inhibition of synthesis of NAADP from NADP by ADP-ribosyl cyclases. NAADP is a second messenger involved in calcium release, but its synthesis is not well characterized in plants. AvrRxo1 was also recently found to phosphorylate the NAD precursor, NAAD, *in vitro* at a similar efficiency to NAD, generating the product 3’ NAADP [16]. NAAD is a very low-abundance metabolite and we did not detect evidence of 3’ NAADP accumulation through global metabolome profiling, but this does not rule out NAAD as a potentially relevant target of AvrRxo1-induced phenotypes.

Perhaps the most intriguing question arising from this work is that of how AvrRxo1 triggers activation of resistance mediated by the canonical NB-LRR R protein Rxo1. The lack of a phenotype in the D193T mutant suggests that AvrRxo1 kinase activity is required for Rxo1 activation. However, bacterial avirulence effectors are generally recognized through their activity on a protein target. The finding that AvrRxo1 modifies a small molecule prompts many questions on the nature of resistance activation- might the recognized signal be 3’-NADP, a change in the redox environment, or a change in NAD-dependent post-translational modification of proteins? By establishing NAD as an effector phosphorylation target during infection, this work demonstrates that direct “cofactor engineering” may be an effective strategy for pathogen manipulation of the host- and possibly, an effective signal for host recognition of the pathogen.

## Materials and methods

### Strains and culture conditions

The bacterial strains used in this study included *E*. *coli* DH5α for plasmid maintenance, *E*. *coli* BL21 (DE3) for protein expression, *Agrobacterium tumefaciens* strain GV2260 for transient expression in *N*. *benthamiana*, *Xanthomonas oryzae* pv. *oryzicola* strains BLS256 and MAI10 for infection of rice plants, and *Xanthomonas oryzae* strain X11-5A for detection of the Rxo1- mediated HR. *Saccharomyces cerevisiae* strain Y2H gold (TakaraBioUSA, Mountain View, CA) was used for AvrRxo1 expression.

*E*. *coli* strains were routinely cultured in Luria Bertani (LB) medium at 37° C unless stated otherwise and *A*. *tumefaciens* strain GV2260 was grown in YEB medium at 28° C with appropriate antibiotics. *X*. *oryzae* pv. *oryzicola* strains BLS256 and MAI10 and *Xanthomonas oryzae* strain X11-5A were grown on peptone sucrose agar (PSA) at 28° C. *Saccharomyces cerevisiae* was grown in SD (Synthetic defined) media lacking tryptophan and containing either glucose (2%) or galactose (2%) or galactose/raffinose (2%/1%).

### Plasmid construction and transformation

Plasmids used in this study are listed in Table 2. For protein expression studies in *E*. *coli*, previously published pDEST527-derived clones were used for expression of 6xHis-AvrRxo1 and the catalytic site mutants 6xHis-AvrRxo1-D193T and 6xHis-AvrRxo1-T167N [8]. pDESTcv, used as a second control strain for pDEST studies, replaces the toxic Gateway cassette with the first 300 nt of the 5’ end of *avrRxo1;* this construct was previously shown to lack any growth suppression phenotype in bacteria [5]. For bacterial assays in rice, pAvrRxo1 (also referred to as pHM1-AvrRxo1) [7], pHM1-AvrRxo1-T167N [8] and pHM1-AvrRxo1-D193T (this study) were used with pHM1 (R. Innes, Indiana University) as the empty vector control. For construction of pHM1-AvrRxo1-D193T, the plasmid pAvrRxo1 [7] was used as a template for amplification of SacI-PstI flanked *avrRxo1*:*arc1* region using the primers avrRxo1:arc1for, caccgagctcgacgcatttttatagcttcgttcg, and avrRxo1:arc1rev, gatcctgcagacagaggactcggattgaaccagt. The fragment was cloned into pENTR-D-Topo (Invitrogen). A single-site substitution was introduced using primers pHM1AvrRxo1D193Tfor, gggaaaggttgcgtgaatccaactgtattcaagagttcgcttgcg, and pHM1AvrRxo1-D193Trev, cgcaagcgaactcttgaatacagttggattcacgcaacctttccc, in a mutagenic PCR designed according to the manufacturer’s instructions for QuikChange II Site-Directed Mutagenesis Kit (Agilent), to generate pENTR-D-Topo-pAvrRxo1-D193T. Mutagenesis was confirmed by sequencing. The SacI-PstI fragment from vector pENTR-D-Topo-pAvrRxo1-D193T was cloned into pHM1. Inserts of all pHM1 and Gateway constructs were sequenced to confirm that *avrRxo1*-containing inserts were identical except for the single site mutations. pHM1 constructs were introduced into *X*. *oryzae* strain X11-5A by electroporation as previously described [7], and into MAI10 by triparental mating as previously described [46]. For *Agrobacterium*-mediated transient assays, entry vector pENTR-D-Topo clones carrying wild type *avrRxo1*, *avrRxo1-D193T* and *avrRxo1-T167N* [8] were used for recombination of the desired inserts into the Gateway compatible destination vector pEARLEYGATE 201 [47] using LR clonase according to manufacturer’s instructions (Invitrogen, Carlsbad, CA). The plasmids were transformed into *A*. *tumefaciens* via electroporation. For inducible expression in yeast, the Gateway destination vector pESC-Trp-DEST was constructed by cloning the SalI fragment of destination cassette C.1 (Gateway Vector Conversion System, ThermoFischer Scientific) into the MCS2 of pESC-TRP (Agilent). Wild type *avrRxo1*, *avrRxo1-T167N*, and *avrRxo1-D193T* were recombined into pESC-Trp-DEST vector from the corresponding pENTR-D-Topo clones [8] via LR clonase reactions; the incorporated stop codon prevents expression of the C-terminal Myc tag. The plasmids were transformed into yeast using the Frozen-EZ Yeast Transformation Kit (Zymo Research, Irvine, CA) according to manufacturer’s instructions.

**Table 2:** List of plasmids used in this study.

Plasmid	Relevant characteristics and use	Source
pENTR/D-Topo	Entry vector for Gateway cloning technology, Km^R^	Invitrogen
pENTR/D-Topo-AvrRxo1	*avrRxo1* from *X*. *oryzae* pv. *oryzicola* strain BLS256 cloned into pENTR/D-topo, Km^R^	[8]
pENTR/D-Topo-AvrRxo1-D193T	pENTR/D-Topo-AvrRxo1 with D193T substitution, Km^R^	[8]
pENTR/D-Topo-AvrRxo1-T167N	pENTR/D-Topo with T167N substitution, Km^R^	[8]
pDEST-527	Gateway destination vector for *E*. *coli* expression, T7 promoter, N-terminal 6xHis tag, Amp^R^, Cm^R^	Addgene.org
pDEST527-AvrRxo1	*avrRxo1* from *X*. *oryzae* pv. *oryzicola* strain BLS256 in pDEST527, N terminal 6xHis tag, Amp^R^	[8]
pDEST527-AvrRxo1-D193T	*avrRxo1-D193T* in pDEST527, N terminal 6xHis tag, Amp^R^	[8]
pDEST527-AvrRxo1-T167N	*avrRxo1-T167N* in pDEST527, N terminal 6xHis tag, Amp^R^	[8]
pDEST527-cv	pDEST-527 with Gateway cassette replaced by a 300 nt fragment of the 5’ end of *avrRxo1*	[5]
pHM1	Broad-host range cosmid vector, Sp^R^	R. Innes, Indiana University
pavrRxo1 (also referred to as pHM1-AvrRxo1)	The 1.797-kb SphI fragment subcloned into pHM1 from VB1C that contains the avrRxo1and arc1 genes, Sp^R^	[7]
pHM1-AvrRxo1-T167N	pHM1-AvrRxo1 with T167N substitution, Sp^R^	[8]
pENTR/D-Topo-pAvrRxo1	SacI-PstI flanked insert from pavrRxo1 in pENTR/D-topo, Km^R^	This study
pENTR/D-Topo-pavrRxo1-D193T	SacI-PstI fragment from pHM1-avrRxo1 with D193T substitution in pENTR/D-Topo Km^R^;	This study
pHM1-AvrRxo1-D193T	SacI-PstI fragment of pENTR/D-Topo-pAvrRxo1-D193T in pHM1; Sp^R^	This study
pEARLEYGATE 201	Gateway destination vector for binary expression with N-terminal HA tag, CaMV 35S promoter, Km^R^	[47]
pEARLEYGATE 201-AvrRxo1	insert from pENTR/D-Topo-AvrRxo1 in pEARLEYGATE201 Km^R^;	This study
pEARLEYGATE 201-AvrRxo1-D193T	insert from pENTR-D-Topo-AvrRxo1-D193T in pEARLEYGATE201, Km^R^;	This study
pEARLEYGATE 201-AvrRxo1-T167N	insert from pENTR-D-Topo-AvrRxo1-T167N in pEARLEYGATE201, Km^R^;	This study
pESC-TRP	pUC plasmid origin, auxotrophic marker gene *TRP1*, GAL1 and GAL10 promoter, Amp^R^; protein expression studies in *S*. *cerevisiae*	Agilent
pESC-TRP-DEST	Sal fragment from destination cassette C.1 (Gateway Vector Conversion System) in pESC-TRP	This study
pESC-TRP-DEST-AvrRxo1	insert from pENTR/D-Topo-AvrRxo1 in pESC-TRP-DEST, Amp^R^	This study
pESC-TRP-DEST-AvrRxo1-D193T	insert from pENTR/D-Topo-AvrRxo1-D193T in pESC-TRP-DEST, Amp^R^	This study
pESC-TRP-DEST-AvrRxo1-T167N	insert from pENTR/D-Topo-AvrRxo1-T167N in pESC-TRP-DEST, Amp^R^	This study

### Estimation of the threshold *E*. *coli* cell density resistant to AvrRxo1 toxicity

Freshly streaked strains of *E*. *coli* transformed with IPTG inducible plasmid pDEST527 carrying wild type *6xHis-avrRxo1*, *6xHis-avrRxo1-T167N*, *6xHis-avrRxo1-D193T* and the pDESTcv plasmid were grown for 4 h. Exponentially growing cells were diluted to OD_600_ 0.2 and then 2-fold serial dilutions were spotted onto LB agar plates with and without the inducer 1 mM IPTG. Colonies were photographed after 24 h incubation at 37° C.

### Detection of *avrRxo1*-induced toxicity in *S*. *cerevisiae*

Freshly streaked strains of *S*. *cerevisiae* transformed with pESC-TRP-DEST vectors carrying wild type *avrRxo1*, *avrRxo1*-*D193T* or *avrRxo1*-*T167N* were grown overnight in media containing 2% glucose. Exponentially growing cultures were serially diluted and spotted on SD agar plates containing galactose/raffinose (2%/1%).

### Protein expression in *E*. *coli* and *S*. *cerevisiae*

Overnight cultures of *E*. *coli* transformed with pDEST527-derived plasmids were suspended to OD_600_ 0.2 and expression was induced by addition of 1 mM IPTG. At 6 hpi, cells were harvested and washed thrice with sterile ice cold HPLC grade water. Cell pellets were frozen in liquid nitrogen and stored at -80° C prior to analysis.

For protein expression in yeast, cultures of *Saccharomyces cerevisiae* transformed with pESC-TRP-DEST vectors carrying wild type *avrRxo1*, *avrRxo1*-*D193T* or pESC-TRP alone were grown in SD media containing 2% glucose for 3 days. Cultures were washed, resuspended in SD media containing 2% galactose and adjusted to OD_600_ 0.5. At 6 hpi in galactose-containing media, cells were harvested and washed thrice in sterile ice cold HPLC grade water. Cell pellets were snap-frozen and stored at -80° C until further use.

### Purification of polyhistidine-tagged proteins

Overnight cultures of *E*. *coli* transformed with pDEST527-AvrRxo1 and pDEST527-AvrRxo1-D193T were diluted 1:100 with LB medium and grown at 30° C. When the culture density reached OD_600_ 0.4, expression was induced by addition of 1 mM IPTG. After 5 h, cells were harvested, resuspended in TBS (Tris buffered Saline, pH 7.2), lysed by sonication and incubated in bacterial protein extraction reagent (ThermoFisher Scientific, Rockford, IL). for 30 min before centrifugation of pellet. Proteins were purified using HisPur Colbalt Resin (ThermoFisher Scientific, Rockford, IL) according to manufacturer’s instructions and eluted in TBS with 0.3 M imidazole. Samples were desalted using Zeba Spin Desalting columns, 7K MWCO (ThermoFisher Scientific, Rockford, IL) according to manufacturer’s instructions, and eluted in water. Purified proteins were stored at -80° C until further use.

### *In vitro* NAD kinase assay

The *in vitro* NAD kinase assay was performed according to Kornberg *et al*. [48], with modifications. Briefly, a reaction mixture comprising of purified 6xHis-AvrRxo1 or 6xHis-AvrRxo1-D193T (3 μM), 5 mM NAD^+^, 5 mM ATP, 10 mM MgCl_2_, 100 mM Tris-HCl (pH 7.5) was incubated for 1.5 h or overnight at 29° C. The samples were snap-frozen and stored at -80° C until further use.

### *A*. *tumefaciens*-mediated transient expression

*A*. *tumefaciens* strain GV2260 was used for transient expression in *N*. *benthamiana*. *A*. *tumefaciens* strains carrying different constructs were re-suspended in Agro-infiltration buffer (1M MgCl_2_, 1 M MES, and 200 μM acetosyringone) to OD_600_ 0.8. Leaves of 4-5-week old *N*. *benthamiana* were infiltrated using a needleless syringe on the abaxial surface and the plants were incubated in 16 h daylight. For LC-MS based metabolic profiling, leaf discs were collected 36 hpi, macerated in liquid nitrogen and stored at -80° C until further use. For recording of watersoaking, photographs were taken 50 hpi.

### Inoculation of rice leaves with *Xanthomonas oryzae* pv. *oryzicola*

Leaves of 6-week-old *Oryza sativa* ssp. *Japonica* cv. *Kitaake* were inoculated with cell suspensions of *X*. *oryzae* pv. *oryzicola* strains (OD_600_ 0.2) prepared in water from a 48- to 72-h-old PSA plate cultures. The inoculations were done by infiltration of the suspensions on the abaxial leaf surface using a needleless syringe. For LC-MS based metabolic profiling, leaf discs were collected 12, 24, 48 hpi, macerated in liquid nitrogen and stored at -80μ C until further use.

### Induction of Rxo1-mediated HR response in rice

Four-week-old transgenic rice plants (cv. Kitaake) expressing Rxo1 [6] were grown in a growth chamber, and fully-expanded leaves were inoculated with 10^8^ cfu/mL *X*. *oryzae* strain X11-5A carrying pHM1, pHM1-AvrRxo1, pHM1-AvrRxo1-T167N, or pHM1-AvrRxo1-D193T by leaf infiltration using needleless syringe as described by [6]. Inoculation sites were photographed at 5 days post infiltration.

### Metabolite extraction

For samples from *E*. *coli* and *S*. *cerevisiae* expressing *avrRxo1* or *avrRxo1-D193T*, 500 μL of MTBE solution (2:3 75% methanol: methyl tert-butyl ether v:v) was added to cell pellets, followed by 90 min of sonication at room temperature. 125 μL of H_2_O was added to induce phase separation, followed by 5 min of vortexing. After centrifugation at 2095 × g for 15 min at 4° C, the bottom aqueous phase was removed and analyzed by LC-MS.

For *in vitro* kinase assay reaction mixtures and infiltrated *N*. *benthamiana* leaf disc samples, 500 μL MTBE solution was added followed by 30 min of sonication, 30 min vortexing, and again 30 min of sonication at room temperature. 300 μL of H_2_O was added to induce phase separation followed by 15 min vortexing. After centrifugation at 2095 × g for 15 min at 4° C, the bottom, aqueous phase was removed, dried down under a gentle stream of nitrogen, resuspended in 150 μL of 40% methanol and then analyzed by LC-MS.

For inoculated rice leaf samples, 1000 μL MTBE solution was added to each sample followed by 90 min of sonication at room temperature. 250 μL of H_2_O was added to induce phase separation followed by 5 min of vortexing. After centrifugation at 2095 × g for 15 min at 4° C, the bottom aqueous phase was removed and analyzed by LC-MS.

### LC-MS

Liquid chromatography coupled to mass spectrometry (LC-MS) was performed on a Waters Acquity UPLC system coupled to a Waters Xevo G2 time-of-flight (TOF) mass spectrometer (MS) for untargeted metabolomics analysis. For high resolution ms/ms experiments, a Waters Q-TOF MS was used to selectively fragment the parent ion of interest. A ZIC-pHILIC stationary phase (Merck Millipore, 150 x 2.1 mm, 5 μM) was used for the separation of polar metabolites. Mobile phase A was H_2_O with 10 mM NH_4_HCO_3_ adjusted to pH 9.6 with NH_4_OH and mobile phase B was acetonitrile. Flow rate was 270 μL/min and the column was held at 50° C. Injection volume was 2 μL. The gradient was as follows: 0 min 90% B, 1.5 min 90% B, 8.5 min 62% B, 11 min 40% B. The column was washed at 150 μL/min with 5% B for 1 min, then equilibrated at starting conditions with 7.3 column volumes of solvent.

For experiments performed on the TOF-MS, source and desolvation temperatures were at 150° C and 350° C, respectively. Desolvation gas flow was 850 L/hr. The MS was operated in full scan mode with an m/z range of 50–1200. Capillary voltage in negative ion mode was 2.2 kV.

For experiments performed on the QQQ-MS, source and desolvation temperatures were at 150° C and 500° C respectively. Desolvation, cone, collision, and nebuliser gas flows were 1000 L/hr, 150 L/hr, 0.2 mL/min, and 7 Bar, respectively. The MS was operated in selected reaction monitoring mode. 2’- and 3’-NADP have a precursor ion of 744.08 and product ions of 604 (collision energy = 20) and 136 (CE = 35). Capillary voltage in positive ion mode was 3.1 kV.

### Detection of flg22-triggered oxidative burst in *N*. *benthamiana*

Detection of the flg22-based oxidative burst was performed using a luminol-HRP-based chemiluminescence assay. 24 h after infiltration of *N*. *benthamiana* leaves with *A*. *tumefaciens*, leaf discs (5 mm diameter) were collected and incubated overnight in H_2_O. Per measurement, two leaf discs were removed from H_2_O and transferred into cuvette containing 300 μL assay solution (20 mM luminol, 1 μg/ml horseradish peroxidase and 100 nM flg22 (EZBiolab, Carmel, IN, USA) or H_2_O (mock)). Luminescence was measured over a period of 30–40 min using an FB12 single tube Luminometer (Berthold Detection Systems GmbH, Pforzheim, Germany).

### Measurement of menadione generated intracellular oxidation levels in *Saccharomyces cerevisiae*

The oxidant-sensitive probe H_2_DCFDA (ThermoFisher Scientific) was used to determine the intracellular levels of ROS generated in yeast cells after treatment with menadione. Yeast cells were grown overnight in media containing glucose. They were then washed and diluted in SD media containing galactose/ raffinose for induction of expression. Exponentially growing cells were harvested at *OD*_600_ 0.8, washed and resuspended in 10 mm potassium buffer (6.15 mM K_2_HPO_4_, 3.85 mM KH_2_PO_4_, pH 7.0) followed by incubation for 30 min in the same buffer with 10 μM of H_2_DCFDA. After 60 min exposure to 8 mM menadione, cells were washed and resuspended in distilled water and disrupted using glass beads. Fluorescence of the cell lysate was measured at λ_EX_ = 490 nm and λ_EM_ = 519 nm using Synergy HI microplate reader (Biotek). The values were further normalized by protein concentration.

### Production of anti-AvrRxo1 polyclonal antibody

A DNA fragment containing AvrRxo1 (65-421aa) was amplified from the genomic DNA of *Xanthomonas oryzae* pv. *oryzicola* strain BLS256, and cloned into the protein expression vector pGEX4T-1 (GE Healthcare Bio-Sciences, Pittsburgh, PA). To facilitate the removal of GST tag, a TEV protease cleavage site was inserted between the GST and targeted protein. The GST-AvrRxo1 (65-421aa) fusion protein was expressed in *E*. *coli* and purified as previously described [8]. The GST tag was removed by cleavage with AcTEV (ThermoFisher Scientific, Waltham, MA) and separated on PAGE. The protein band corresponding to AvrRxo1 (65-421aa) was used to generate anti-rabbit polyclonal antibodies (Cocalico Biologicals, Inc., Reamstown, PA).

### Immunoblot analysis

To confirm expression of AvrRxo1 and derivatives in X11-5A, leaves of six-week-old *O*. *sativa* cv. *Kitaake* plants were inoculated with cell suspensions of *X*. *oryzae* pv. *oryzicola* strains as described above. Inoculated 3-cm leaf sections were collected at 48h, homogenized in liquid nitrogen, and then ground in 100 uL crude extraction buffer (50 mM Tris-HCl, pH 7.5, 2% SDS, 10 mM DTT, and 1 mM PMSF). Lysates were mixed 4:1 with urea-amended SDS loading dye, for a final concentration of 1 M urea. Roughly 50 μg of total protein sample was separated on 10% SDS/PAGE gel and transferred to a PVDF membrane or stained with GelCode Blue Stain Reagent (ThermoFisher Scientific, Rockford, IL). Immunodetection was performed in a 1:3000 dilution anti-AvrRxo1 polyclonal antibody, followed by a 1:5000 dilution of goat anti-rabbit IgG-HRP conjugate. Commercial antibodies were obtained from ThermoFisher Scientific (Rockford, IL). The blot was incubated with ECL Plus Western blotting detection reagents (GE Healthcare, Amersham) following the manufacturer's instructions, and blot was developed for two minutes on autoradiography film.

To confirm expression of AvrRxo1 in transient expression experiments, *N*. *benthamiana* leaf disks were collected at 42 hours after infiltration with *Agrobacterium* strains for transient expression of HA-AvrRxo1, HA-AvrRxo1(D193T) and HA-AvrRxo1(T167N); this is after initial appearance of watersoaking in the HA-AvrRxo1 treatment but prior to total leaf tissue collapse. Lysis and separation was performed using the protocol above. Immunodetection was performed in a 1:1000 dilution mouse anti-HA primary antibody followed by a 1:3000 dilution of goat anti-mouse IgG-HRP conjugate. Blot developing was performed as described above except that the blot was visualized under a Chemi-Doc-It 2 chemiluminescence imager (UVP, Upland, CA).

## Supporting information

S1 Fig Analysis of catalytic site derivative expression in *N*. *benthamiana* post transient expression. (A) Western blot analysis of HA-AvrRxo1 and catalytic site mutants HA-AvrRxo1-D193T and HA-AvrRxo1-T167N after transient expression in *N*. *benthamiana*. Leaf samples were collected 50 hpi, the time point used in Fig 1C; no expression of any treatment was detected in samples collected at 36 h, the time point used for detection in Fig 3. Blot was imaged using a luminescence-detecting camera. (B) In-gel detection of Rubisco observed from the same protein extracts using Coomassie staining.
(EPS)

S2 Fig Analysis of catalytic site derivative expression in rice tissues after inoculation with AvrRxo1-expressing derivatives of *Xoo* strain X11-5A. (A) Western blot analysis of AvrRxo1 in tissues of rice cv. Kitaake after inoculation with X11-5A (pHM1-AvrRxo1), X11-5A (pHM1-AvrRxo1-D193T), X11-5A (pHM1-AvrRxo1-T167N), and X11-5A (pHM1). (B) In-gel detection of Rubisco observed from the same protein extracts using Coomassie staining.
(EPS)

S3 Fig Identification of metabolic changes in *E*. *coli* cultures expressing AvrRxo1. Hierarchical clustering analysis summarizing relative changes in abundance of 79 annotated metabolites obtained upon LC-MS analysis of IPTG-induced *E*. *coli* carrying pDESTcv, pDEST-AvrRxo1 or pDEST-AvrRxo1-D193T (n = 3).
(EPS)

S4 Fig Identification of metabolic changes in *S*. *cerevisiae* cultures expressing AvrRxo1. Hierarchical clustering analysis summarizing relative changes in abundance of 79 annotated metabolites obtained upon LC-MS analysis of *S*. *cerevisiae* cultures carrying the vector pESC-TRP, pESC-TRP-DEST-AvrRxo1 (WT) or pESC-TRP-DEST-AvrRxo1-D193T in inducing media (n = 3, n = 2 for WT)
(EPS)

S5 Fig Identification of the AvrRxo1-dependent compound. (A-B) TOF-MS/MS-generated spectra of NADP from lysates of *E*. *coli* transformed with pDESTcv (A) or expressing 6xHis-AvrRxo1 spiked with NADP (B). (C-D) Mass spectra from the AvrRxo1-dependent peak (C) was a spectral match to that of NADP (D).
(EPS)

S6 Fig Confirmation of identity of the AvrRxo1-dependent compound. (A-B) Mass spectra of the suspected 3’-NADP signal in AvrRxo1-expressing bacteria (A) and that of a synthetic 3’-NADP standard (B).
(EPS)

S7 Fig Purified 6xHis-AvrRxo1, but not AvrRxo1-D193T, phosphorylates NAD *in vitro*. (A) Eluate of 6xHis-AvrRxo1 and AvrRxo1-D193T purification. The middle three lanes show a co-purification with Arc1, not relevant to this study. (B-D) LC-MS/MS analysis of NADP in a neat solution containing synthetic NADP and 3’-NADP standards (overlaid chromatograms) (B), and *in vitro* kinase assays using purified 6xHis-AvrRxo1 (C) or 6xHis-AvrRxoI-D193T (D) incubated overnight in buffer with ATP and NAD.
(EPS)

S8 Fig 3’-NADP can be detected in rice leaves after inoculation with MAI10 (pHM1-AvrRxo1) but not with MAI10 or MAI10 (pHM1). (A) LC-MS/MS analysis of NADP species from rice leaves 48h after inoculation with the *avrRxo1-*negative strain *X*. *oryzae* pv. *oryzicola* MAI10, MAI10 (pHM1), and MAI10 (pHM1-AvrRxo1). (B) Relative peak areas at the position of the 3’-NADP commercial standard for four replicate samples inoculated with MAI10, MAI10 (pHM1), and MAI10 (pHM1-AvrRxo1).
(EPS)

S9 Fig Rice leaves inoculated with an AvrRxo1- producing *Xoc* strain accumulate 3’-NADP within 12h. (A-F) LC-MS/MS analysis of NADP species from rice leaves 12 and 24 h after infiltration with water (A, B), the *avrRxo1-*negative strain *X*. *oryzae* pv. *oryzicola* MAI10 (C, D), and the *avrRxo1-*positive strain BLS256 (E, F).
(EPS)

S1 File Normalized abundance of 79 annotated metabolites in bacteria, yeast, and rice metabolomic studies. Boxplots representing the range and standard deviation of the normalized abundance of 79 metabolites upon expression of AvrRxo1, AvrRxo1-D193T, or control in *E*. *coli* (ecoli) and *S*. *cerevisiae* (yeast), and 48h after infection of rice cv. Kitaake with *X*. *oryzae* strain X11-5A expressing *avrRxo1* or carrying an empty vector (rice). “BV” denotes pDESTcv in *E*. *coli*, pESC-TRP in yeast, and pHM1 in rice. “WT” denotes the AvrRxo1 treatment, and the D193T treatment is here labeled as “D143T” (n = 3 except for the WT treatment in *S*. *cerevisiae*, for which n = 2). Anova p-values by species and treatment are shown at the top left, with significant values highlighted in red.
(PDF)
