# Supplementary material for: The effector AvrRxo1 phosphorylates NAD in planta
Source: PLoS Pathog. 2017 Jun 19;13(6):e1006442. doi: 10.1371/journal.ppat.1006442 (PMC5491322; doi:10.1371/journal.ppat.1006442)

Sucrose

rt=481
C2: MSI conf = 1
notes:

	p−value
spp	<1e−05
trt	0.955
spp:trt	0.982

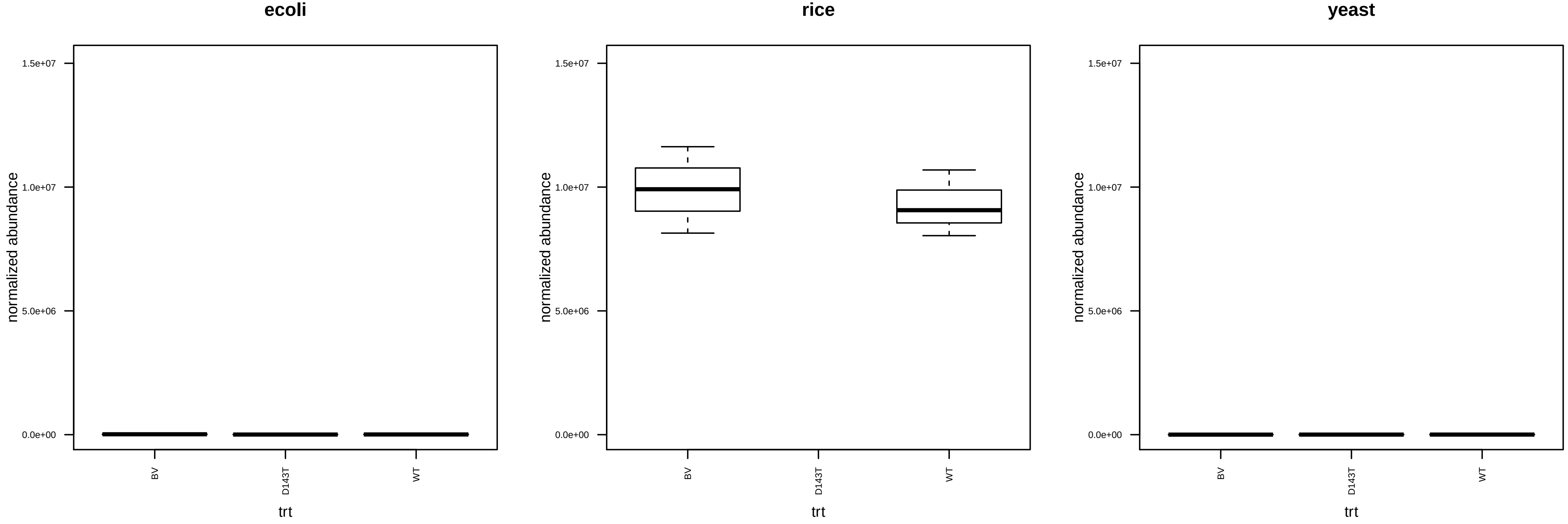

Rhamnocitrin 3−rhamninoside

rt=89
C3: MSI conf = 4
notes: Metlin:50992

	p−value
spp	<1e−05
trt	0.861
spp:trt	0.937

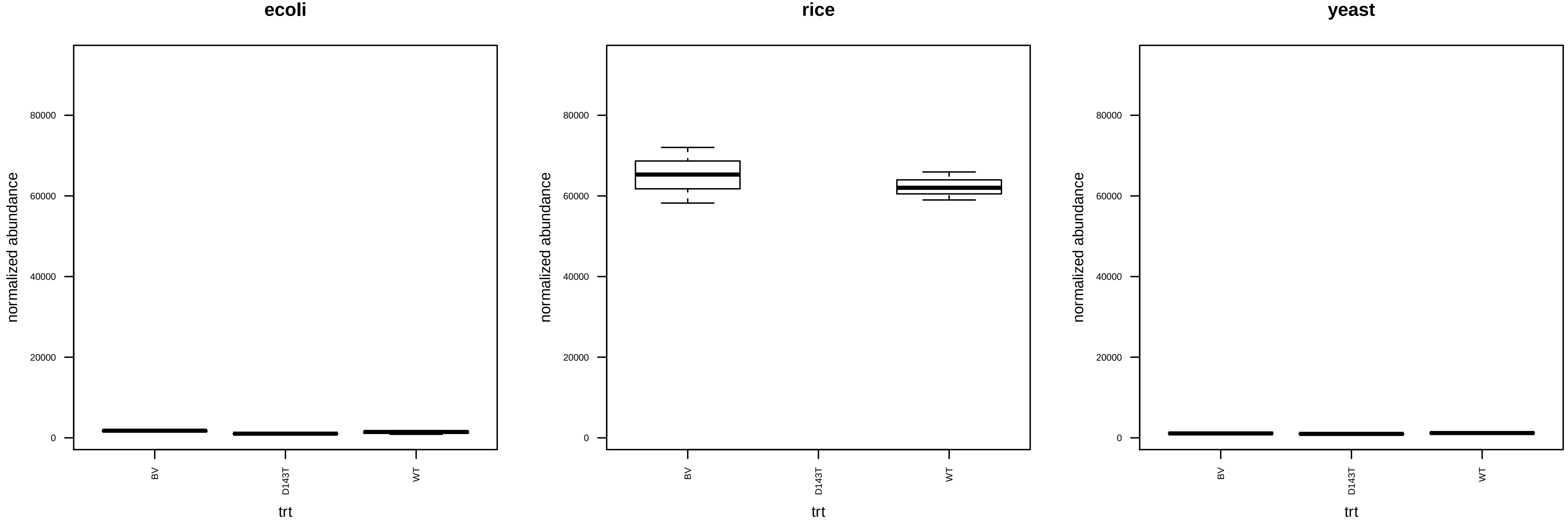

L−glutathione reduced

rt=424
C4: MSI conf = 1
notes:

	p−value
spp	< 1e−05
trt	0.000641
spp:trt	5.09e−05

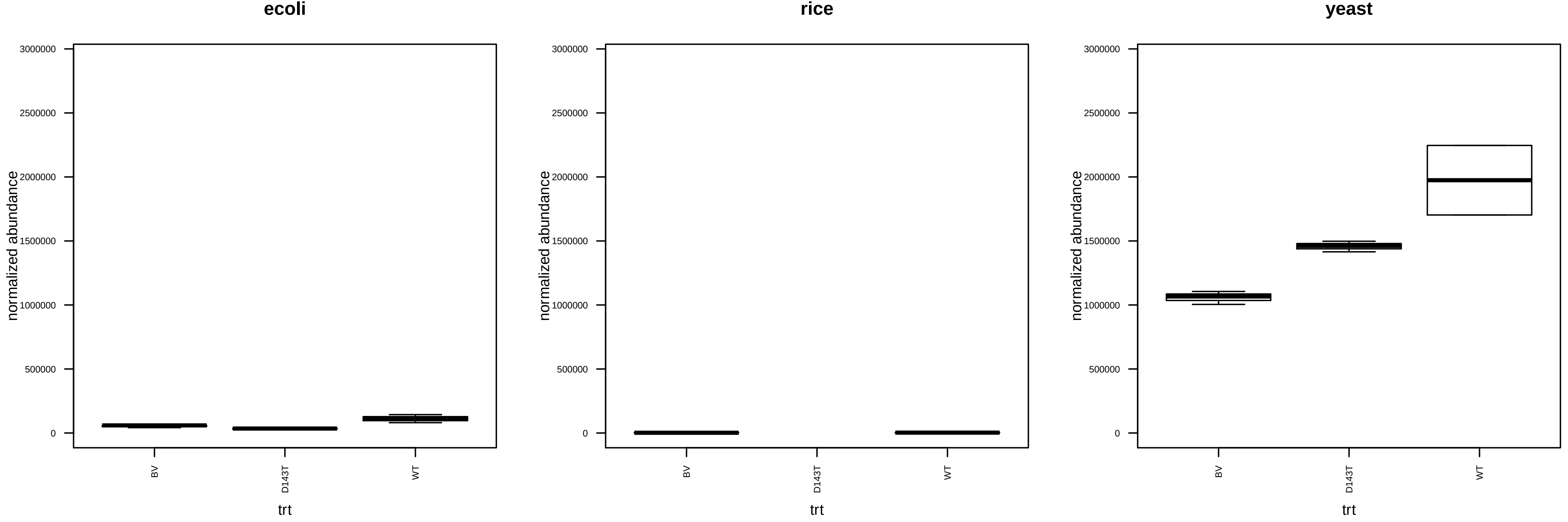

lysoPI(16:0)

rt=52
C5: MSI conf = 4
notes:

	p−value
spp	<1e−05
trt	0.978
spp:trt	1.000

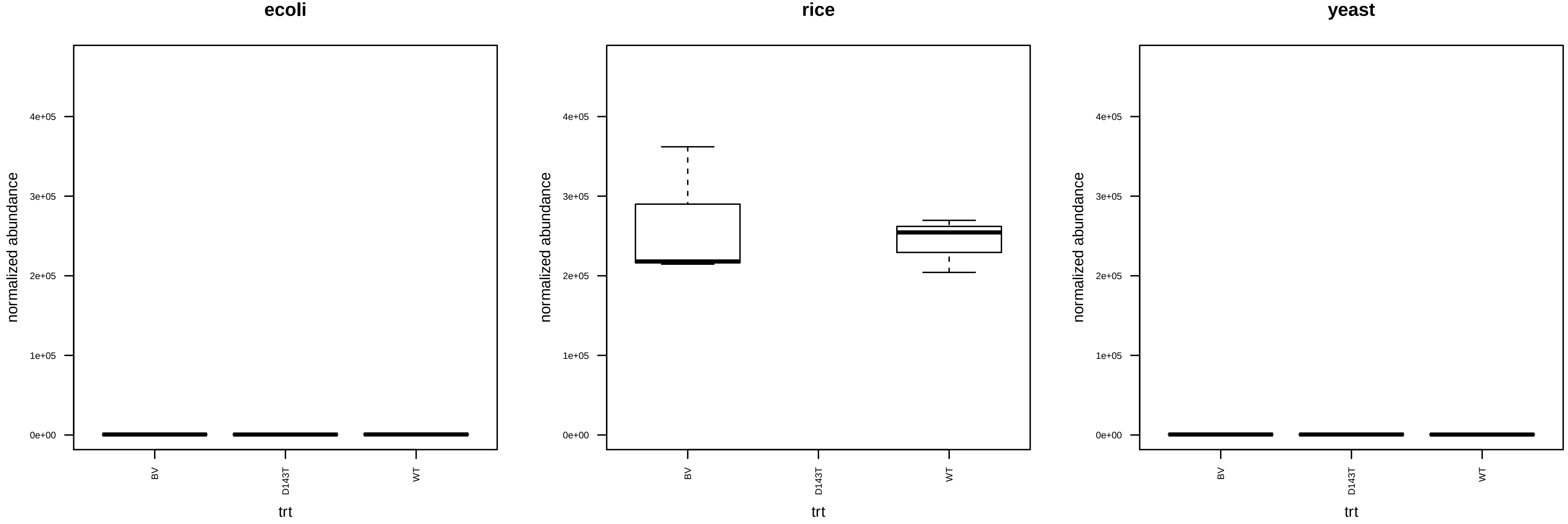

Isorhamnetin 3−(6'''−p−coumaroylglucosyl)−(1−>2)−rhamnoside

rt=285
C6: MSI conf = 4
notes: Metlin:50801

	p−value
spp	<1e−05
trt	0.840
spp:trt	0.857

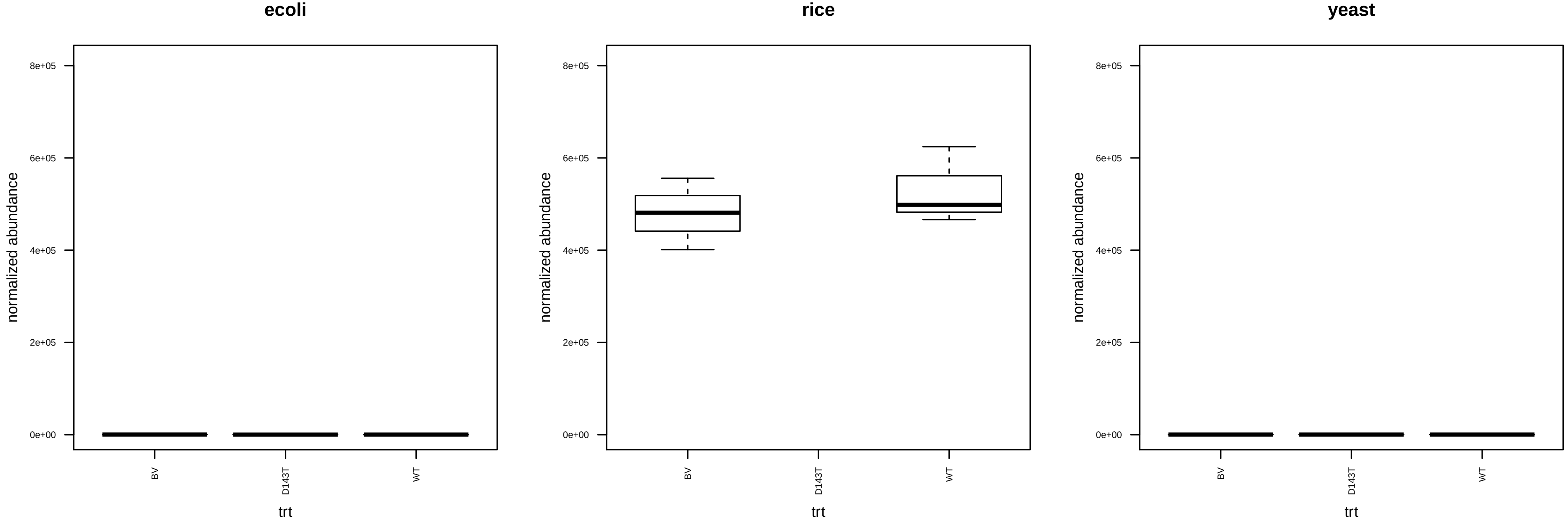

Flavin Mononucleotide

rt=93
C7: MSI conf = 1
notes:

	p−value
spp	<1e−05
trt	0.999
spp:trt	1.000

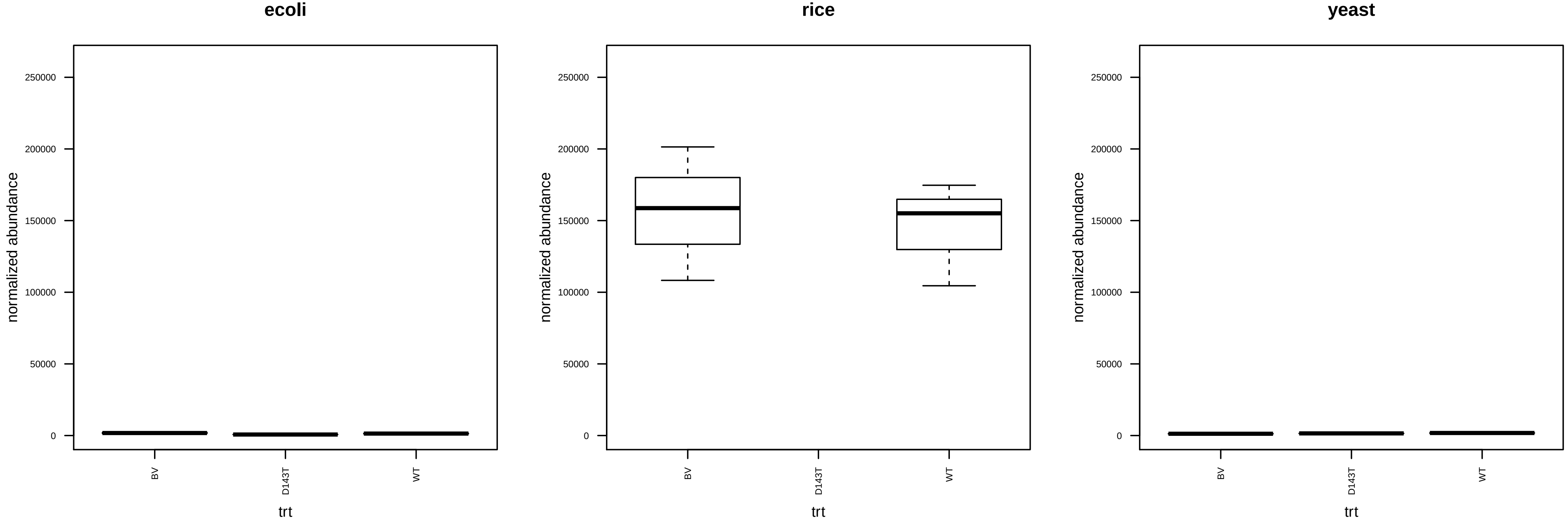

3,7−Dihydroxy−12−oxocholanoic acid−like

rt=91
C10: MSI conf = 3
notes:

	p−value
spp	<1e−05
trt	1
spp:trt	1

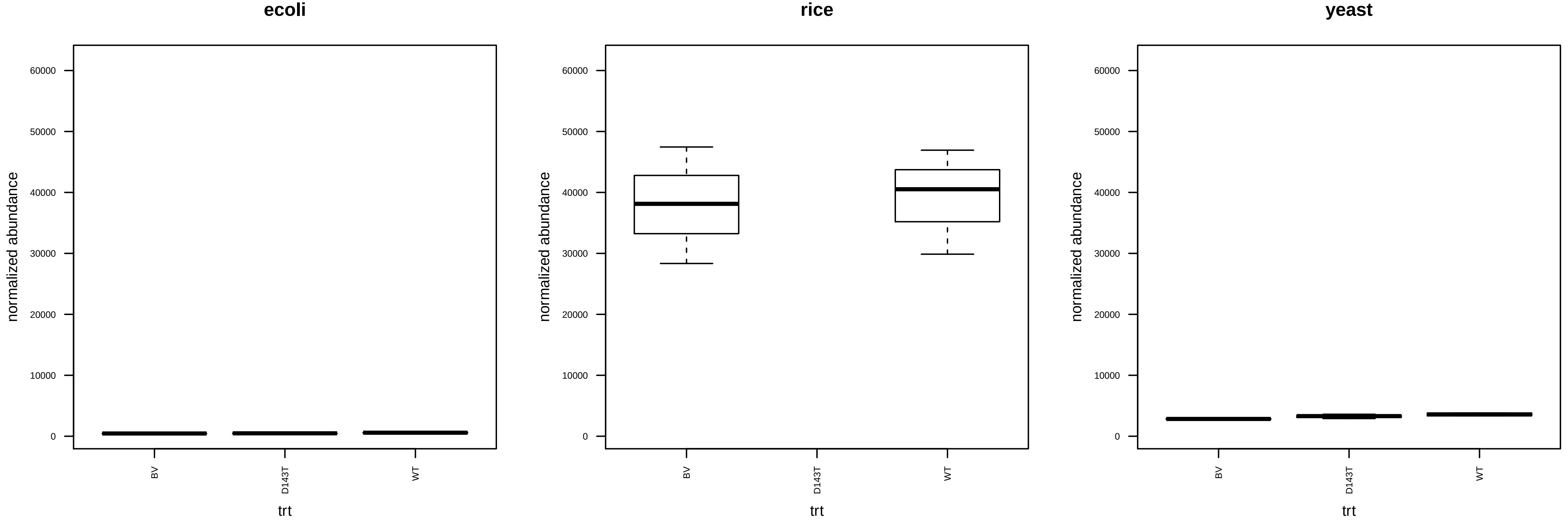

Uridine diphosphate−N−acetylglucosamine

rt=564
C17: MSI conf = 1
notes:

	p−value
spp	<1e−05
trt	0.128
spp:trt	0.134

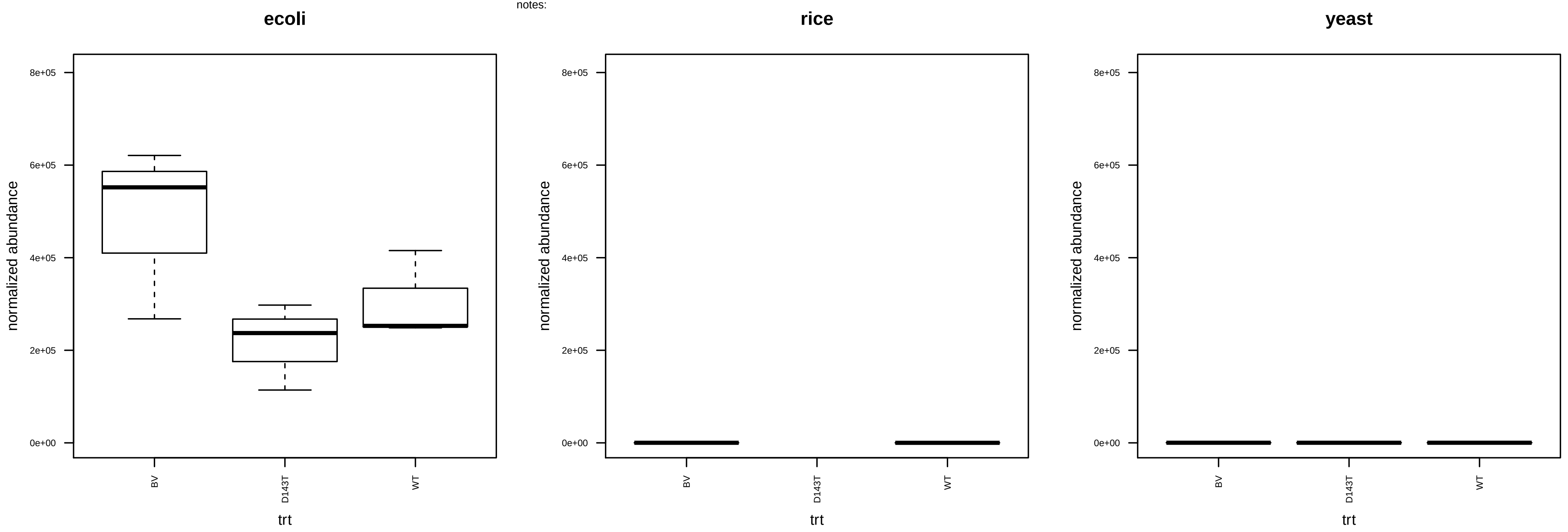

L−glutathione oxidized

rt=566
C18: MSI conf = 1
notes:

	p−value
spp	< 1e−05
trt	0.000246
spp:trt	0.009935

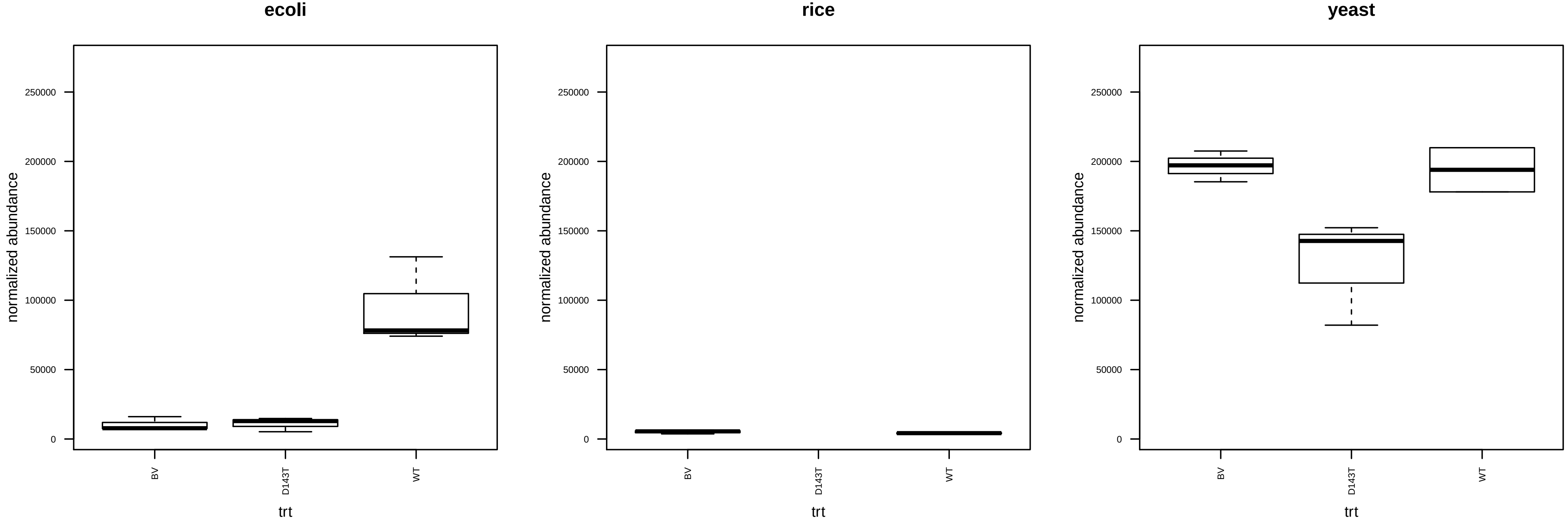

Salicylic acid .beta.−D−O−glucuronide

rt=53
C27: MSI conf = 2
notes:

	p−value
spp	<1e−05
trt	0.911
spp:trt	0.940

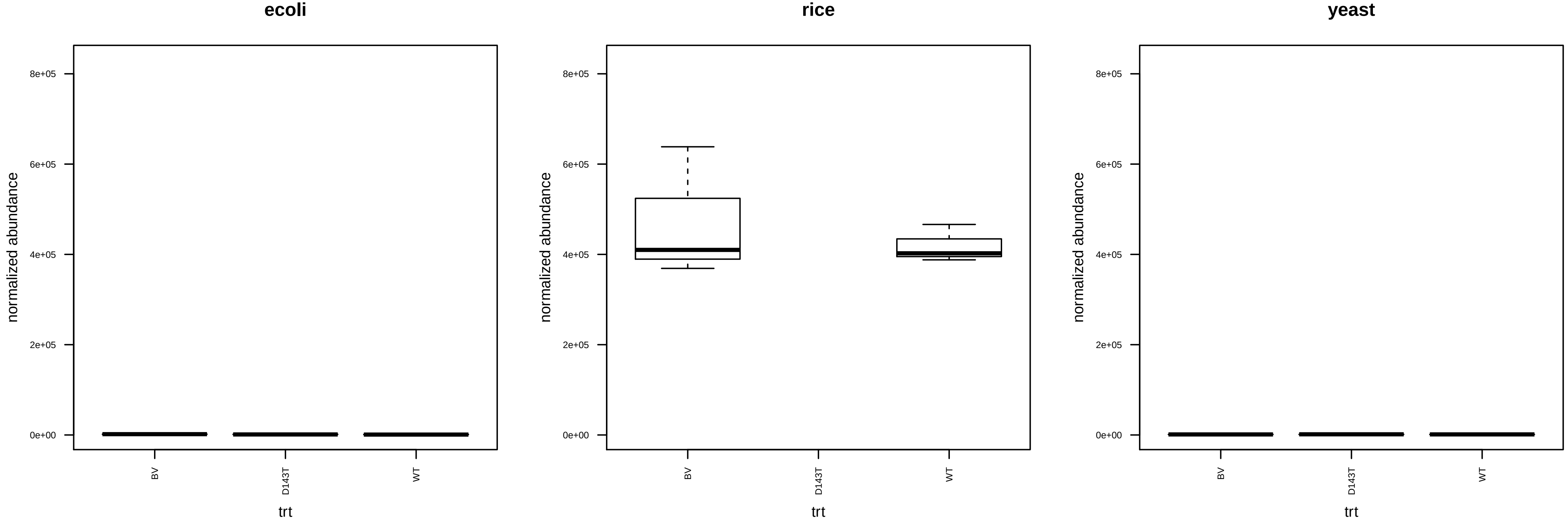

NADP

rt=544

C37: MSI conf = 2

notes: hybrid spectrum − 2' and 3' NADP, with 3' derived from AvrRxo

	p−value
spp	<1e−05
trt	<1e−05
spp:trt	<1e−05

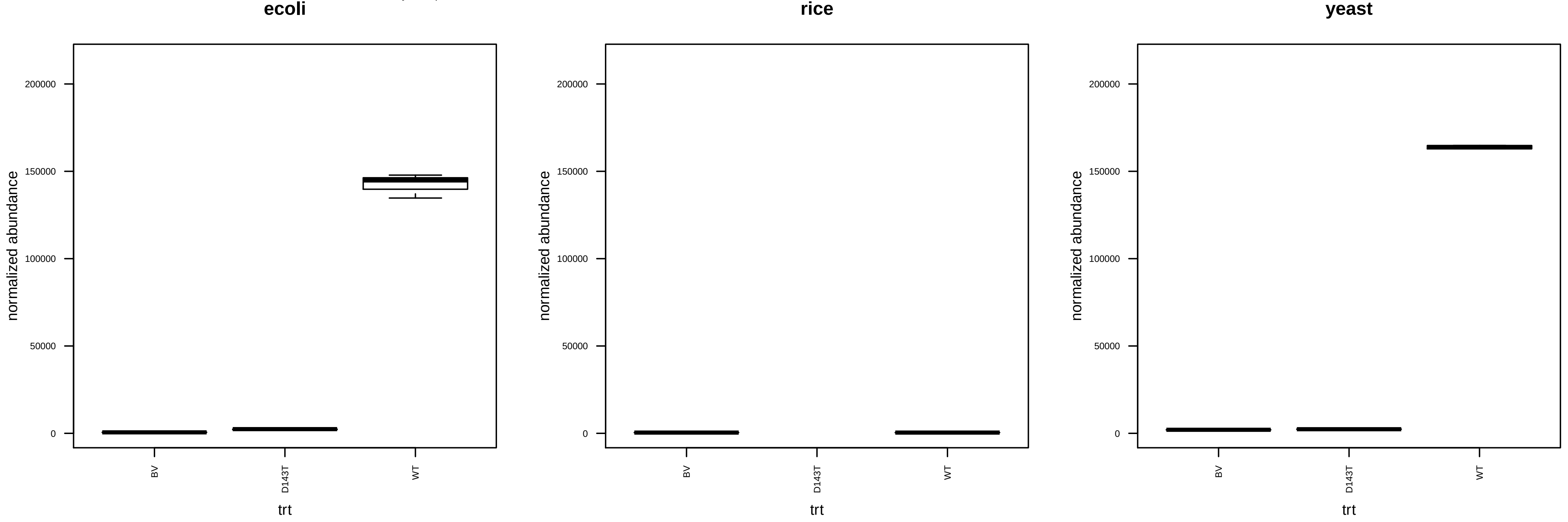

NAD

rt=452
C58: MSI conf = 2
notes:

	p−value
spp	< 1e−05
trt	0.0404
spp:trt	4.84e−05

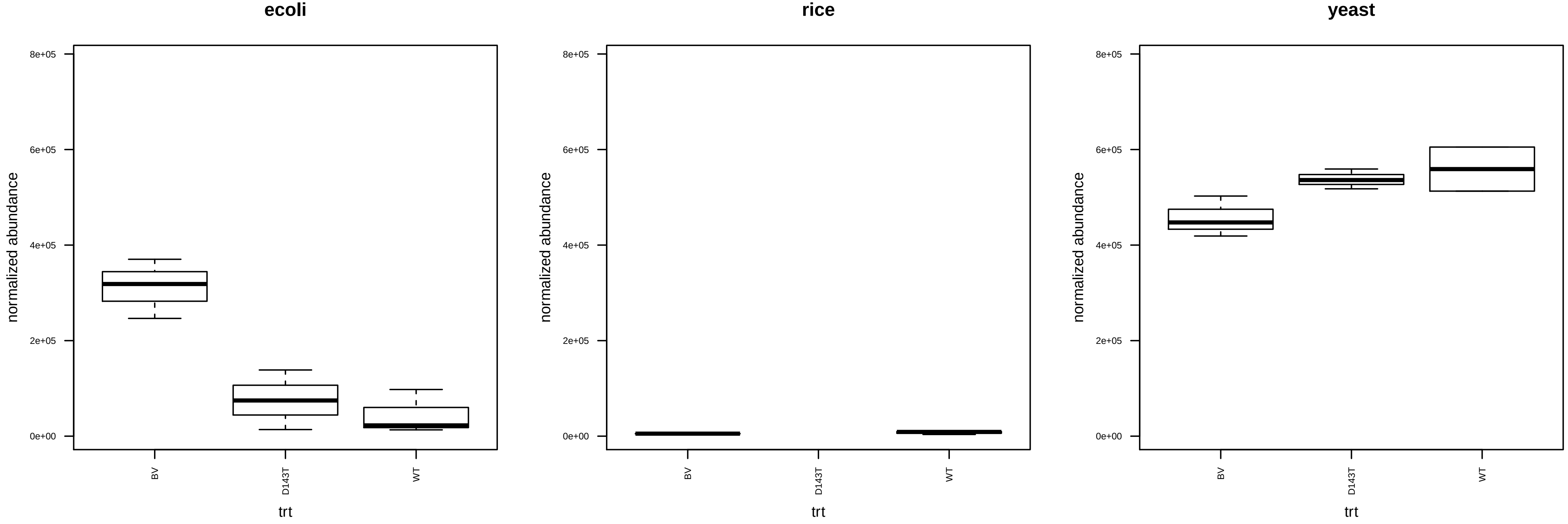

Glucose−6−phosphate

rt=531
C59: MSI conf = 1
notes:

	p−value
spp	< 1e−05
trt	0.000237
spp:trt	2.01e−05

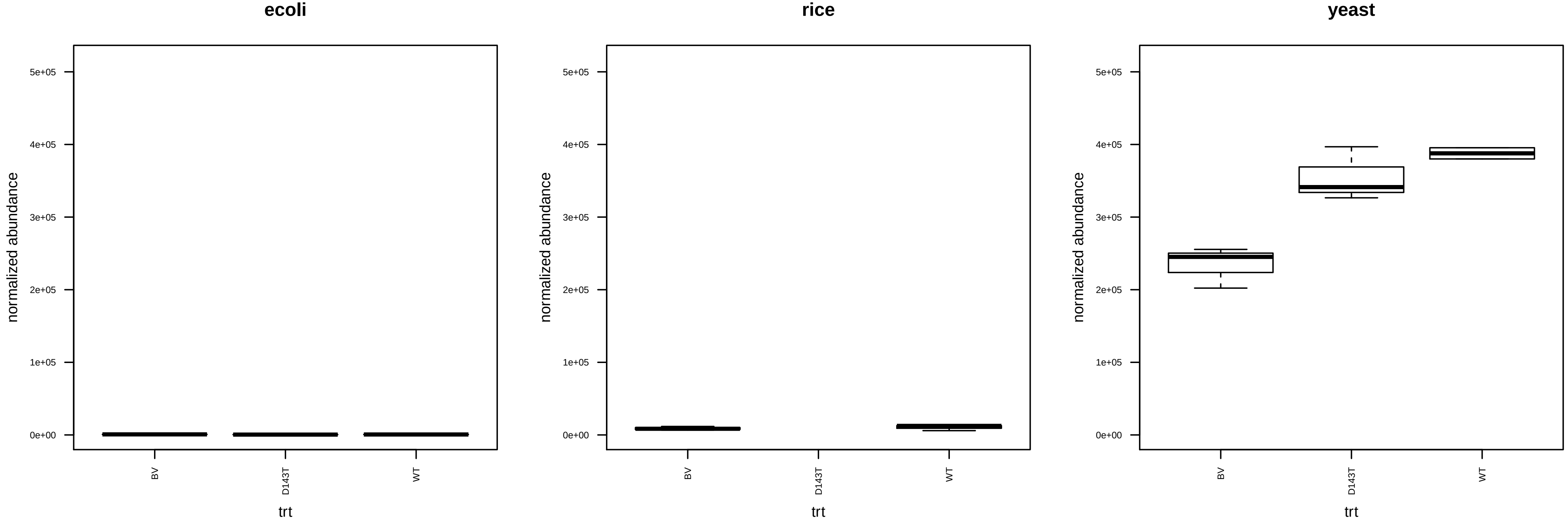

UDP−N−acetylmuramoyl−L−alanyl−D−glutamate

rt=583
C60: MSI conf = 4
notes: Metlin:63457

	p−value
spp	<1e−05
trt	<1e−05
spp:trt	<1e−05

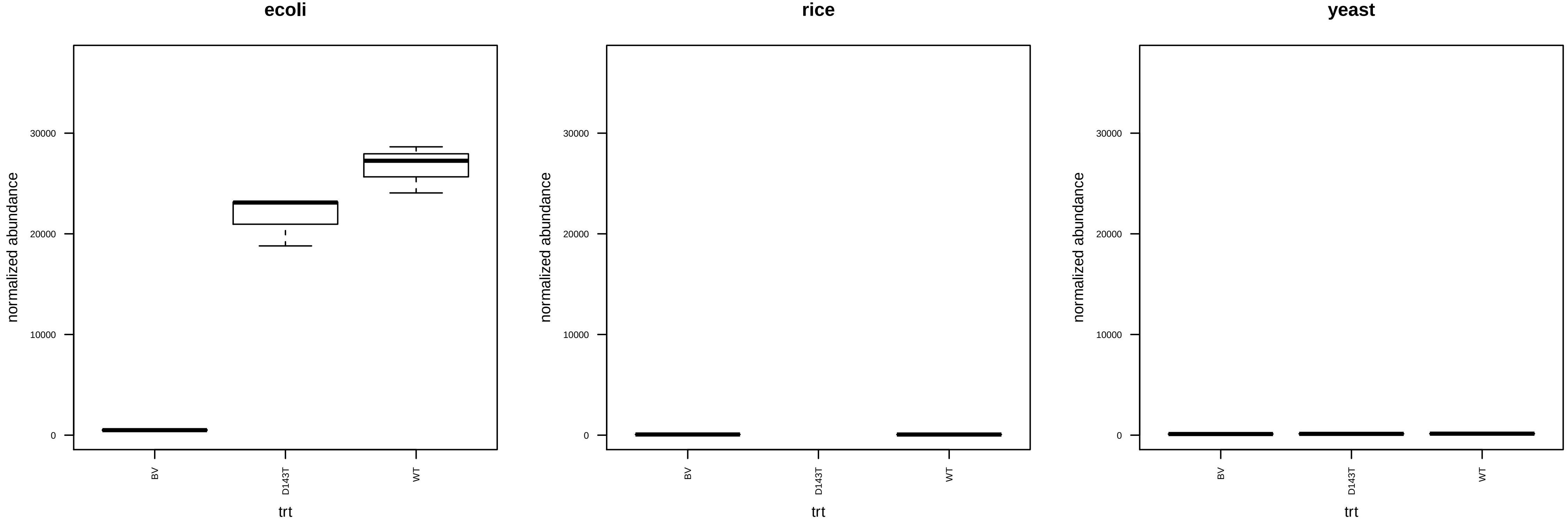

Uridine diphosphate−N−acetylglucosamine

rt=461
C72: MSI conf = 1
notes:

	p−value
spp	< 1e−05
trt	0.03022
spp:trt	0.00176

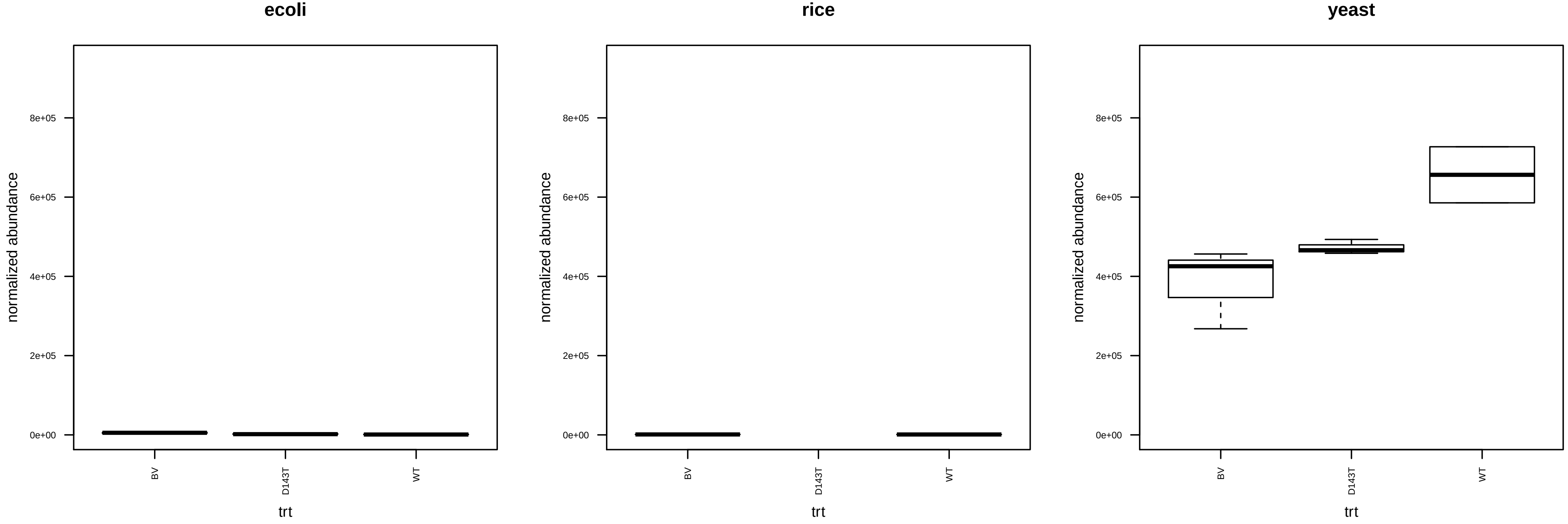

PA(32:0)

rt=47
C75: MSI conf = 1
notes:

	p−value
spp	< 1e−05
trt	0.000481
spp:trt	0.001507

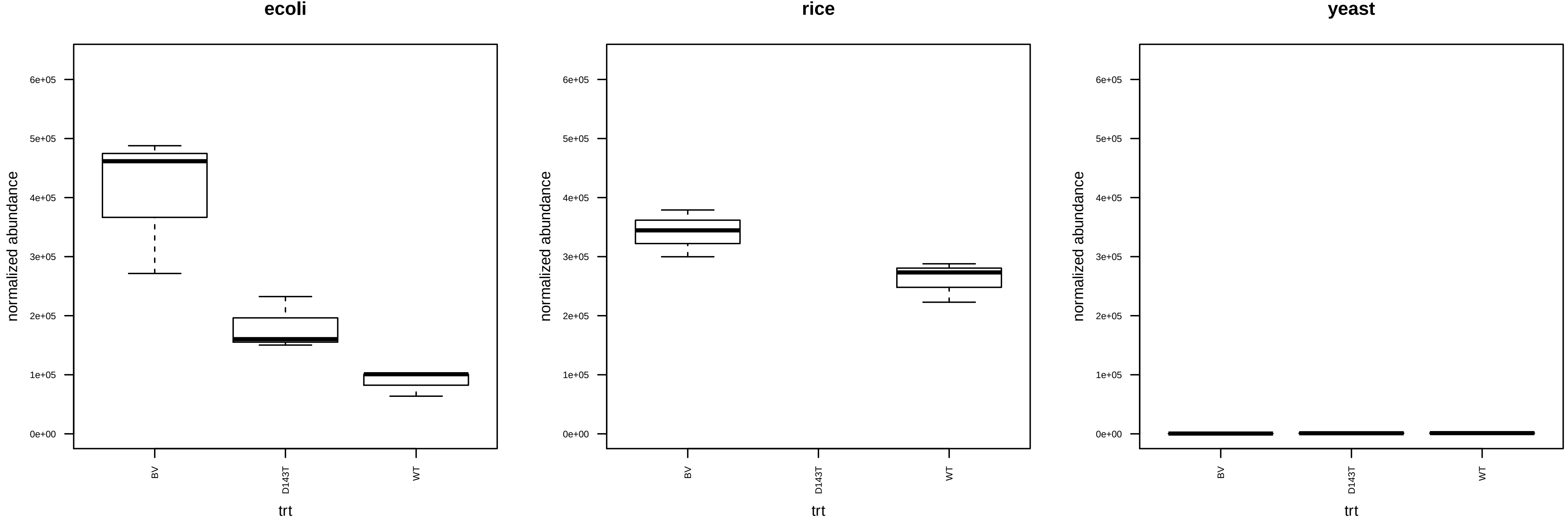

Adenosine triphosphate (ATP)

rt=556
C77: MSI conf = 1
notes:

	p−value
spp	<1e−05
trt	<1e−05
spp:trt	<1e−05

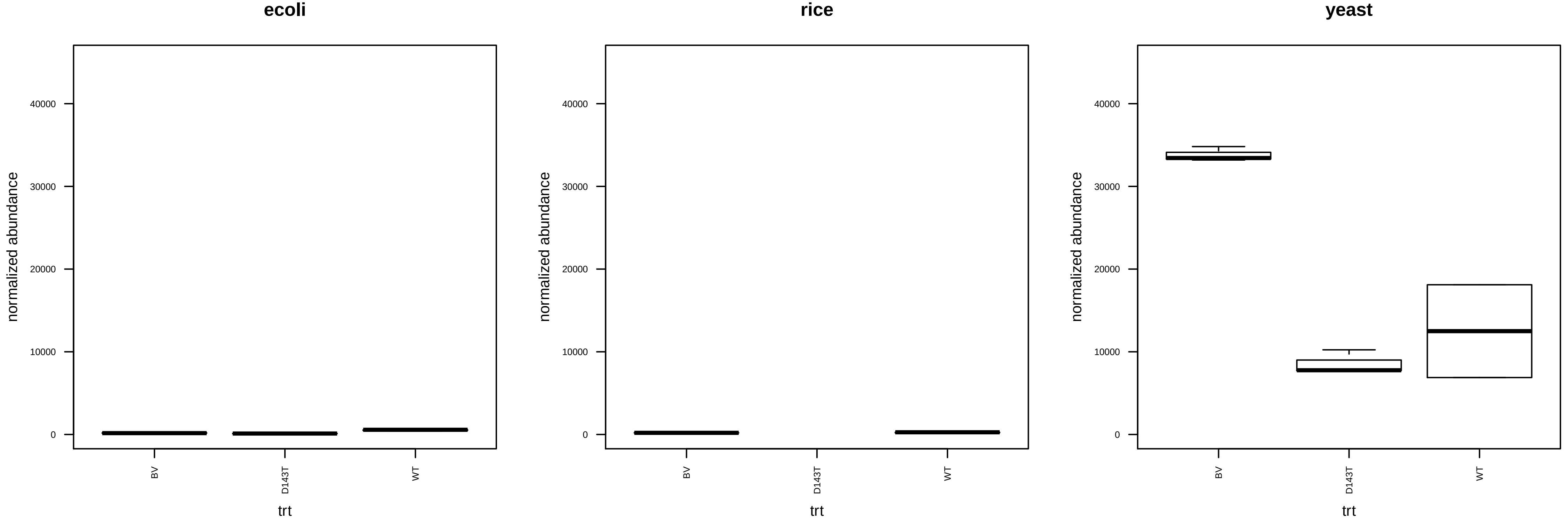

Citric acid

rt=566
C78: MSI conf = 1
notes:

	p−value
spp	2.46e−05
trt	0.652
spp:trt	0.810

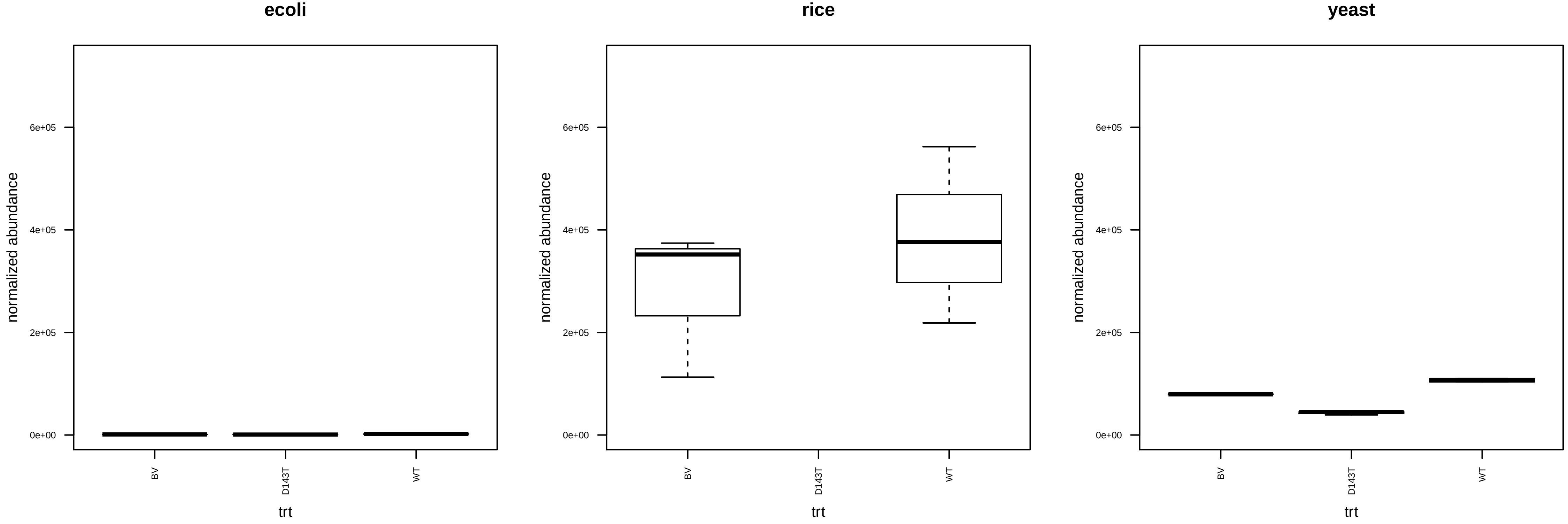

Malic acid

rt=434
C138: MSI conf = 1
notes:

	p−value
spp	<1e−05
trt	1
spp:trt	1

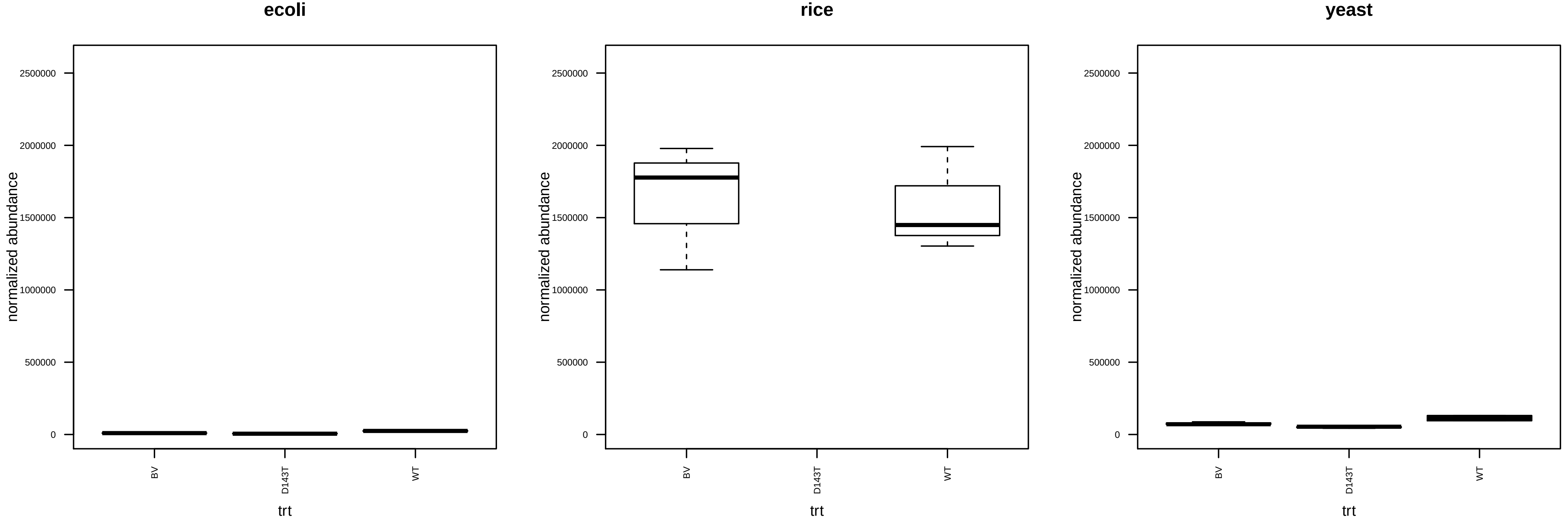

Quinic acid

rt=247
C141: MSI conf = 1
notes:

	p−value
spp	0.0115
trt	1.0000
spp:trt	1.0000

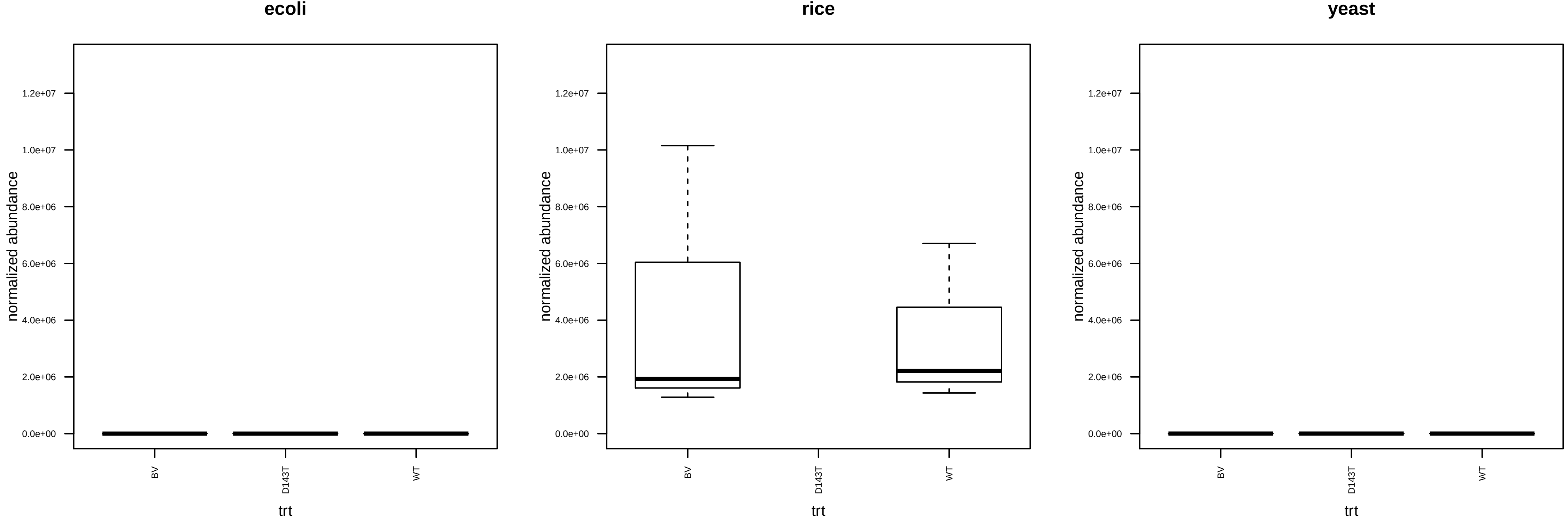

Glutathione

rt=417
C159: MSI conf = 1
notes:

	p−value
spp	< 1e−05
trt	2.93e−05
spp:trt	0.000786

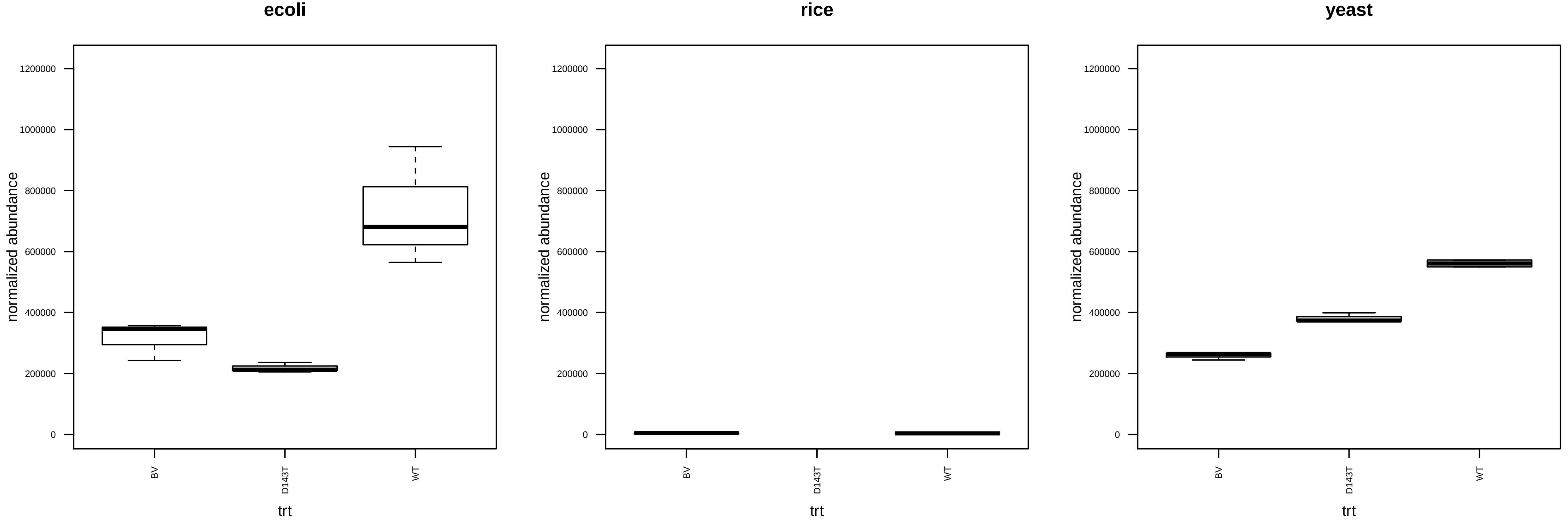

Quinic acid

rt=241
C164: MSI conf = 1
notes:

	p-value
spp	<1e−05
trt	0.0453
spp:trt	0.0155

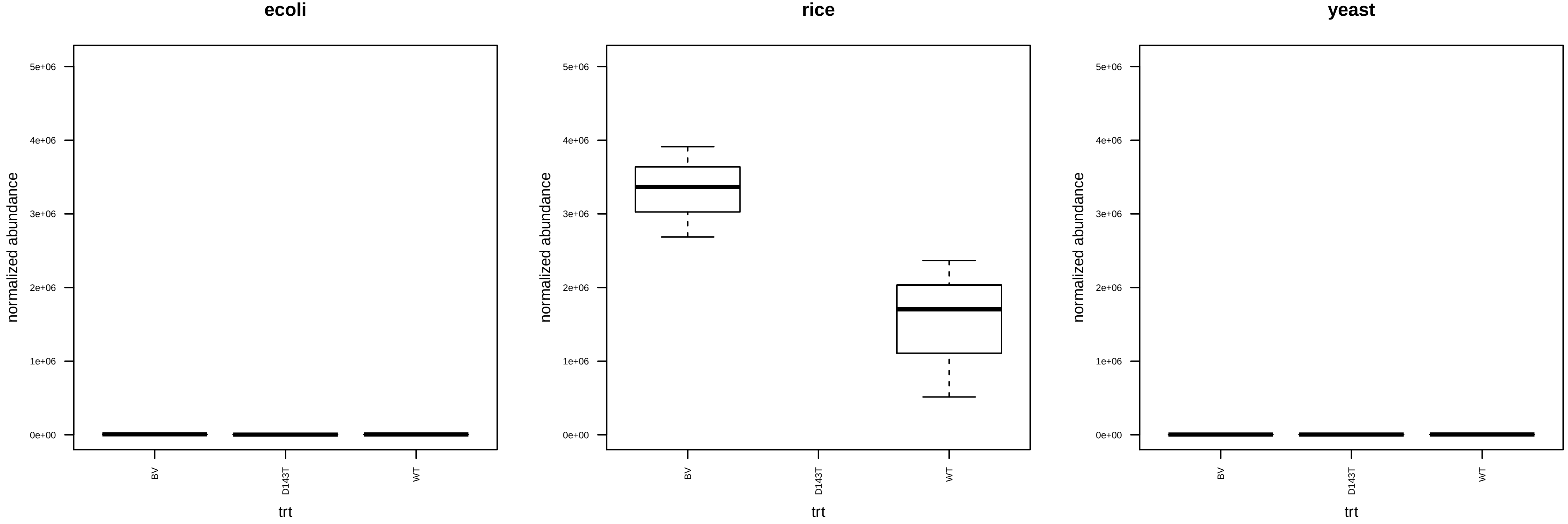

L−Tryptophan

rt=361
C184: MSI conf = 1
notes:

	p−value
spp	<1e−05
trt	<1e−05
spp:trt	<1e−05

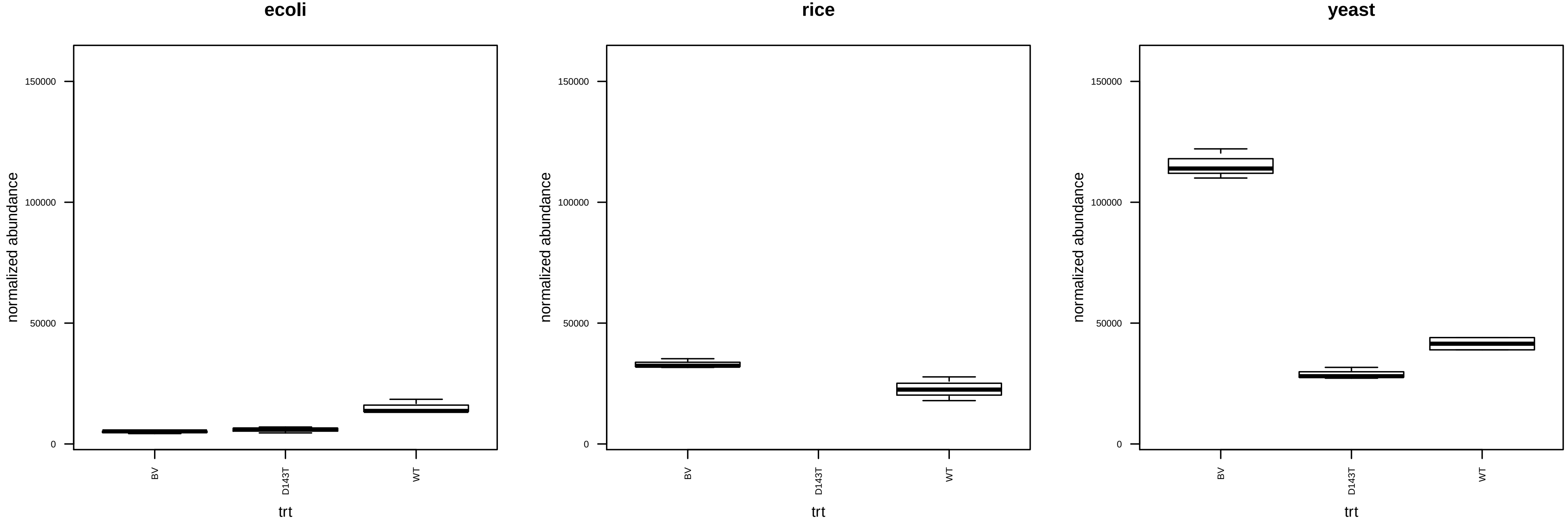

L−Phenylalanine

rt=291
C187: MSI conf = 1
notes:

	p−value
spp	< 1e−05
trt	0.000282
spp:trt	0.057193

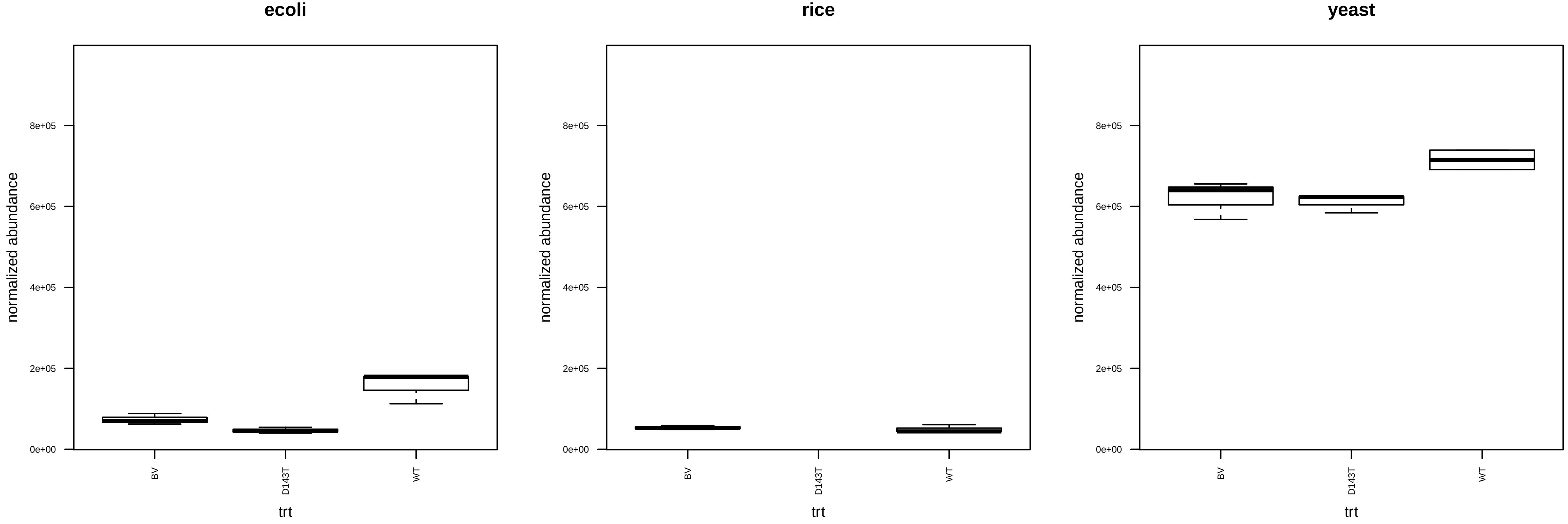

Diadenosine triphosphate

rt=502
C220: MSI conf = 4
notes:

	p−value
spp	<1e−05
trt	<1e−05
spp:trt	<1e−05

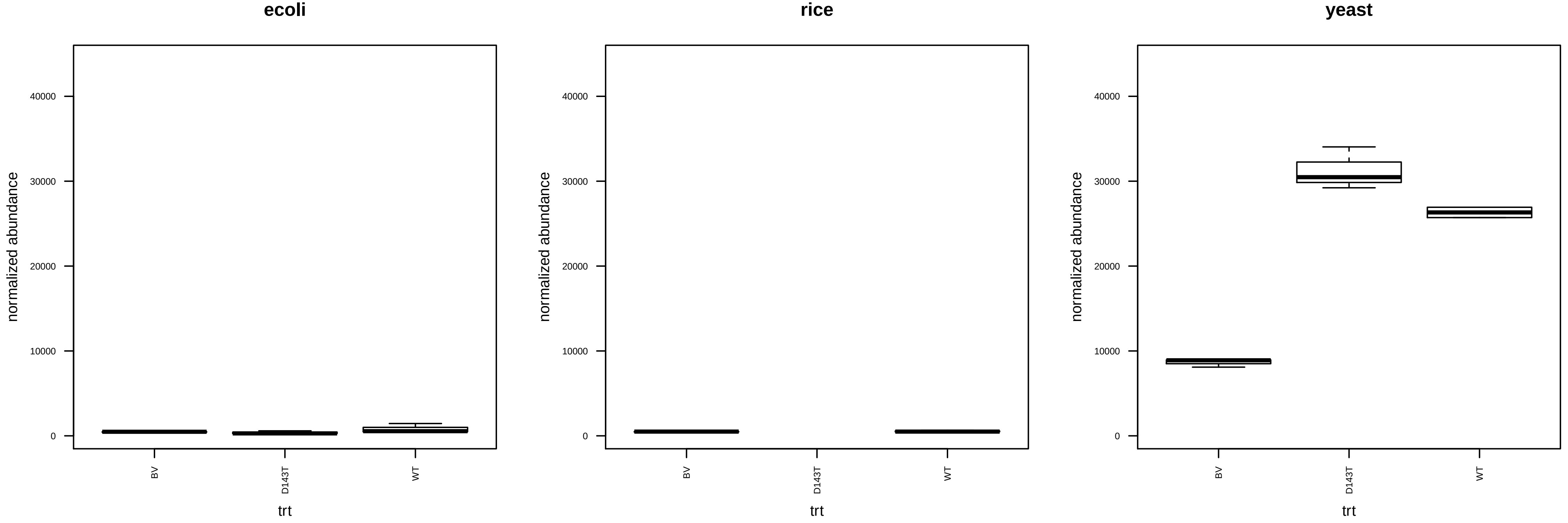

Guanosine

rt=399
C259: MSI conf = 1
notes:

	p−value
spp	< 1e−05
trt	1.11e−05
spp:trt	< 1e−05

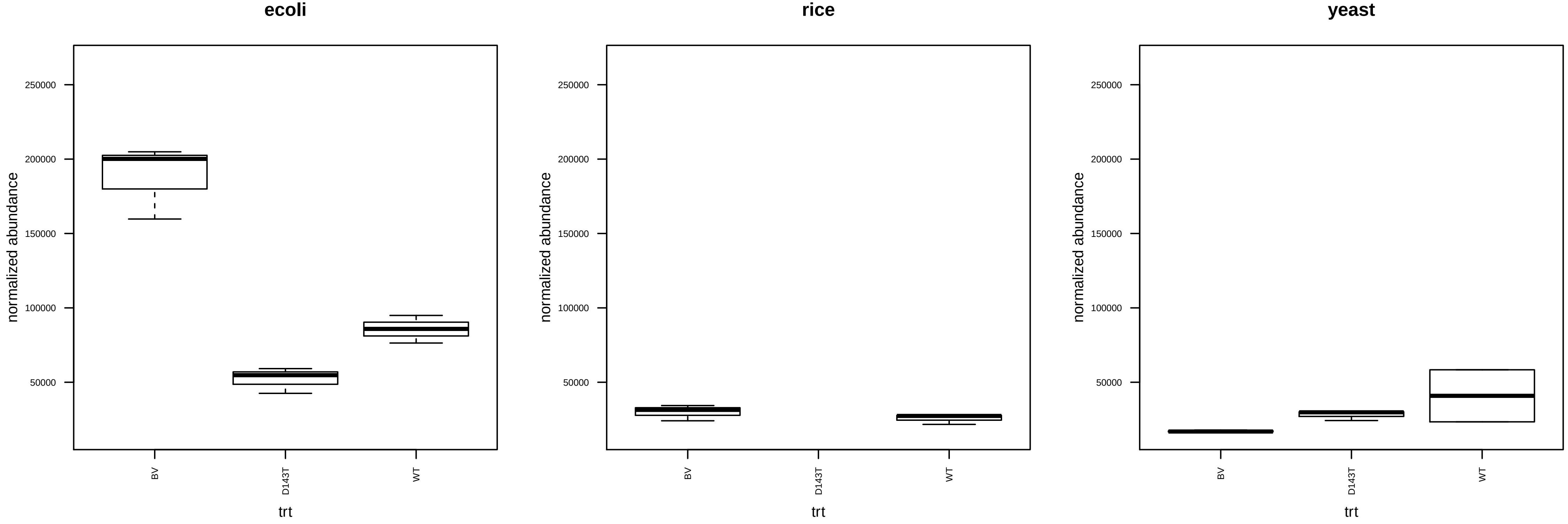

L−glutathione reduced

rt=416
C263: MSI conf = 1
notes:

	p−value
spp	< 1e−05
trt	< 1e−05
spp:trt	1.41e−05

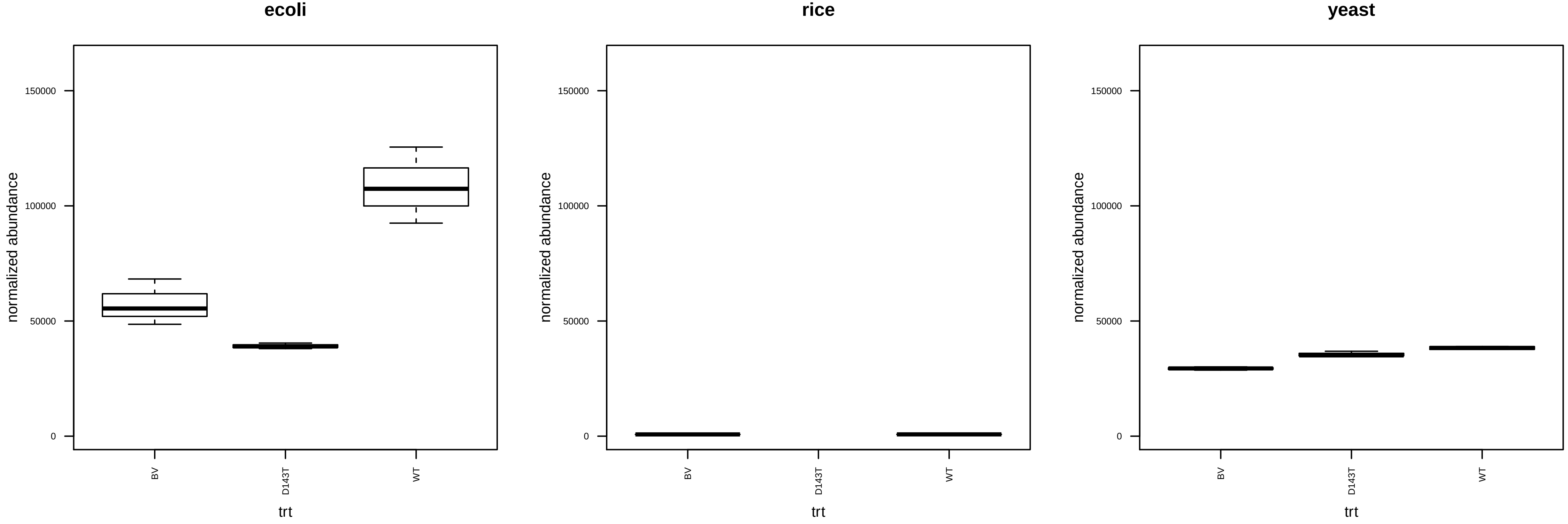

Sucrose

rt=584
C270: MSI conf = 1
notes:

	p−value
spp	< 1e−05
trt	1.5e−05
spp:trt	< 1e−05

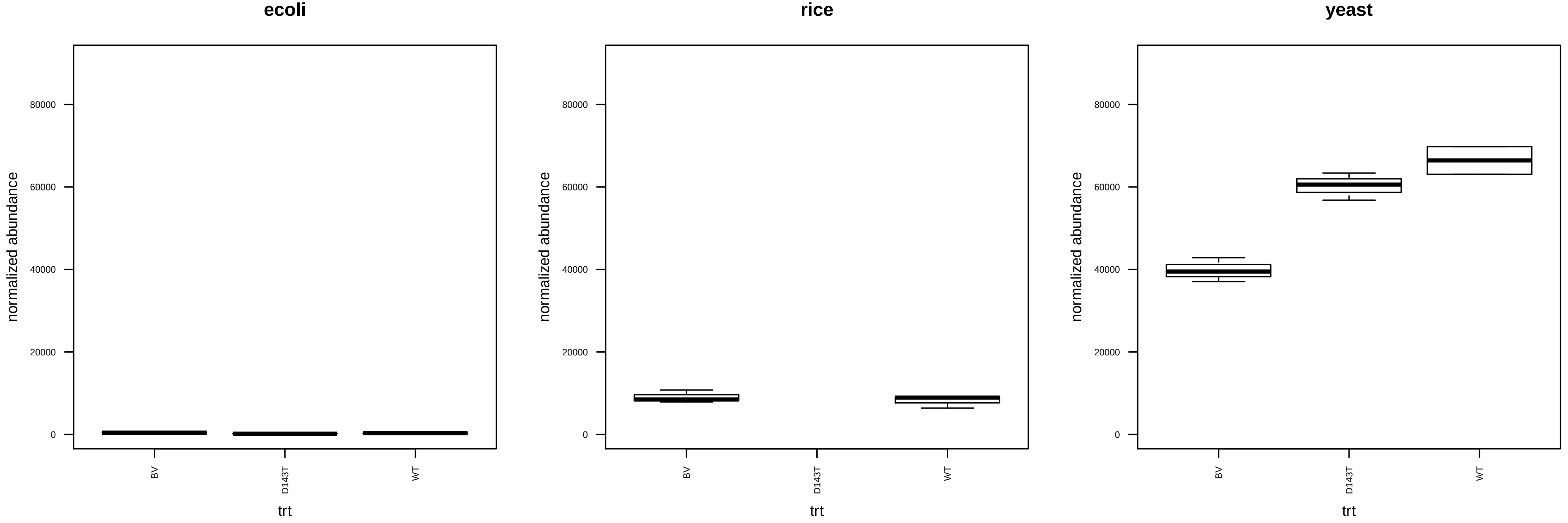

L−Lysine

rt=766
C273: MSI conf = 1
notes:

	p−value
spp	< 1e−05
trt	1.87e−05
spp:trt	< 1e−05

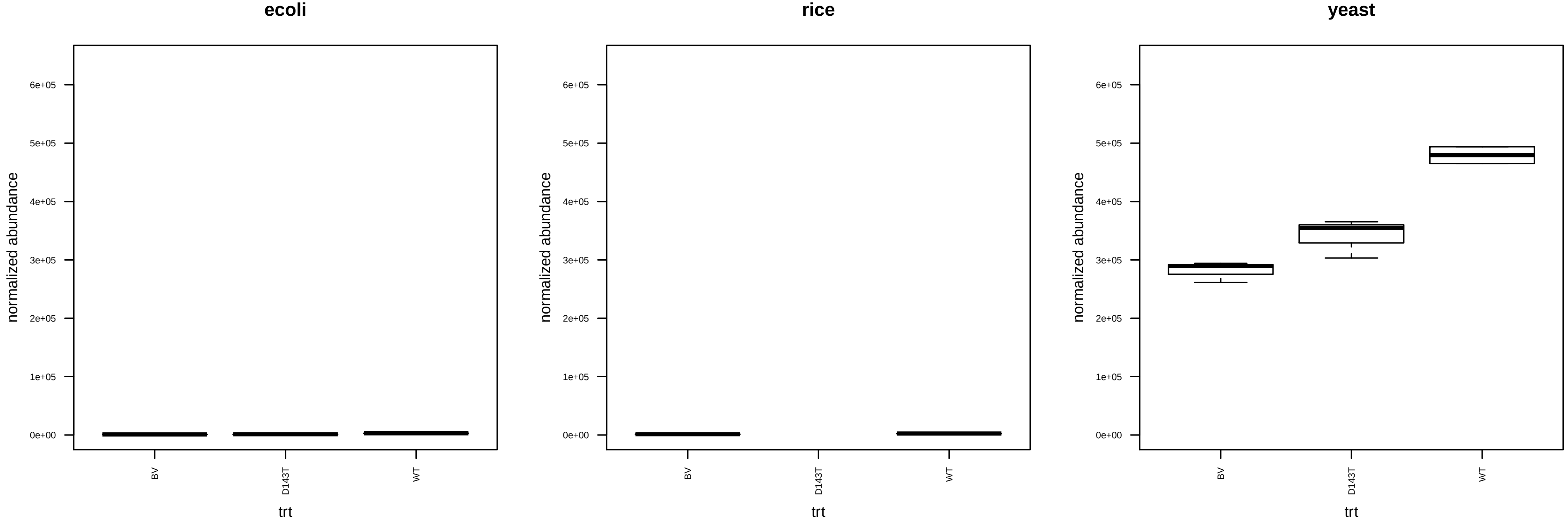

1−palmitoyl−2−linoleoyl−sn−glycero−3−phosphate (sodium salt)

rt=50
C298: MSI conf = 1
notes:

	p−value
spp	< 1e−05
trt	0.00627
spp:trt	0.08658

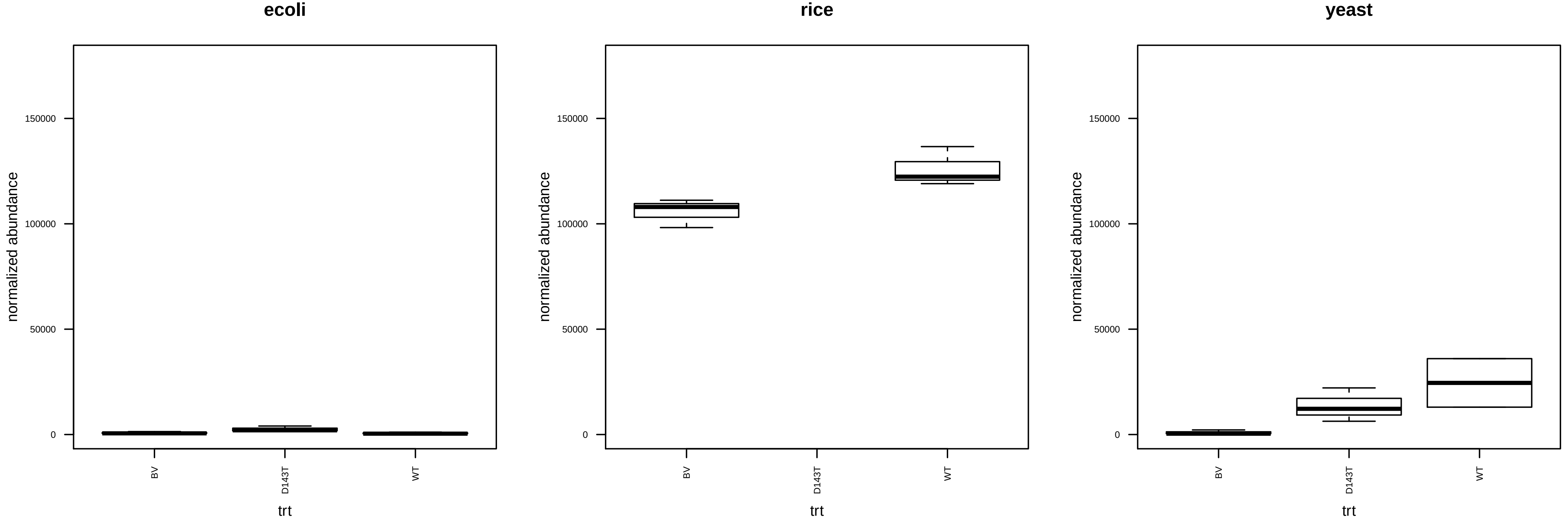

L−Aspartic acid

rt=436
C317: MSI conf = 1
notes:

	p−value
spp	<1e−05
trt	0.1442
spp:trt	0.0723

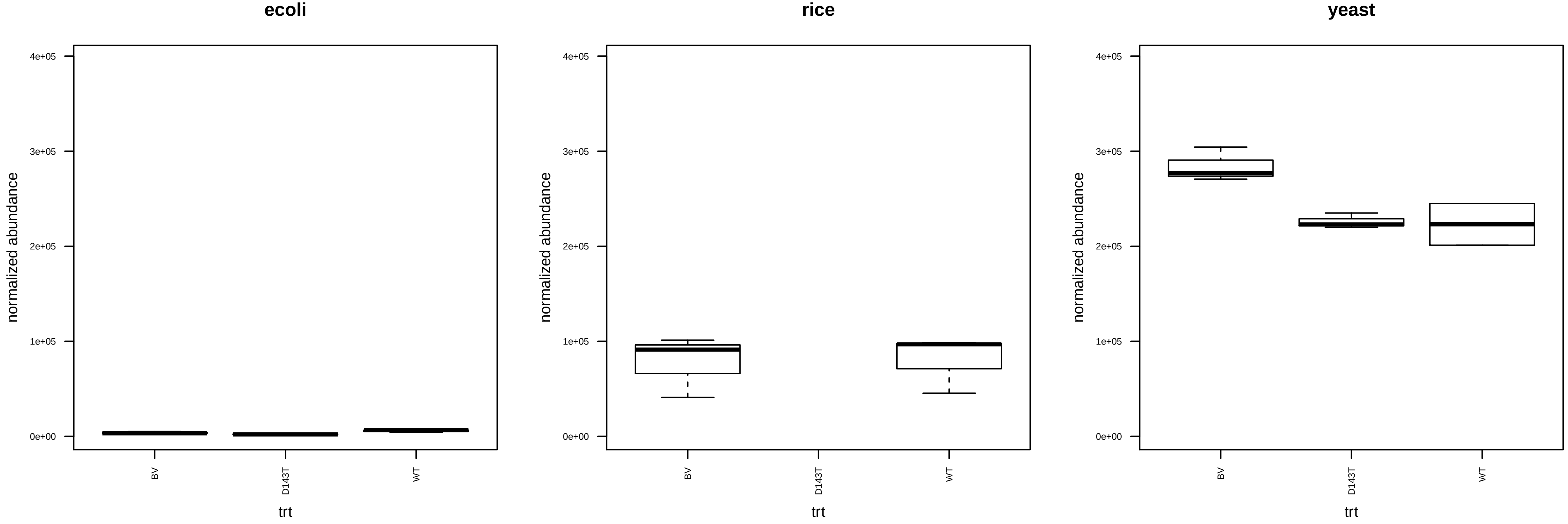

Uridine 5'−diphosphate−like

rt=598
C319: MSI conf = 3
notes:

	p−value
spp	<1e−05
trt	0.0431
spp:trt	0.0144

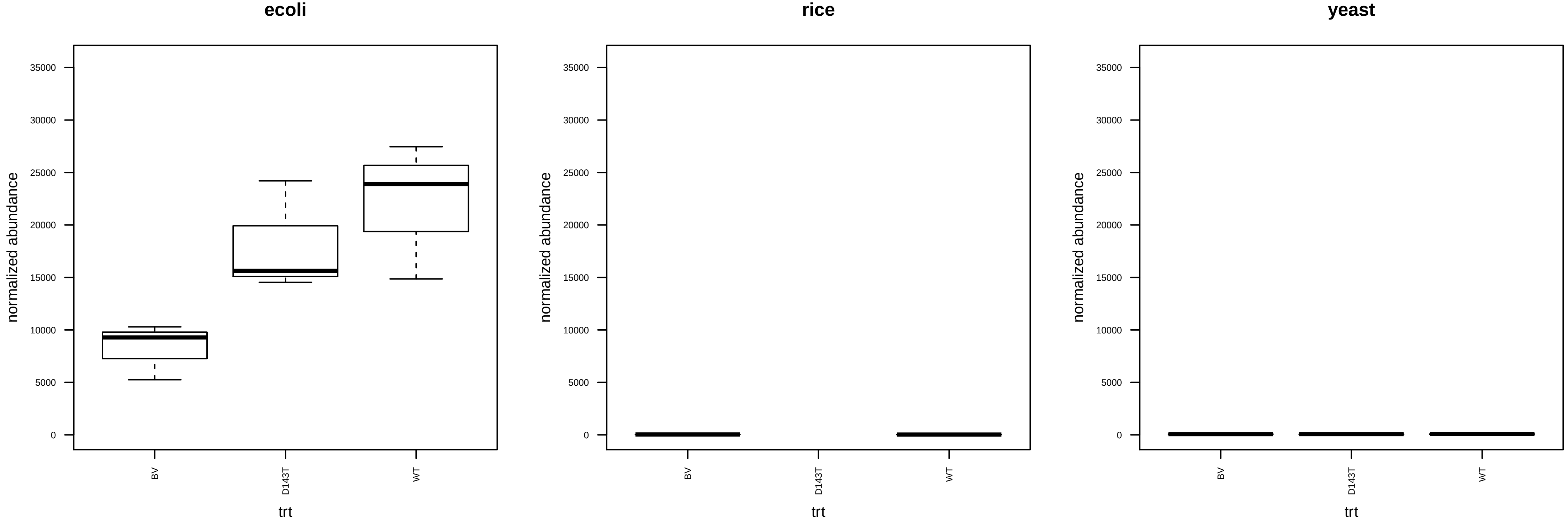

Pyrrolidonecarboxylic acid

rt=132
C358: MSI conf = 1
notes:

	p−value
spp	<1e−05
trt	0.987
spp:trt	1.000

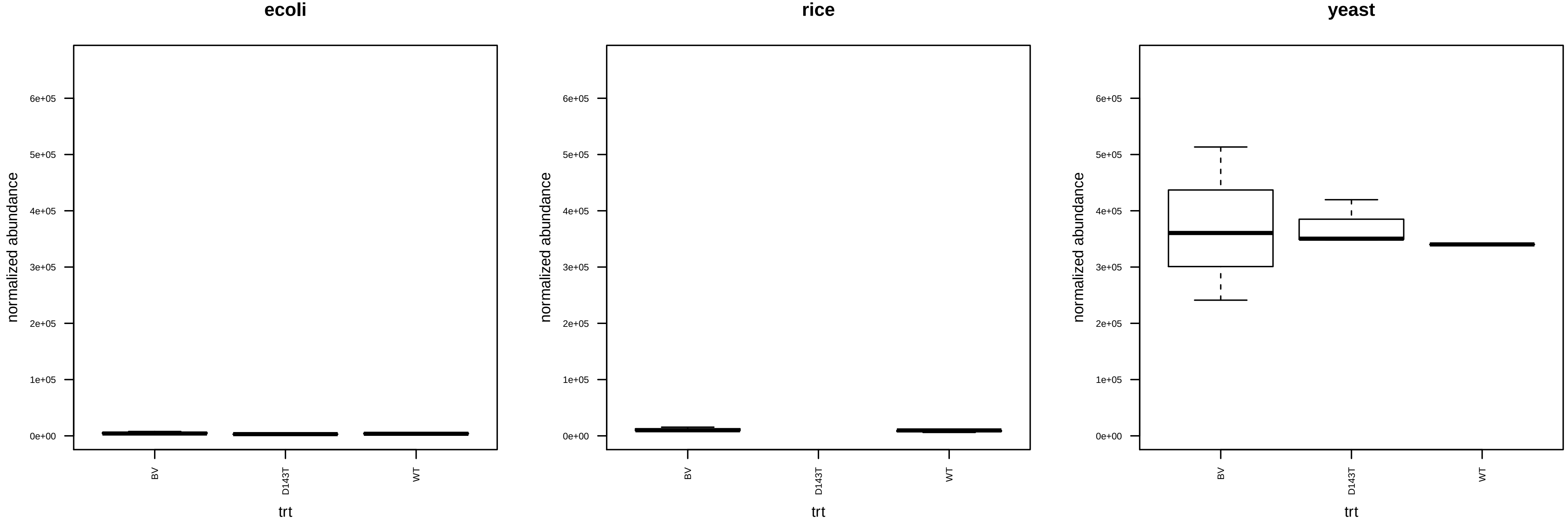

L−Saccharopine

rt=485
C360: MSI conf = 1
notes:

	p−value
spp	<1e−05
trt	<1e−05
spp:trt	<1e−05

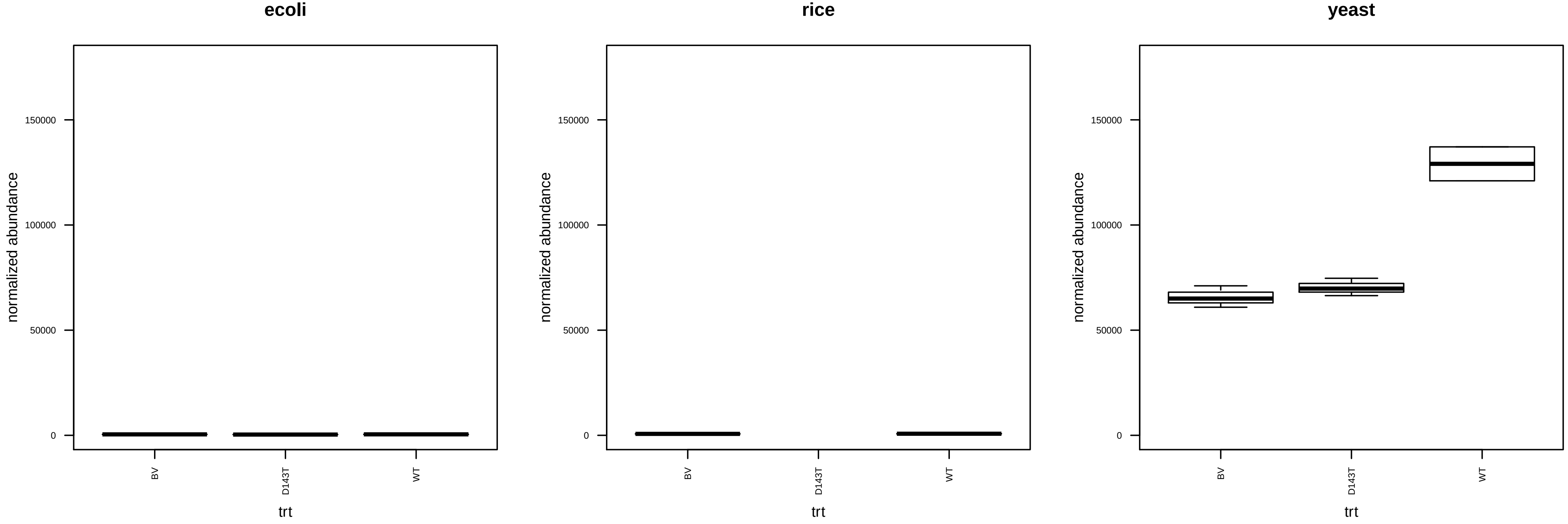

Deoxythymidine diphosphate−l−rhamnose

rt=404
C362: MSI conf = 4
notes: Metlin:58402

	p−value
spp	<1e−05
trt	<1e−05
spp:trt	<1e−05

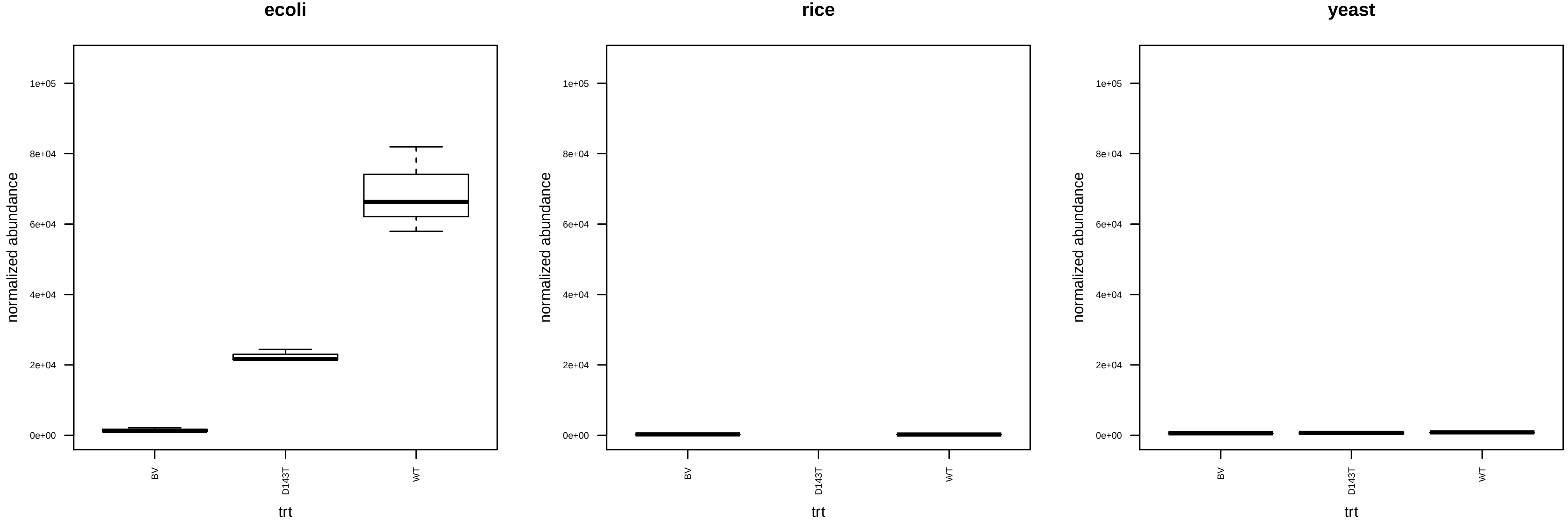

Malic acid

rt=443
C365: MSI conf = 1
notes:

	p−value
spp	<1e−05
trt	0.0941
spp:trt	0.0488

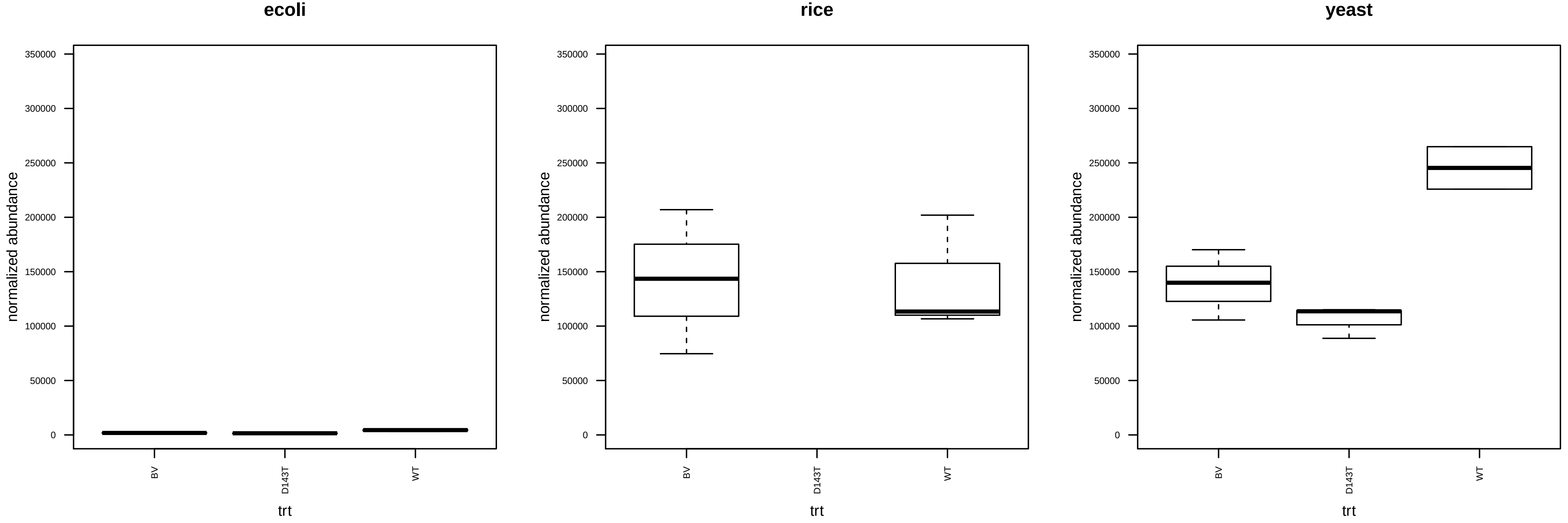

Cytidine

rt=359
C367: MSI conf = 1
notes:

	p−value
spp	< 1e−05
trt	4.46e−05
spp:trt	< 1e−05

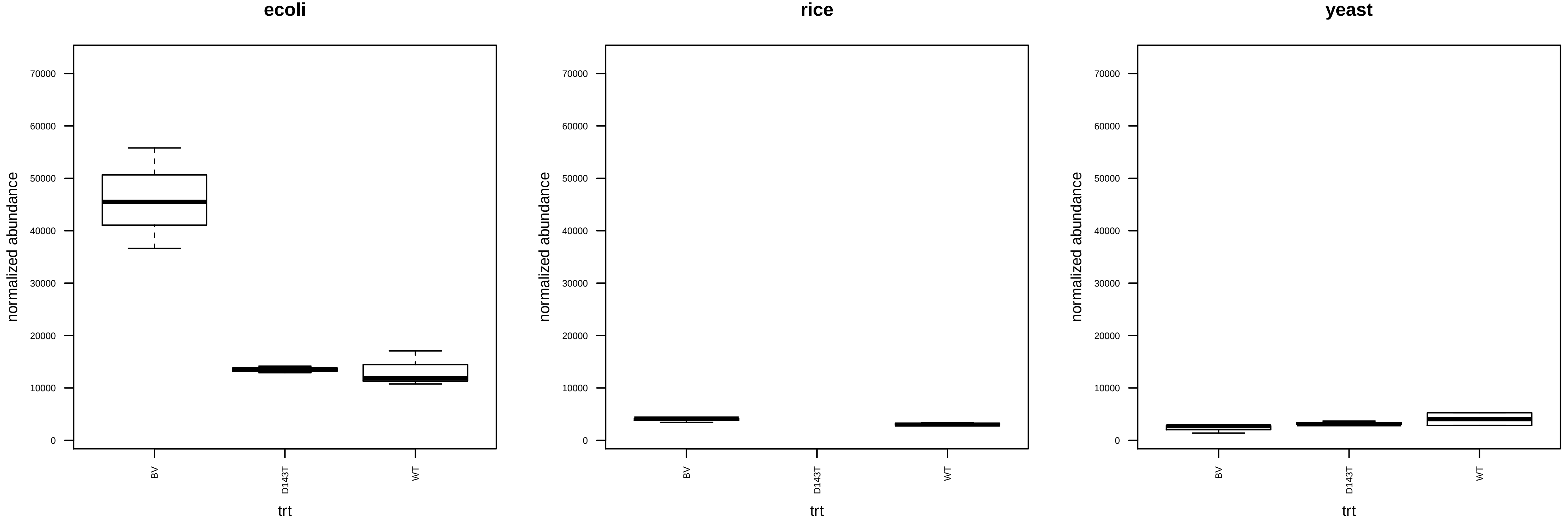

Uridine diphosphate glucose

rt=506
C368: MSI conf = 1
notes:

	p−value
spp	< 1e−05
trt	0.006588
spp:trt	0.000163

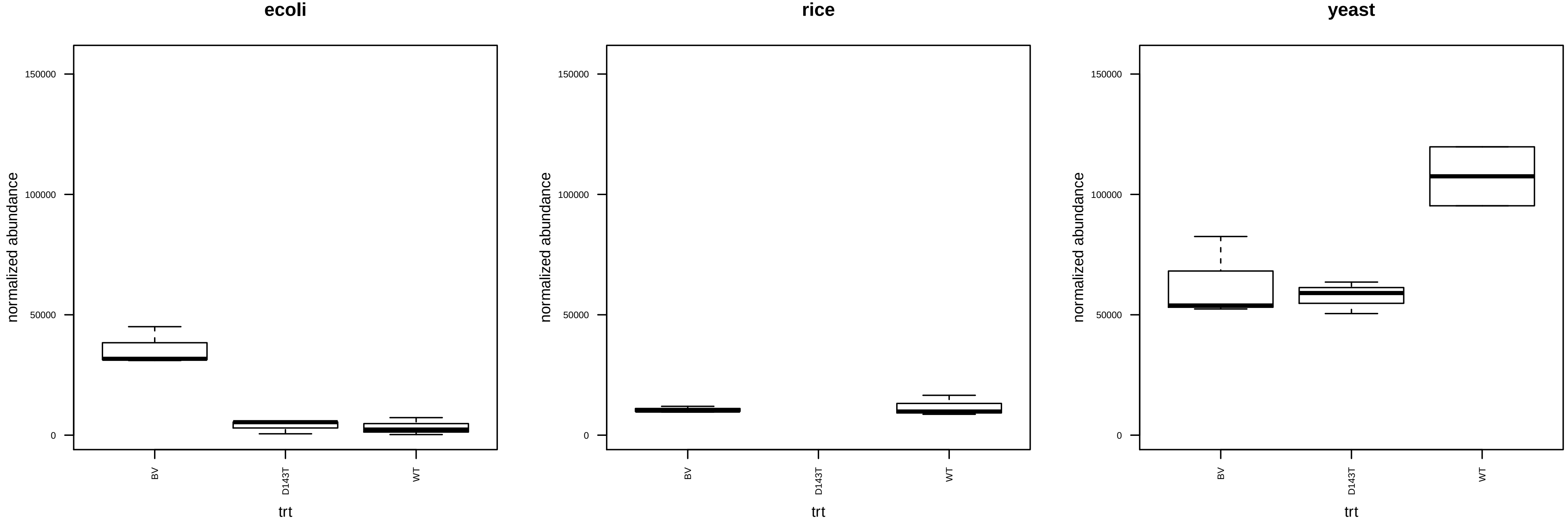

dTDP−D−fucose

rt=386
C443: MSI conf = 3
notes:

	p−value
spp	< 1e−05
trt	6.02e−05
spp:trt	1.01e−05

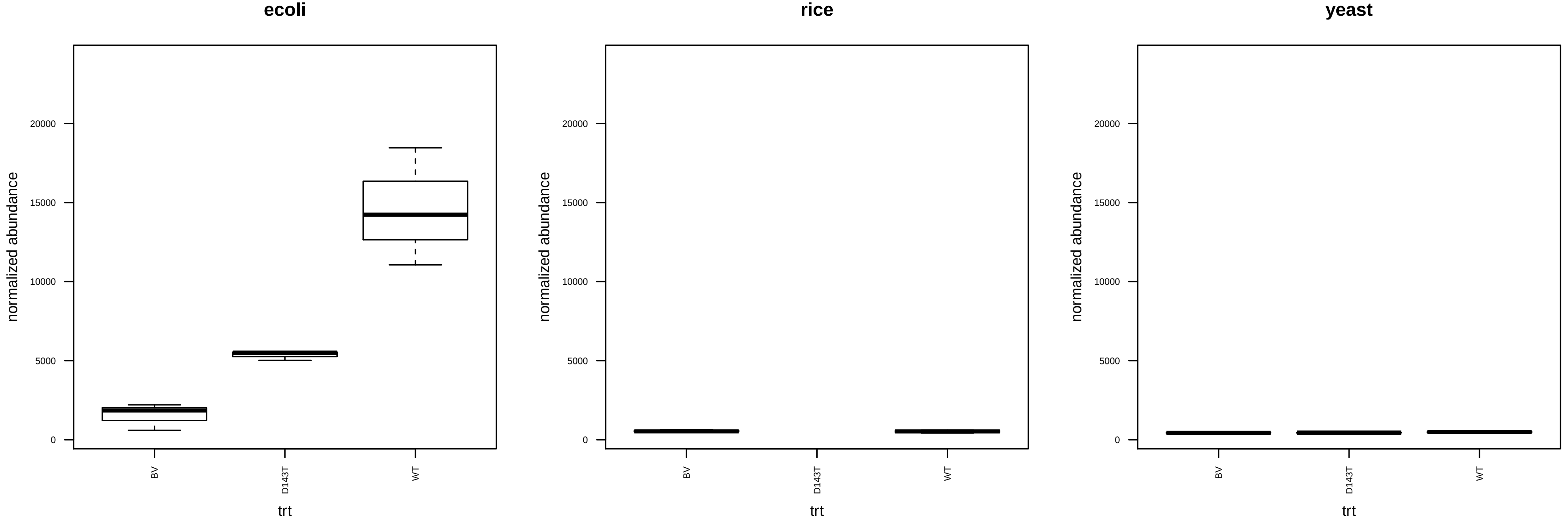

Gluconic acid

rt=371
C447: MSI conf = 1
notes:

	p−value
spp	< 1e−05
trt	0.004860
spp:trt	0.000296

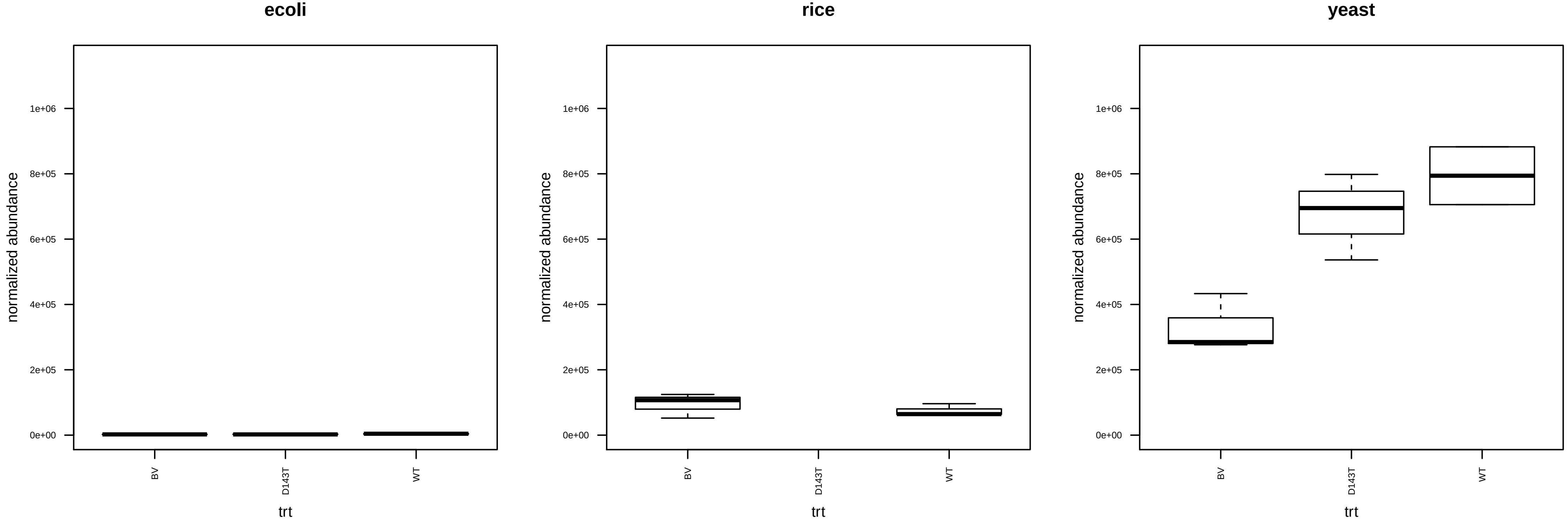

Galactonic acid

rt=370
C448: MSI conf = 1
notes:

	p−value
spp	< 1e−05
trt	0.00359
spp:trt	0.01212

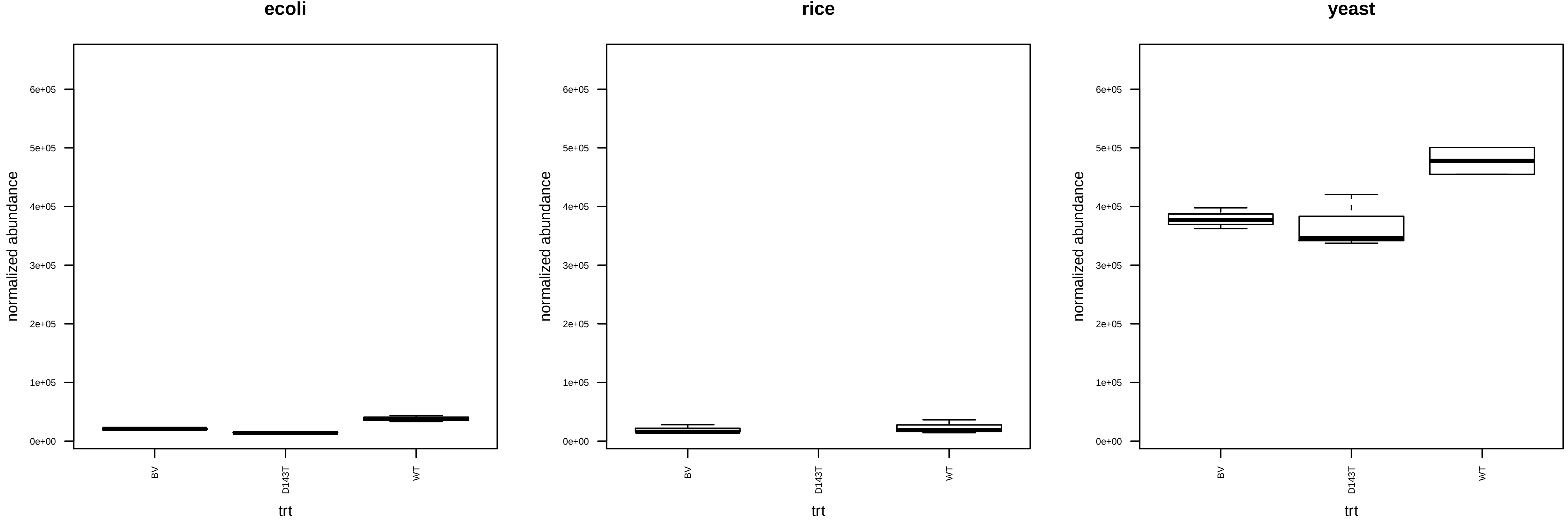

L−Glutamic acid

rt=96
C482: MSI conf = 2
notes:

	p−value
spp	<1e−05
trt	0.879
spp:trt	0.838

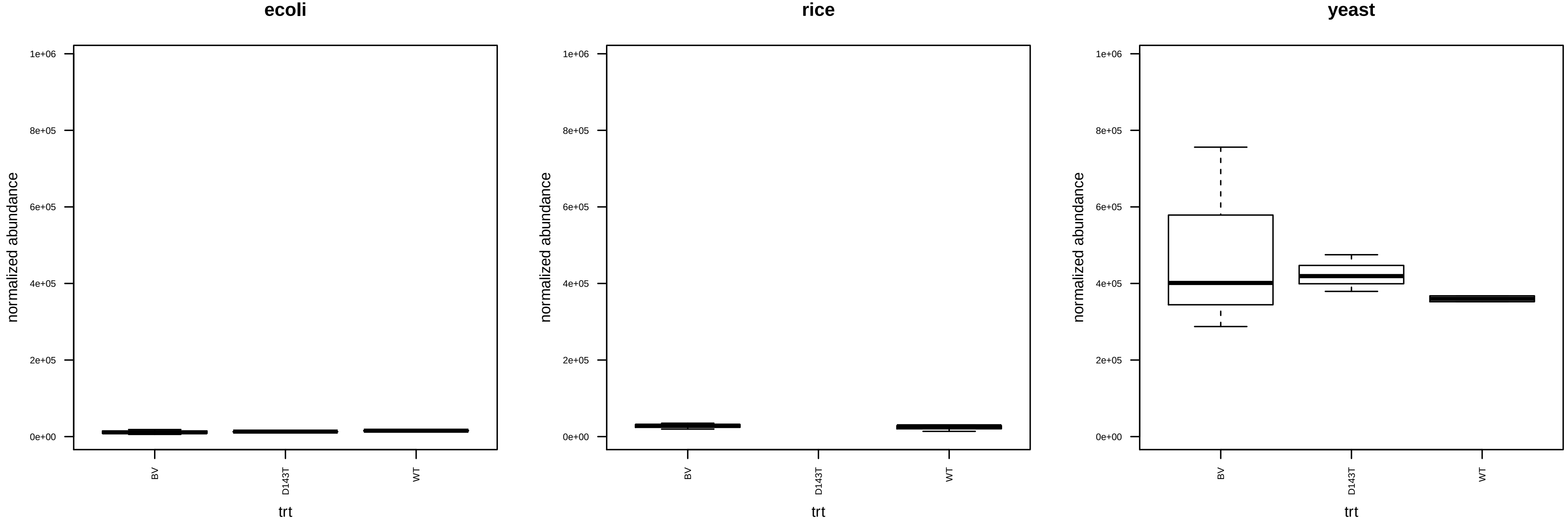

L−Tyrosine

rt=399
C555: MSI conf = 1
notes:

	p−value
spp	< 1e−05
trt	1.03e−05
spp:trt	9.63e−05

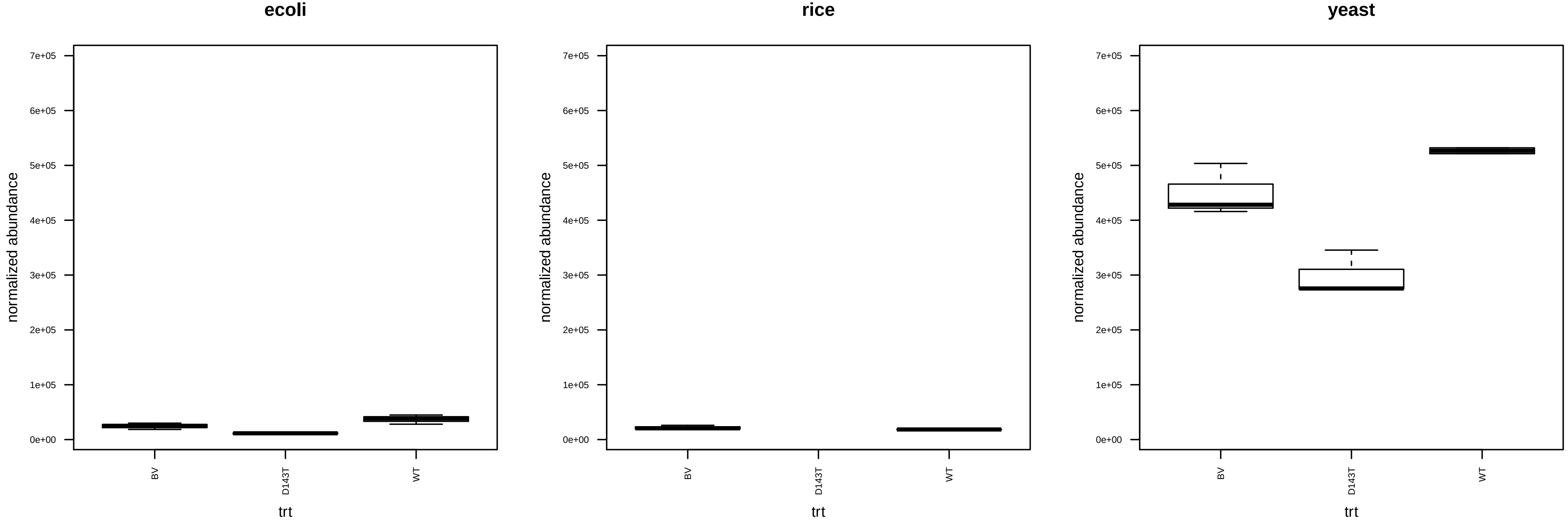

Uridine 5−Monophosphate (UMP)

rt=437
C558: MSI conf = 1
notes:

	p−value
spp	< 1e−05
trt	0.00128
spp:trt	5.85e−05

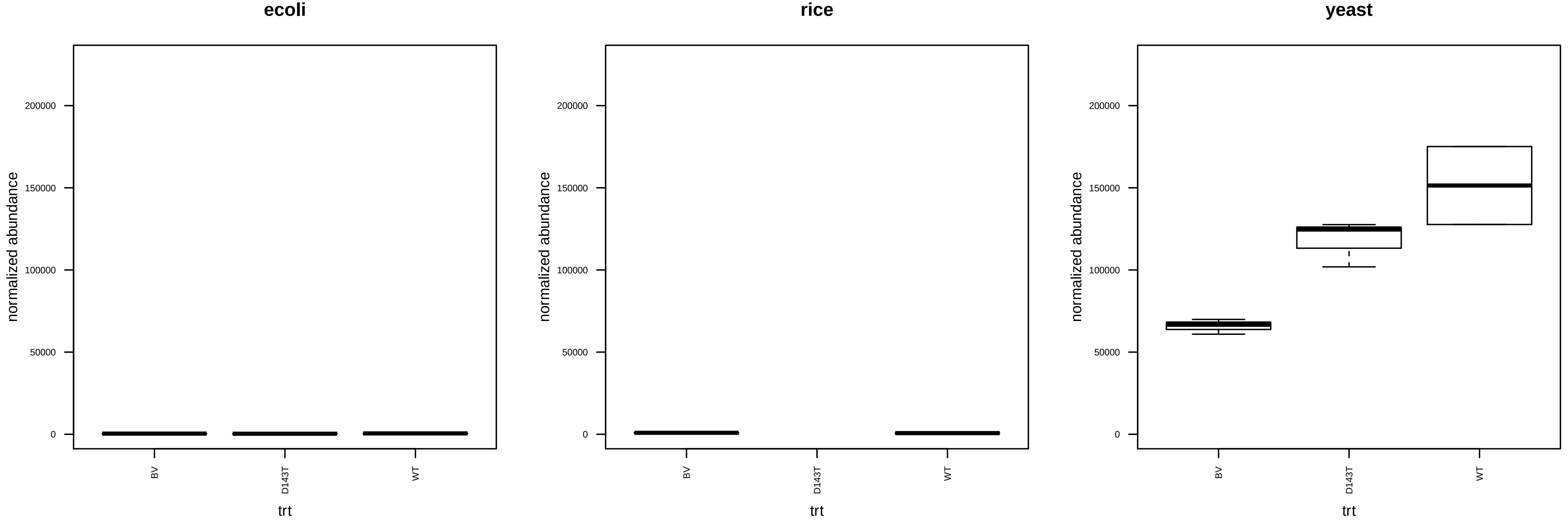

NADP (split signal)

rt=544
C562: MSI conf = 2
notes:

	p-value
spp	0.000504
trt	< 1e−05
spp:trt	1.88e−05

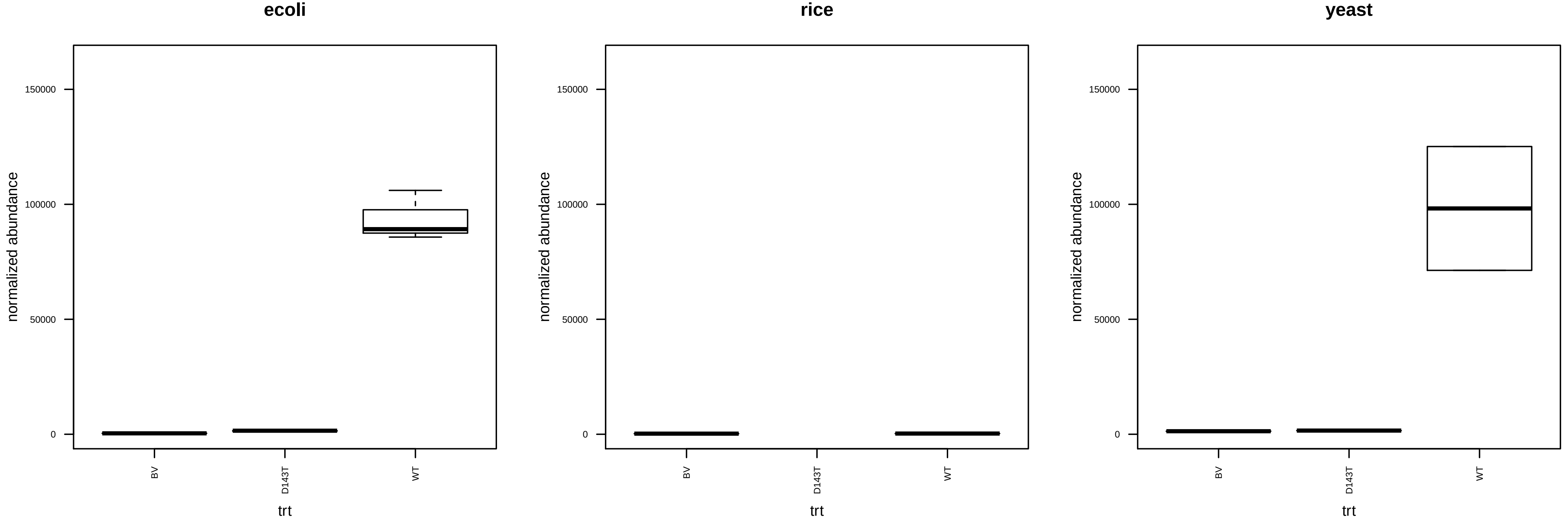

DL−isoleucine

rt=338
C570: MSI conf = 1
notes:

	p−value
spp	< 1e−05
trt	0.000181
spp:trt	0.008853

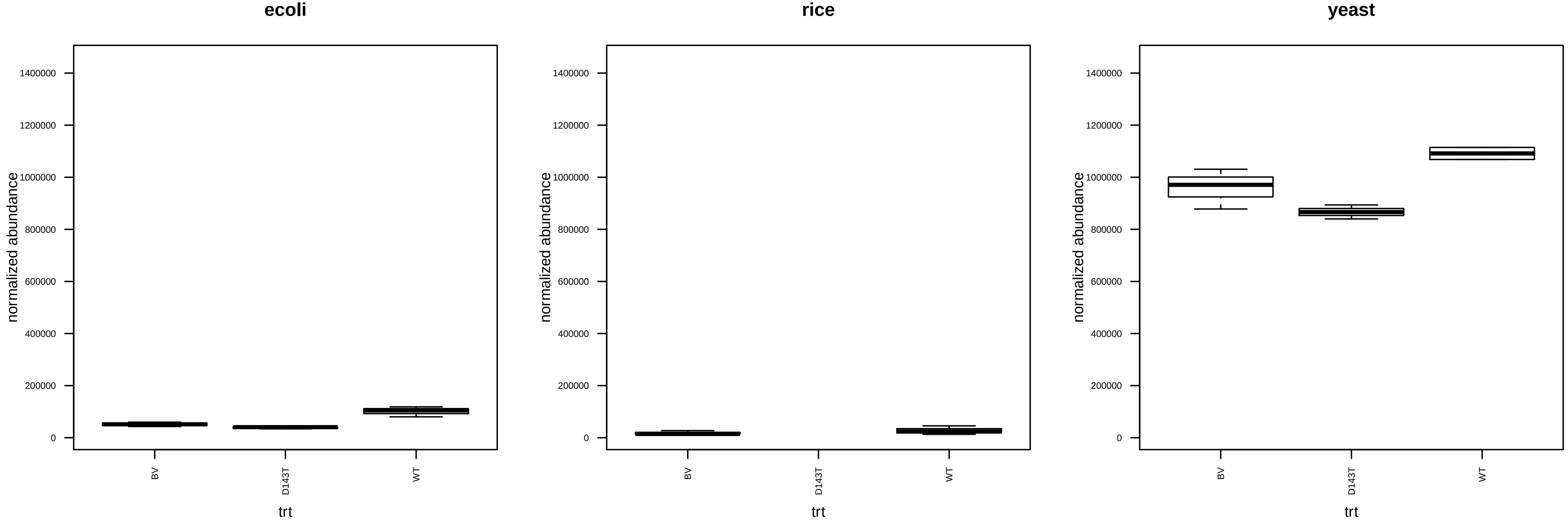

Succinic acid

rt=214
C630: MSI conf = 1
notes:

	p−value
spp	< 1e−05
trt	0.00163
spp:trt	0.05548

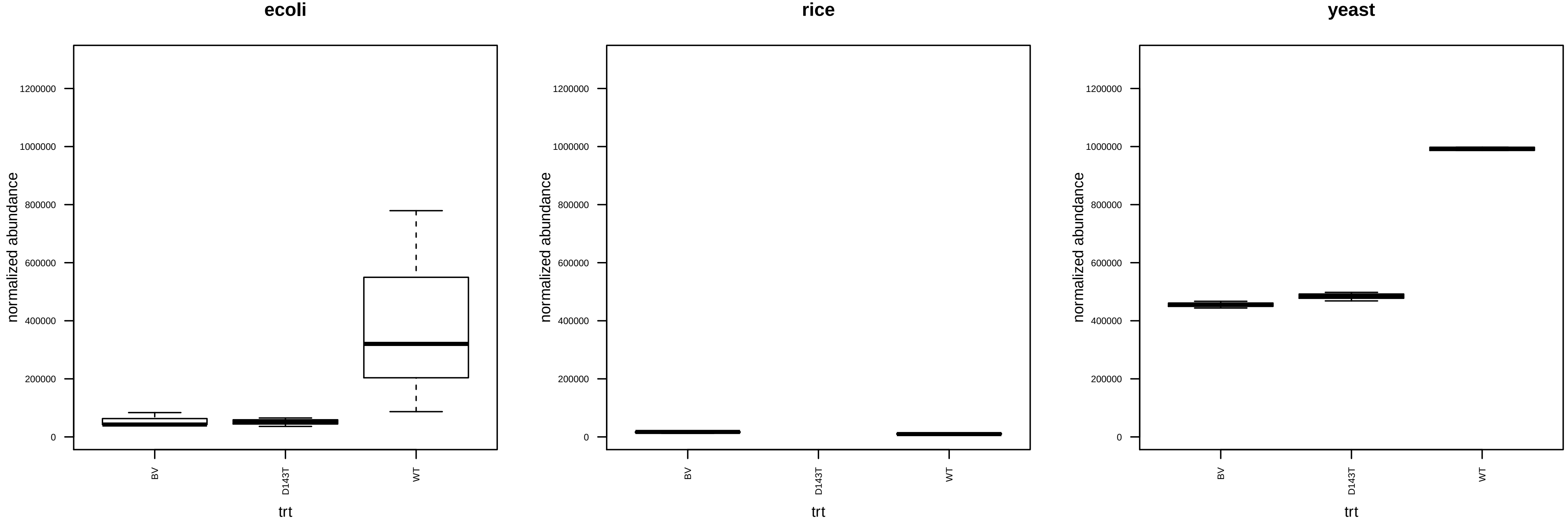

NADP (split signal)

rt=544
C671: MSI conf = 4
notes:

	p−value
spp	0.04262
trt	2.08e−05
spp:trt	0.00176

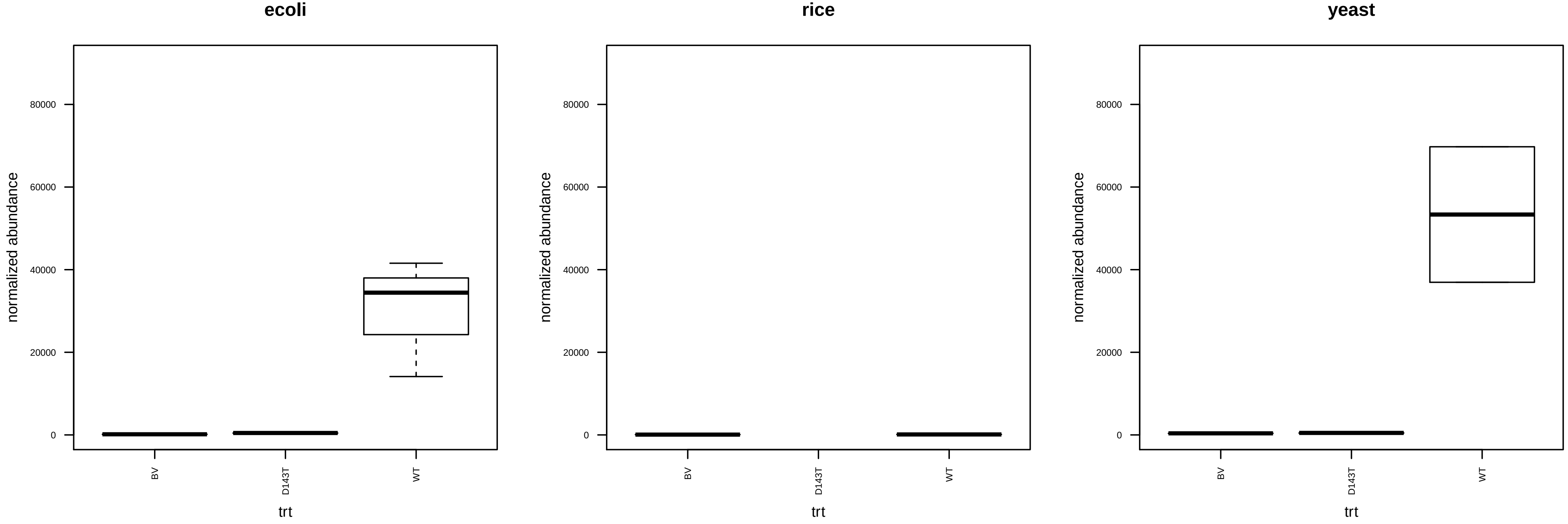

L−Leucine

rt=327
C683: MSI conf = 3
notes:

	p−value
spp	< 1e−05
trt	5.55e−05
spp:trt	0.00462

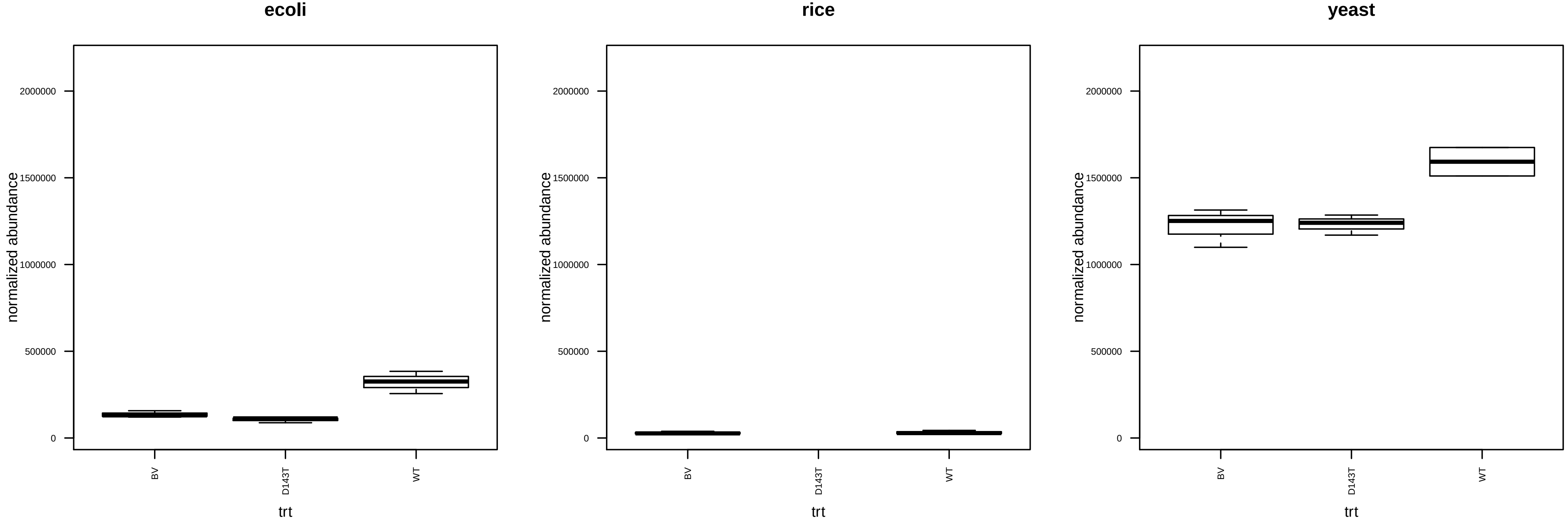

Uridine

rt=262
C702: MSI conf = 1
notes:

	p−value
spp	< 1e−05
trt	0.770322
spp:trt	0.000471

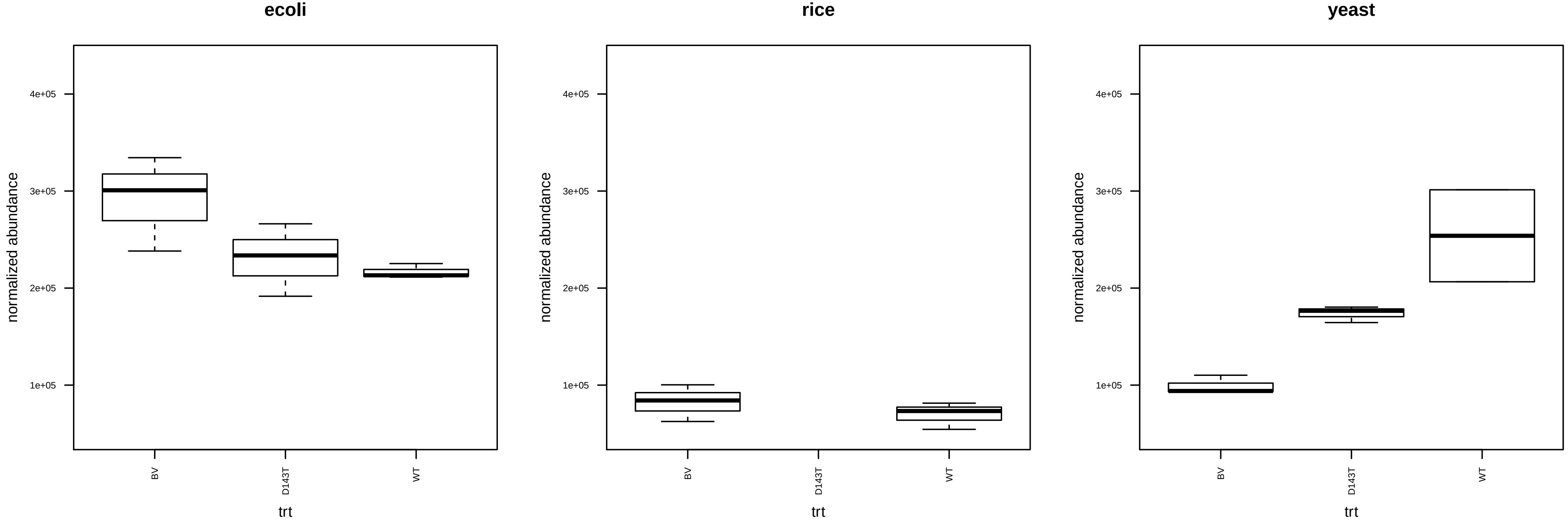

Inosine

rt=325
C858: MSI conf = 1
notes:

	p−value
**spp**	< 1e−05
**trt**	0.00262
**spp:trt**	0.70457

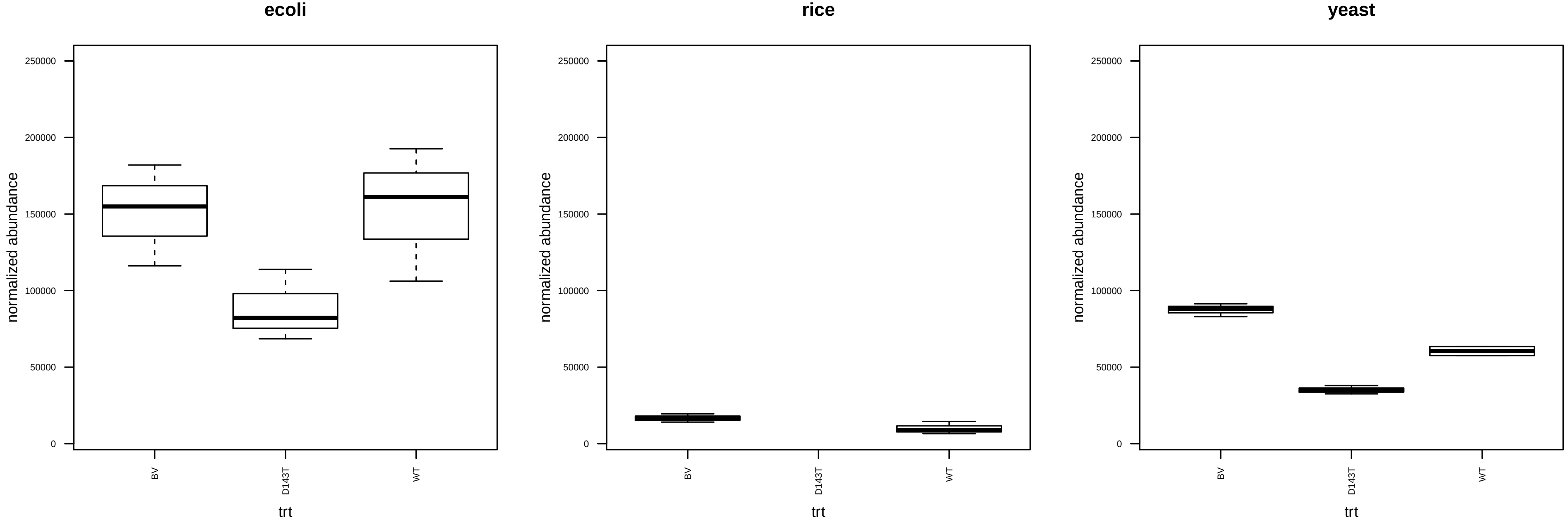

Sodium lauryl sulfate

rt=47
C945: MSI conf = 1
notes:

	p−value
**spp**	0.209
**trt**	0.812
**spp:trt**	0.702

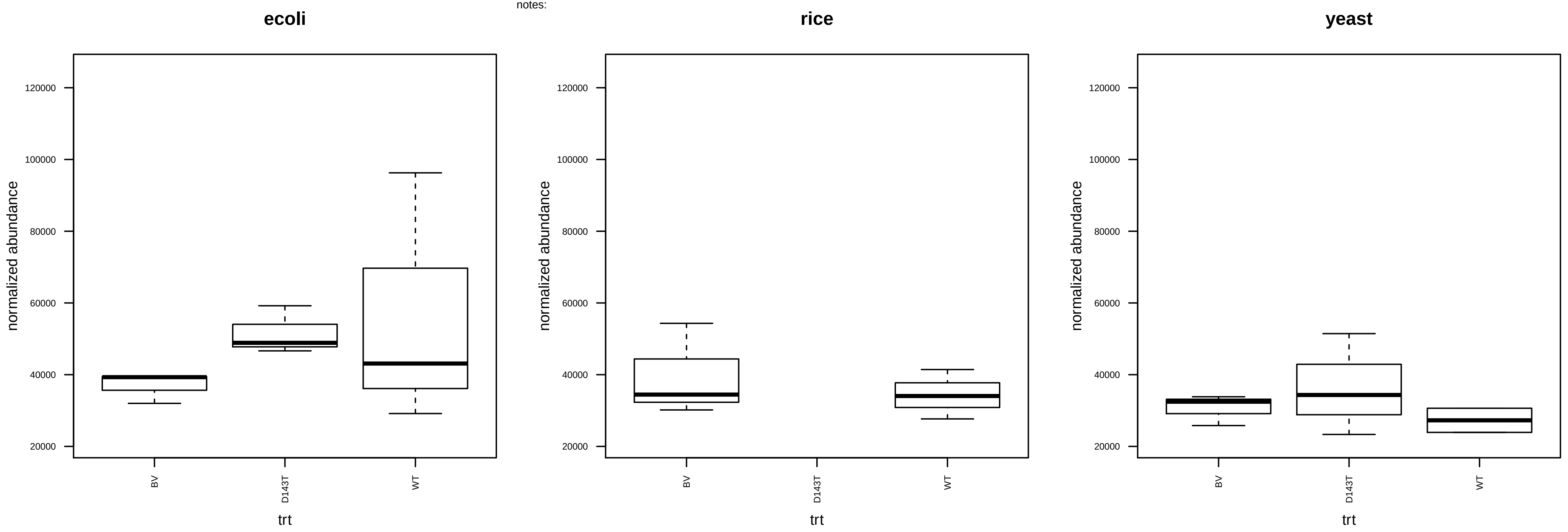

3−Hydroxymethylglutaric acid

rt=223
C971: MSI conf = 1
notes:

	p−value
spp	<1e−05
trt	1.00
spp:trt	0.96

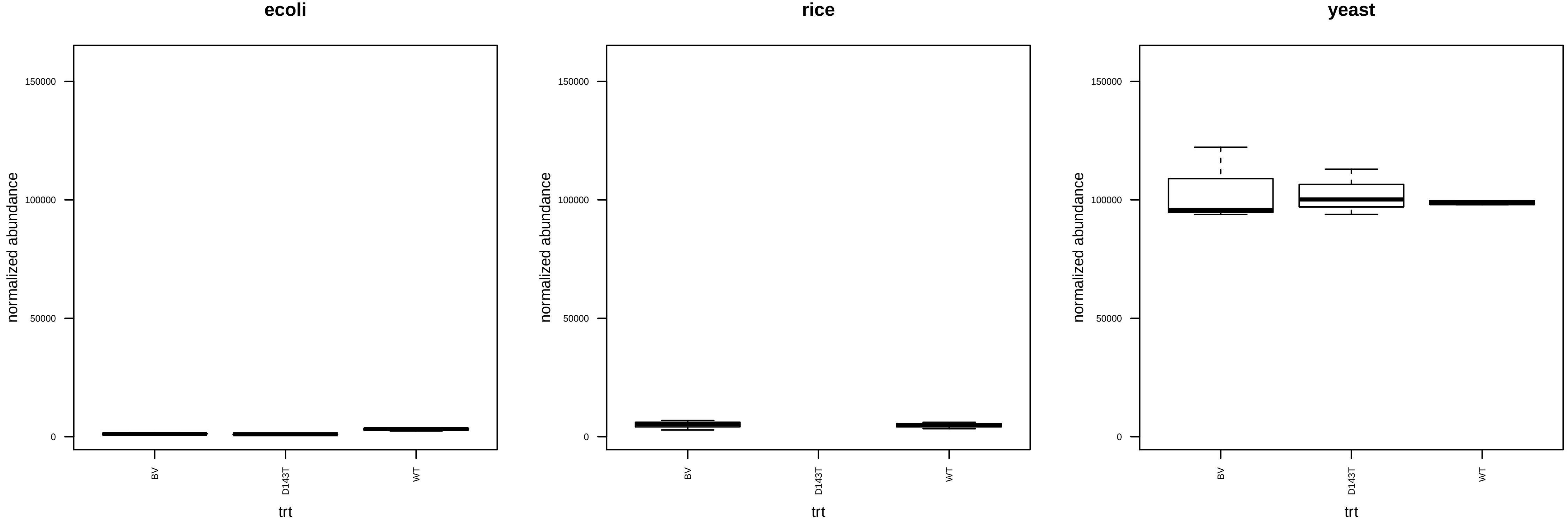

Pantothenic acid

rt=89
C1120: MSI conf = 1
notes:

	p−value
spp	<1e−05
trt	<1e−05
spp:trt	<1e−05

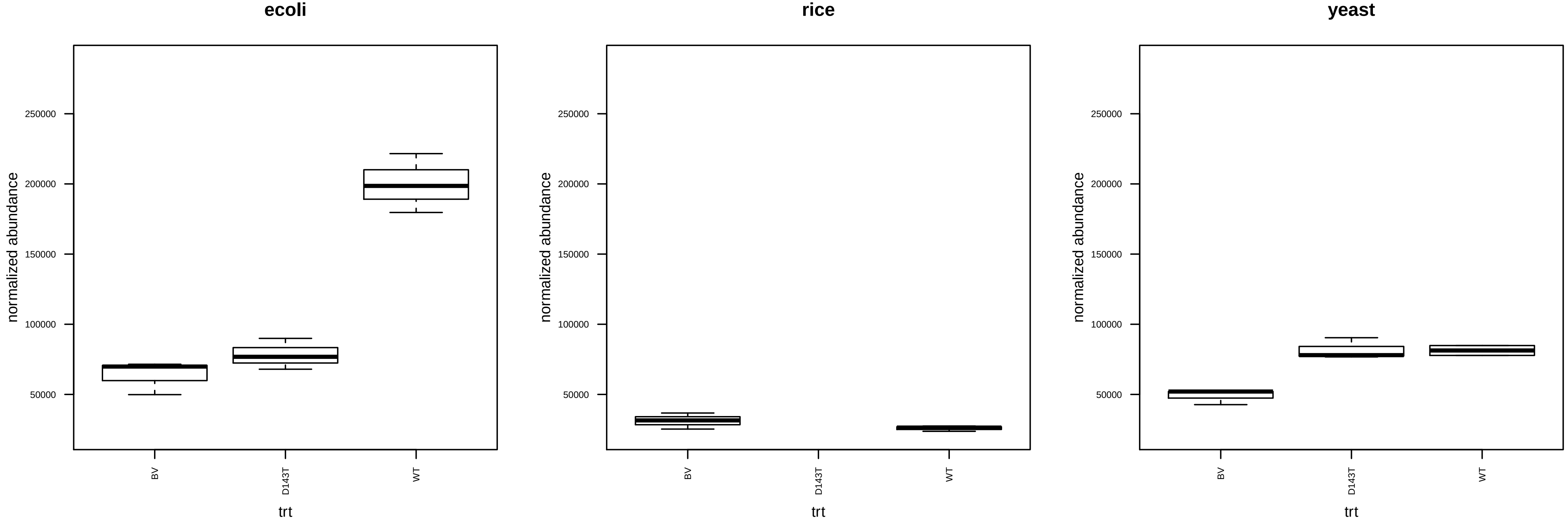

3'−Dephospho coenzyme A

rt=92
C1122: MSI conf = 3
notes:

	p−value
spp	<1e−05
trt	<1e−05
spp:trt	<1e−05

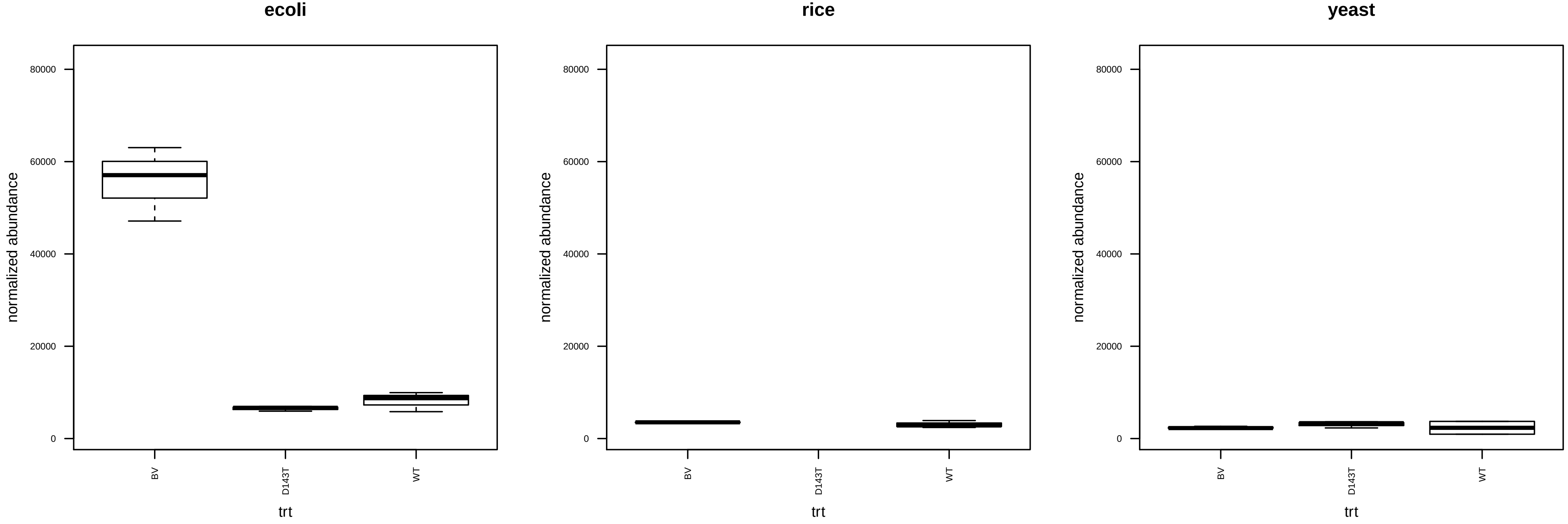

Indoxyl sulfate

rt=68
C1161: MSI conf = 1
notes:

	p−value
**spp**	0.00316
**trt**	0.03927
**spp:trt**	0.53151

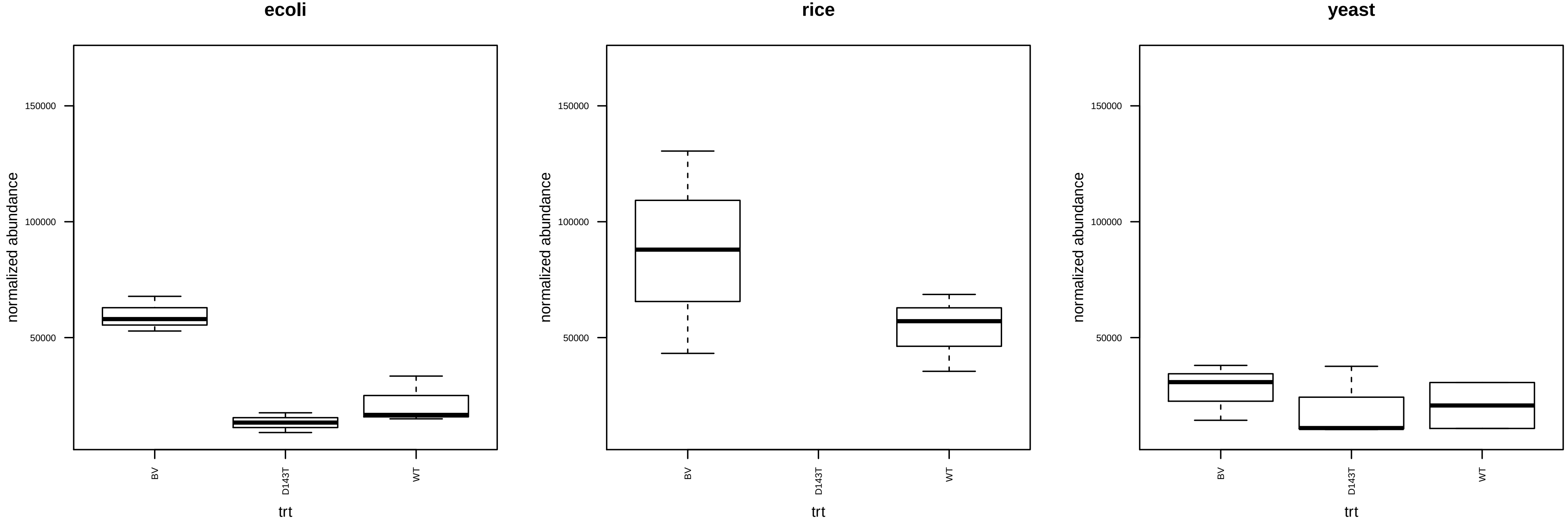

3−Methylxanthine

rt=211
C1220: MSI conf = 1
notes:

	p−value
spp	2.46e−05
trt	0.972
spp:trt	1.000

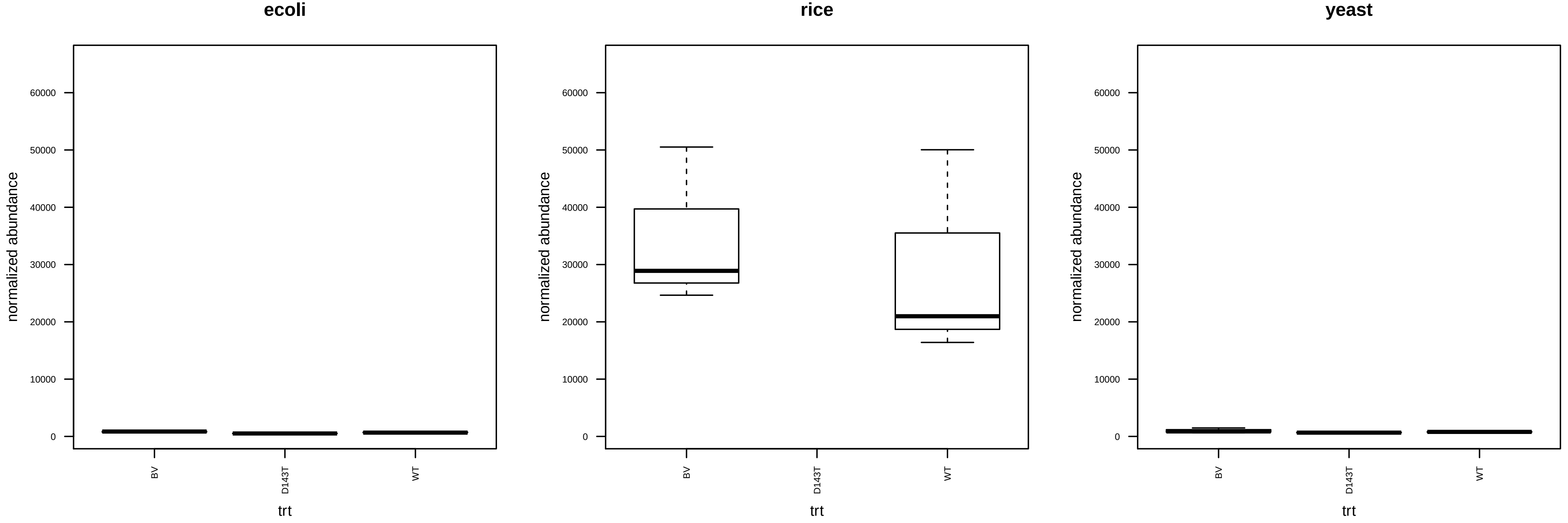

Pyroglutamic acid

rt=115
C1230: MSI conf = 1
notes:

	p−value
spp	< 1e−05
trt	9.15e−05
spp:trt	5.21e−05

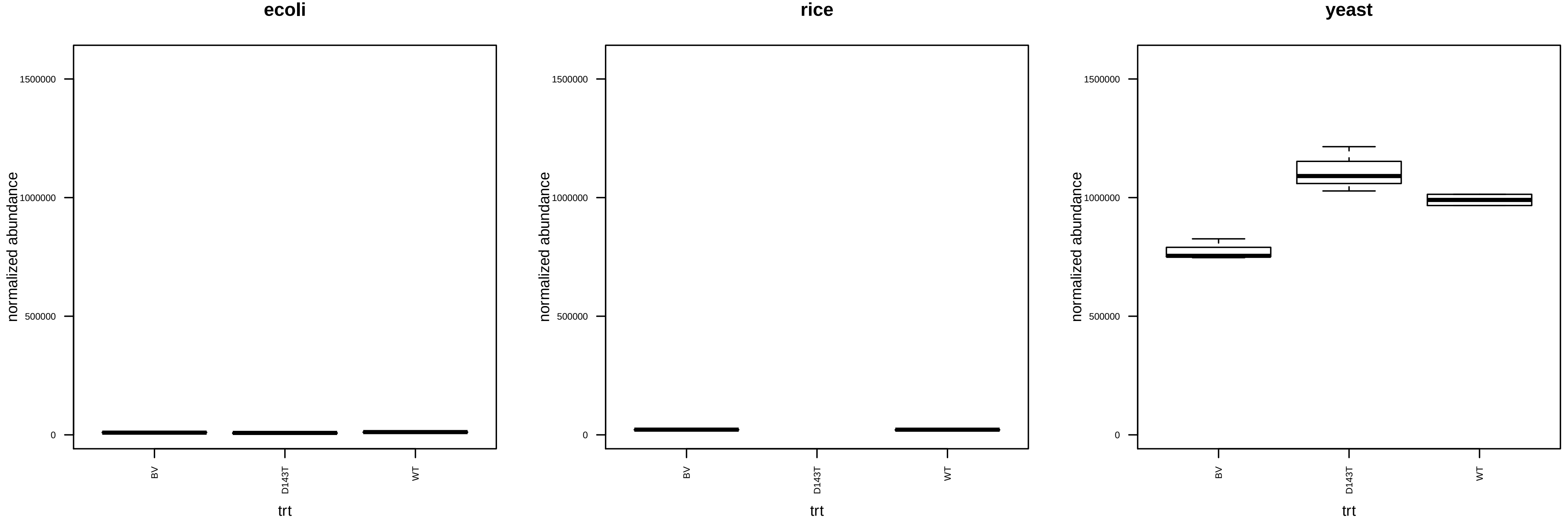

Thymidine

rt=133
C1270: MSI conf = 1
notes:

	p−value
spp	< 1e−05
trt	0.00492
spp:trt	0.00153

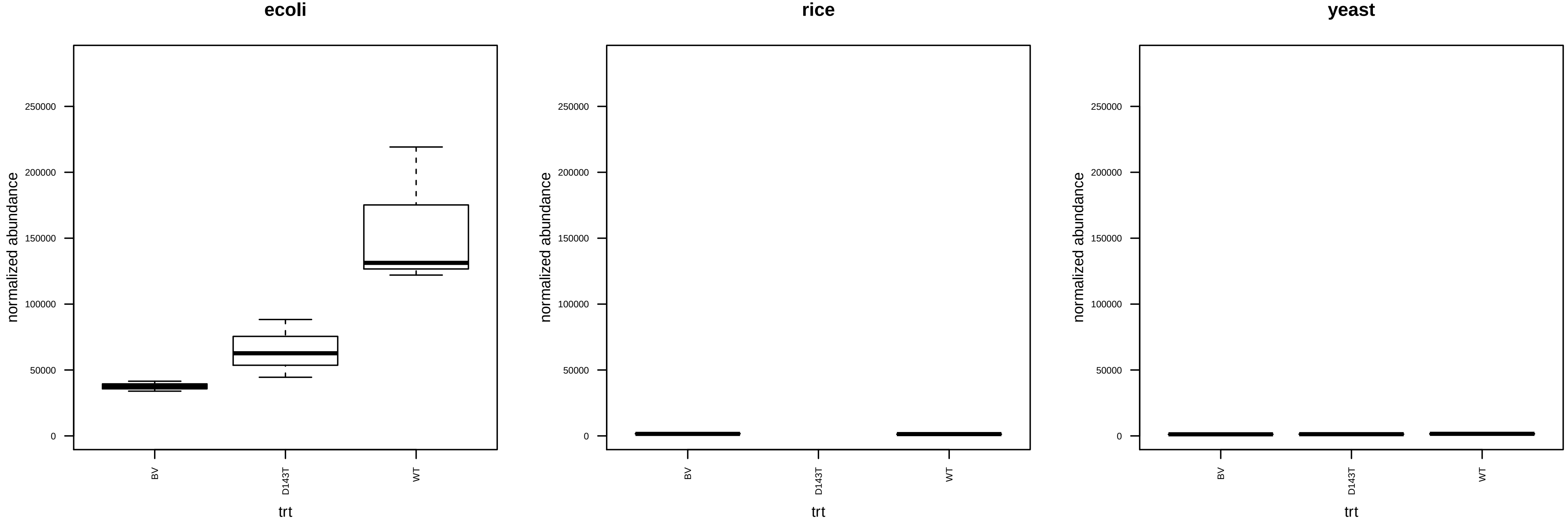

Pyroglutamic acid

rt=142
C1277: MSI conf = 1
notes:

	p−value
**spp**	< 1e−05
**trt**	0.01370
**spp:trt**	0.00476

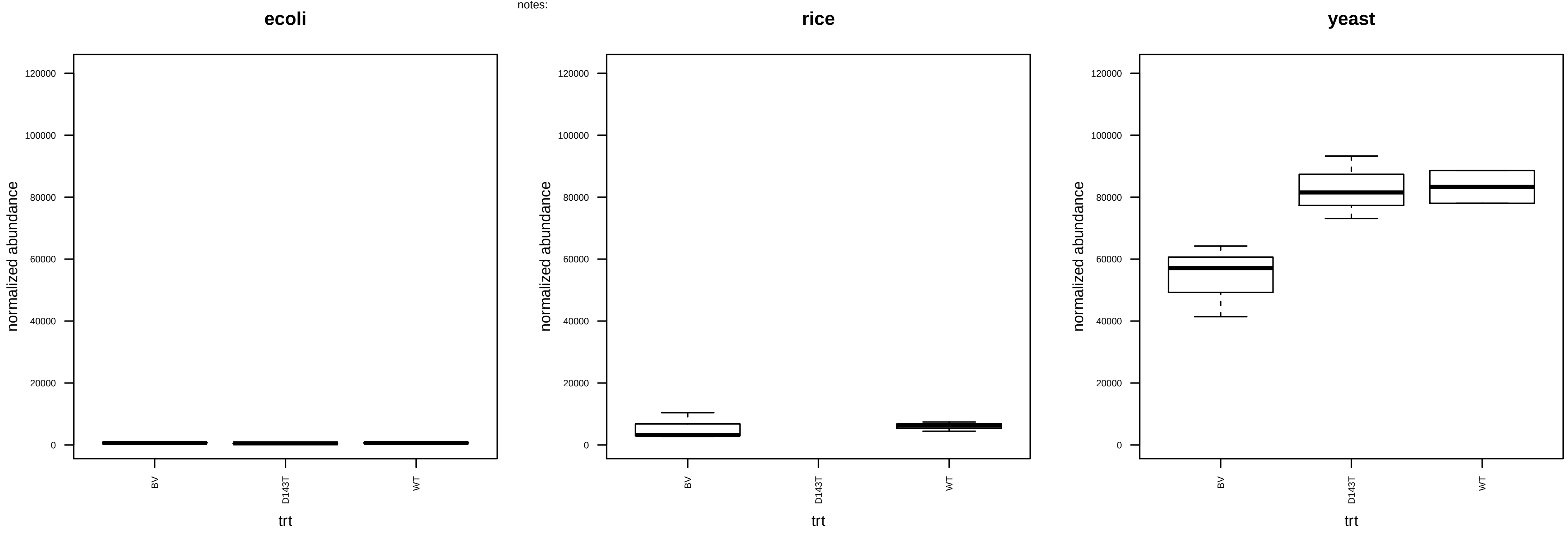

Glyceric acid

rt=180
C1534: MSI conf = 1
notes:

	p−value
**spp**	<1e−05
**trt**	0.1384
**spp:trt**	0.0465

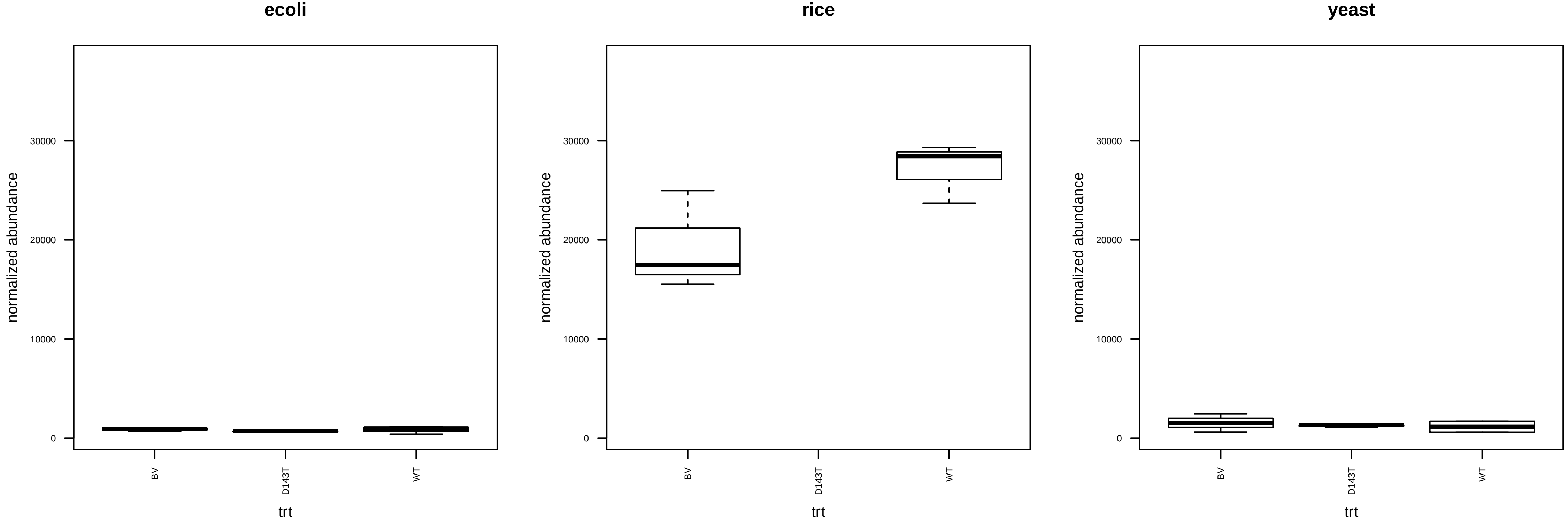

Succinic acid

rt=228
C1549: MSI conf = 1
notes:

	p−value
spp	0.000769
trt	0.041375
spp:trt	0.009995

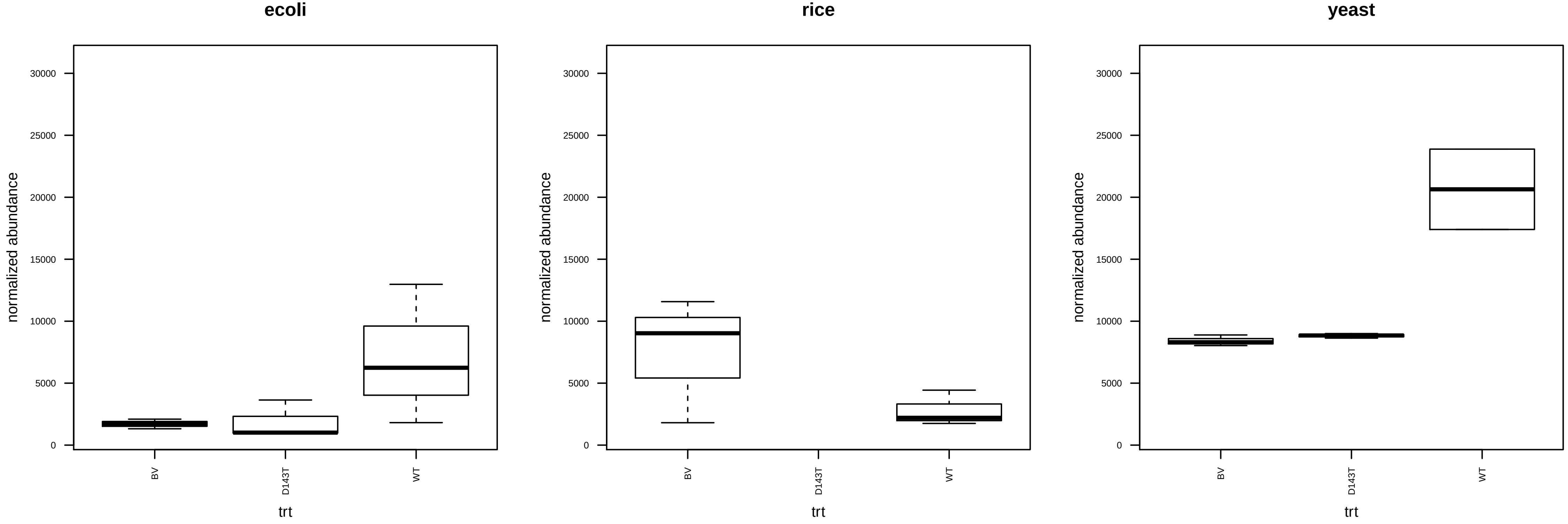

Ribothymidine

rt=126
C1569: MSI conf = 1
notes:

	p−value
spp	< 1e−05
trt	7.86e−05
spp:trt	6.72e−05

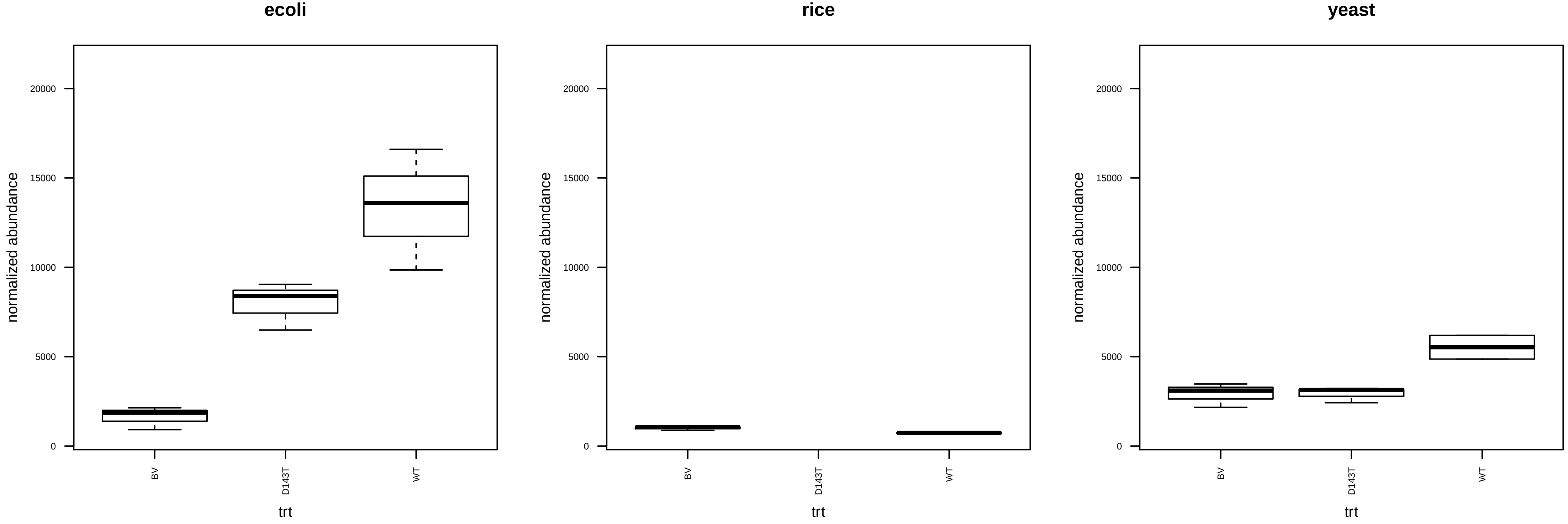

Pyroglutamic acid

rt=106
C1571: MSI conf = 1
notes:

	p-value
spp	<1e-05
trt	0.0687
spp:trt	0.1031

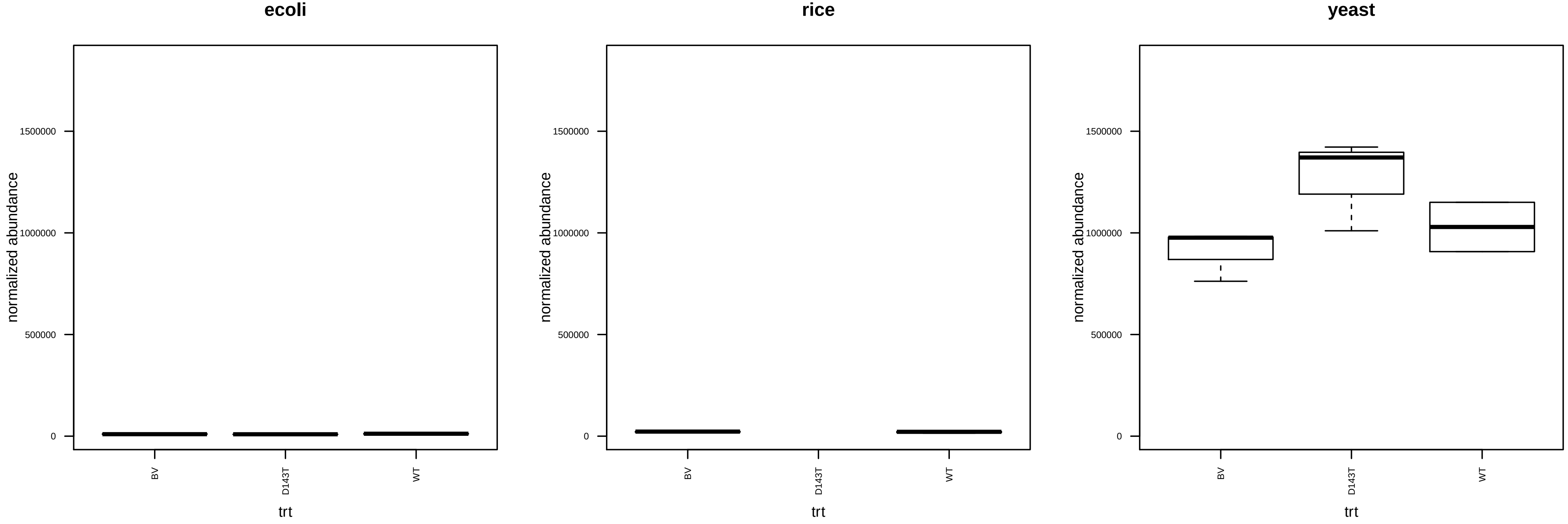

AMP−like

rt=402
C1628: MSI conf = 3
notes:

	p−value
**spp**	<1e−05
**trt**	1
**spp:trt**	1

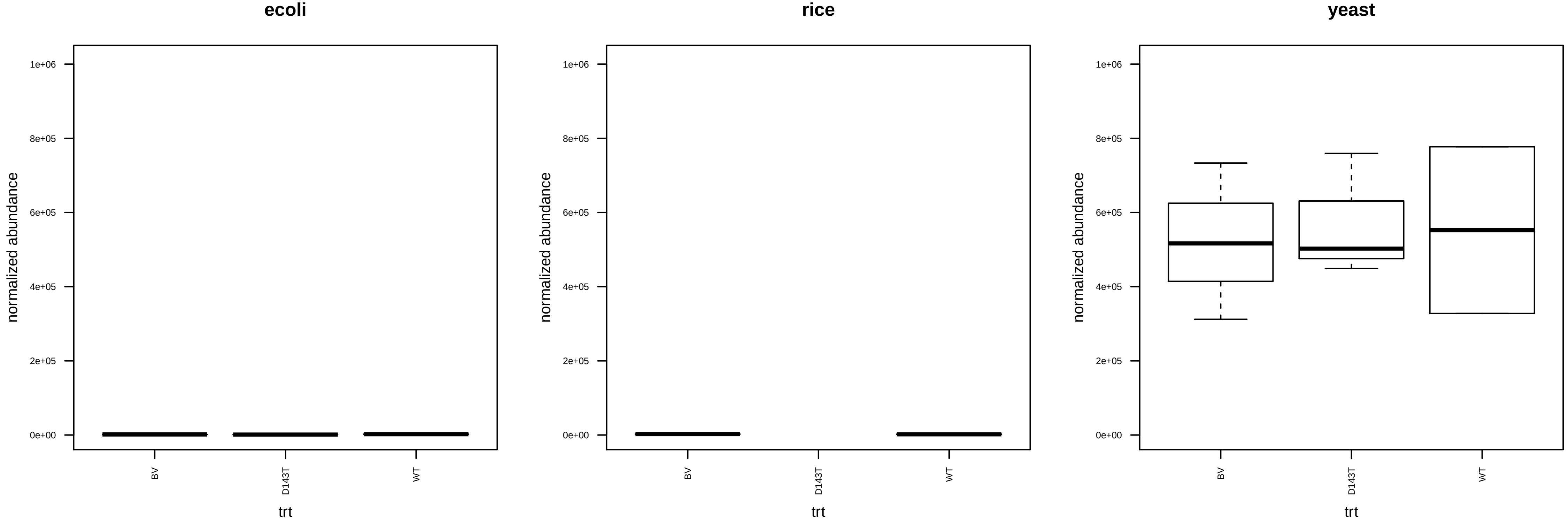

Oxypurinol

rt=272
C1739: MSI conf = 1
notes:

	p−value
spp	< 1e−05
trt	2.77e−05
spp:trt	0.00165

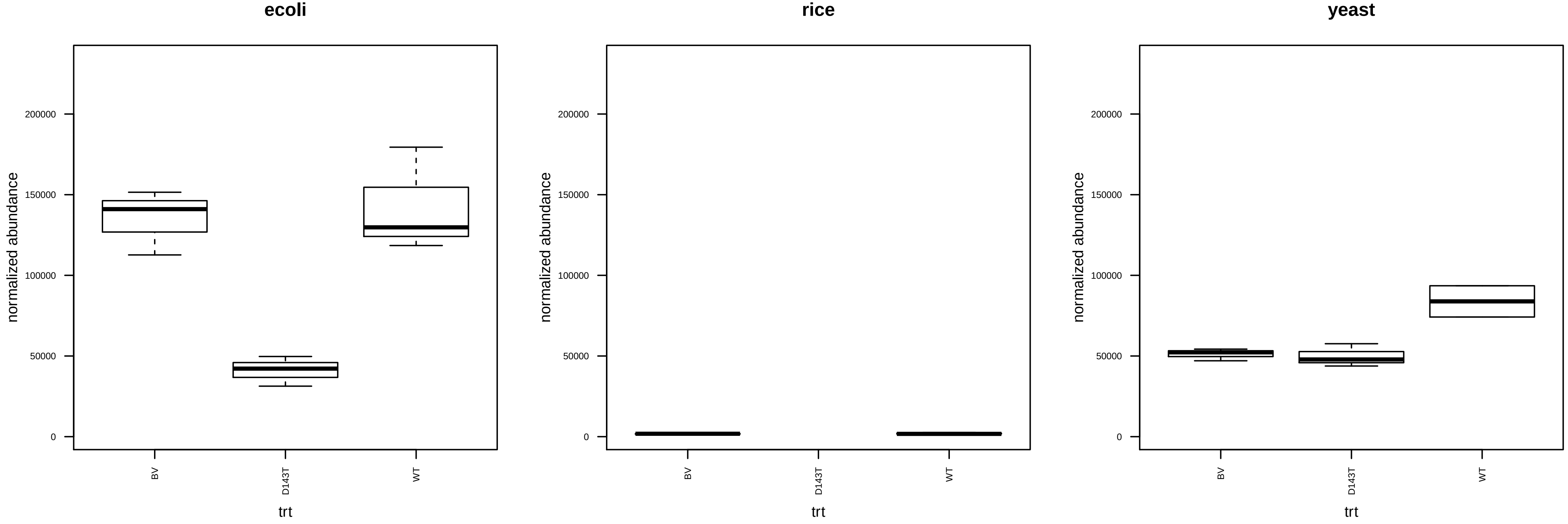

2−Ethyl−2−Hydroxybutyric acid

rt=55
C1854: MSI conf = 1
notes:

	p−value
spp	0.000366
trt	0.147233
spp:trt	0.454154

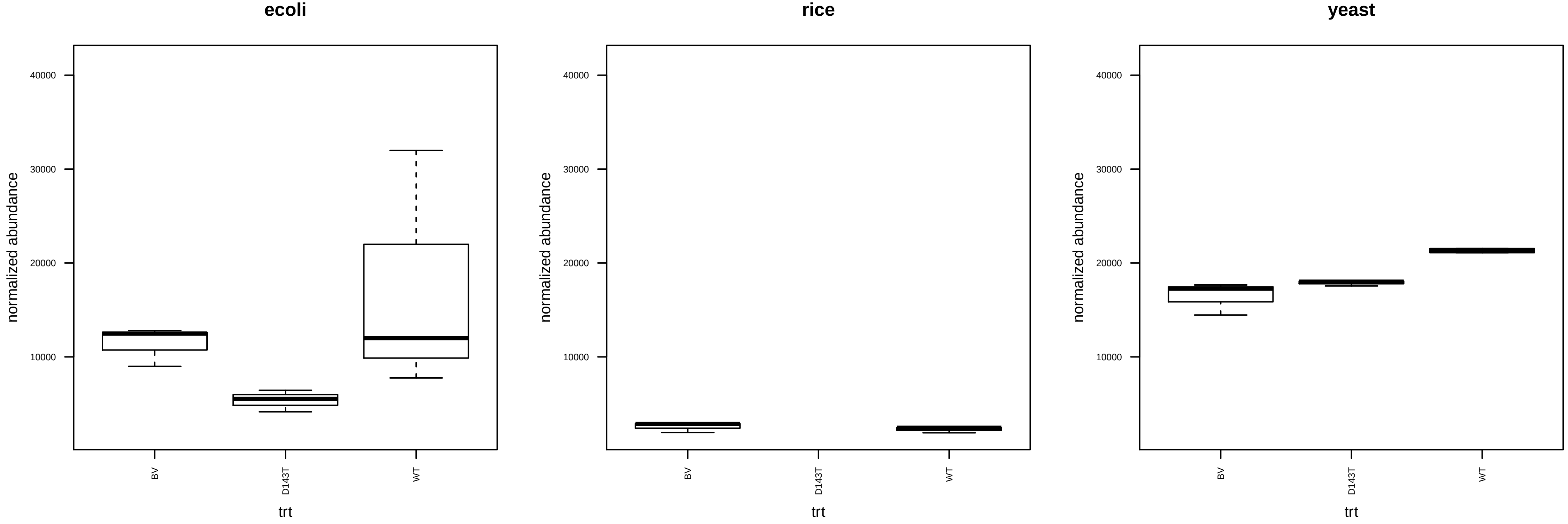

Thymine

rt=135
C1944: MSI conf = 1
notes:

	p−value
spp	8.75e−05
trt	0.0362
spp:trt	0.0186

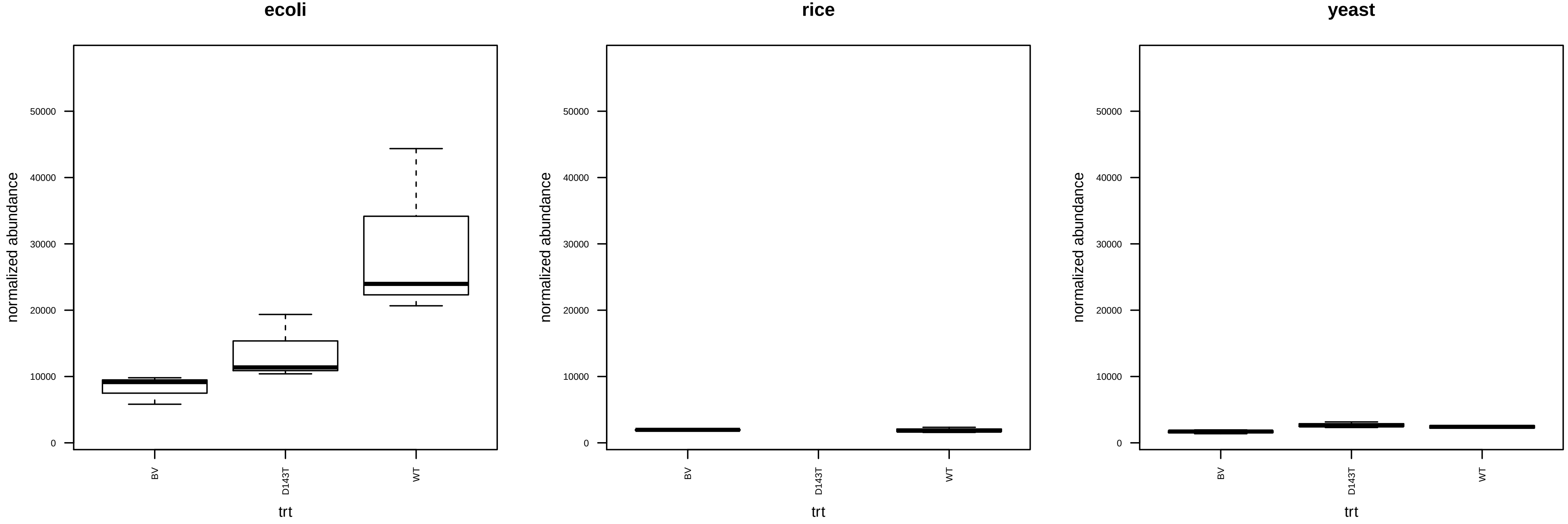

L−Glucose

rt=446
C1994: MSI conf = 1
notes:

	p−value
spp	<1e−05
trt	0.1077
spp:trt	0.0692

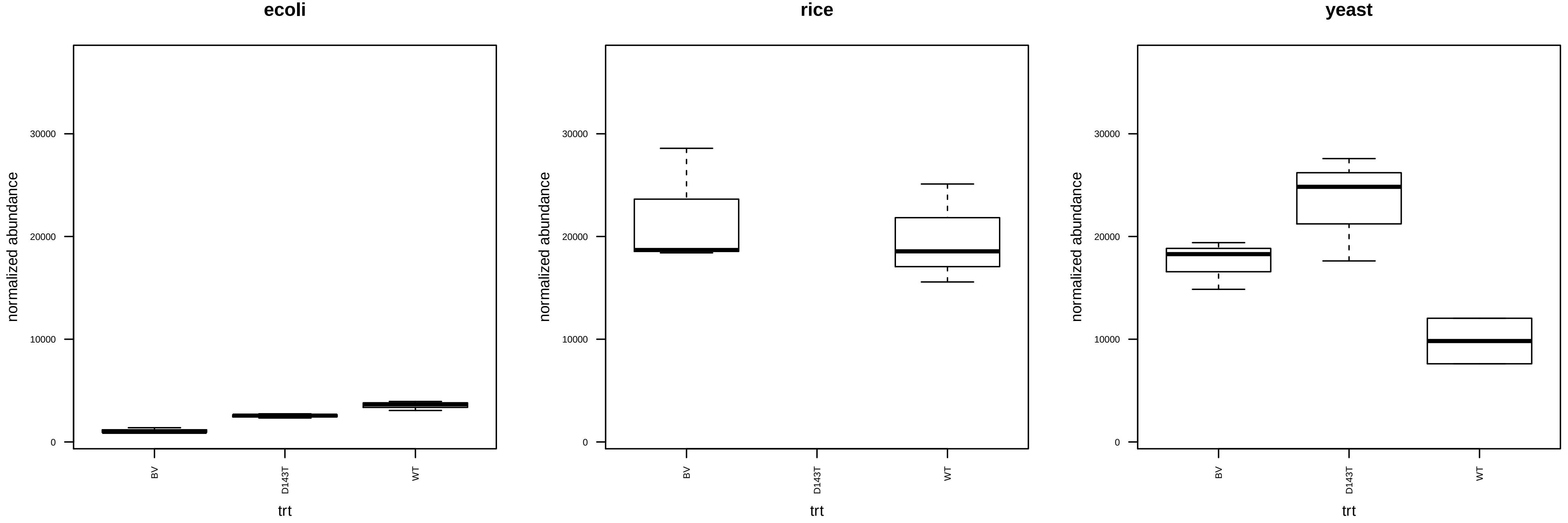

AMP−like

rt=355
C2002: MSI conf = 3
notes:

	p−value
spp	0.00349
trt	0.97652
spp:trt	0.97026

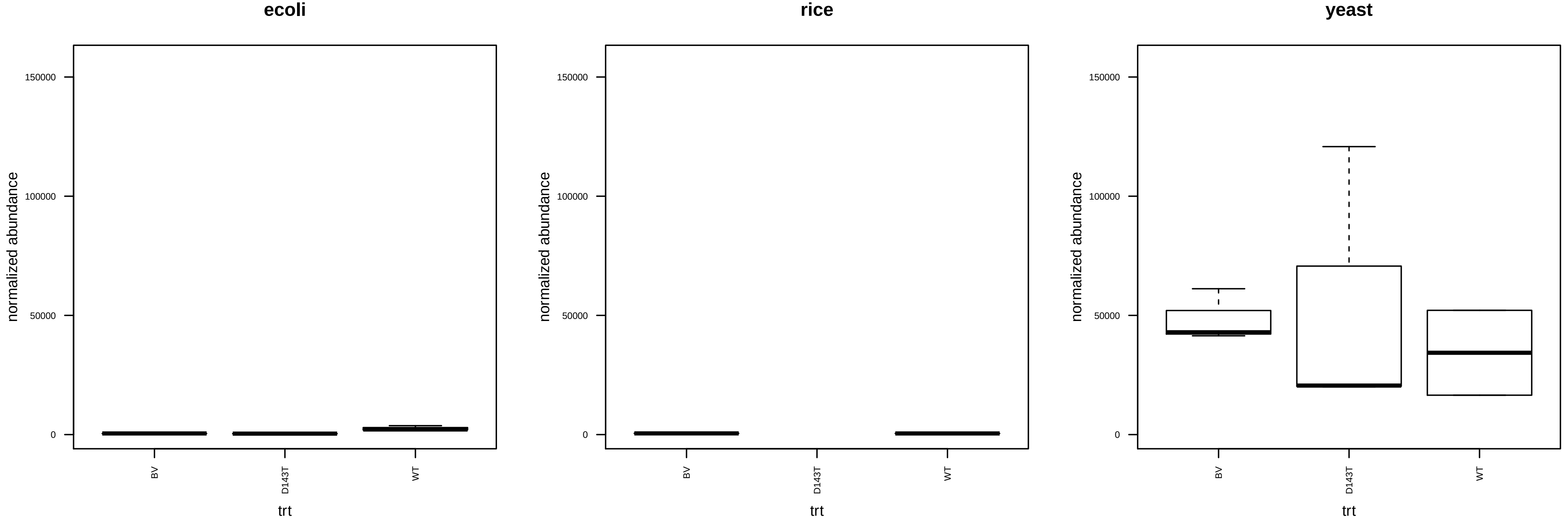

N−Acetylglutamic acid

rt=371
C2003: MSI conf = 1
notes:

	p−value
spp	1.94e−05
trt	0.761
spp:trt	0.889

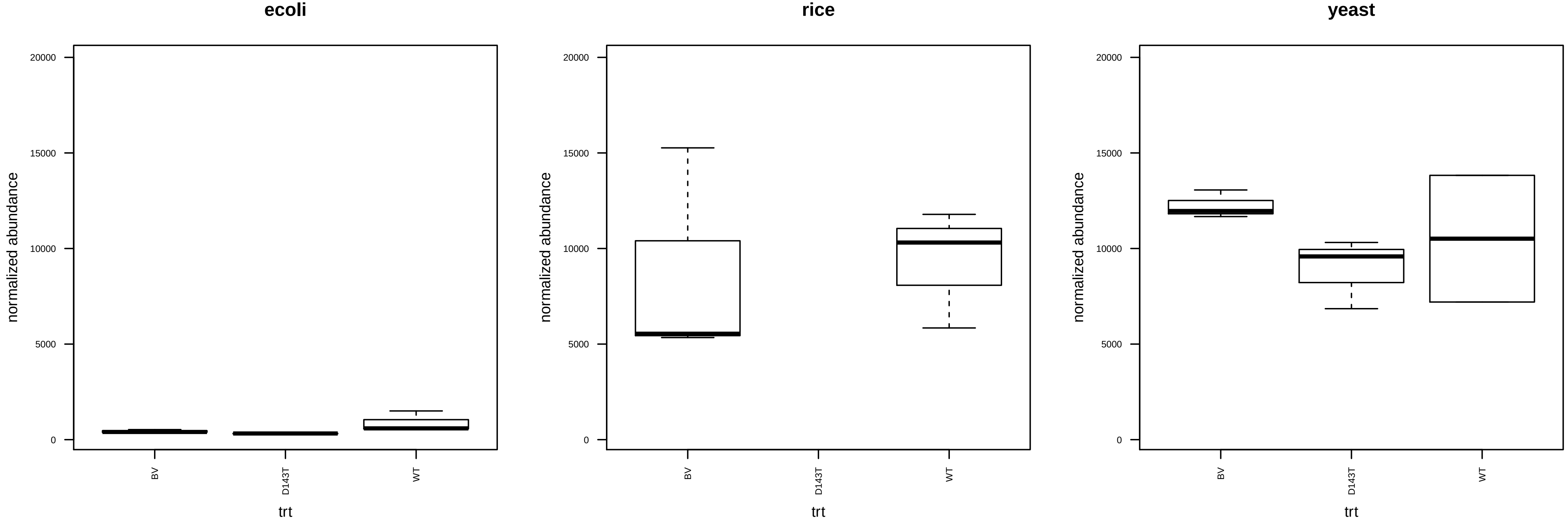

Guanosine 3,5−cyclic−monophosphate

rt=344
C2081: MSI conf = 1
notes:

	p−value
spp	< 1e−05
trt	0.01571
spp:trt	0.00344

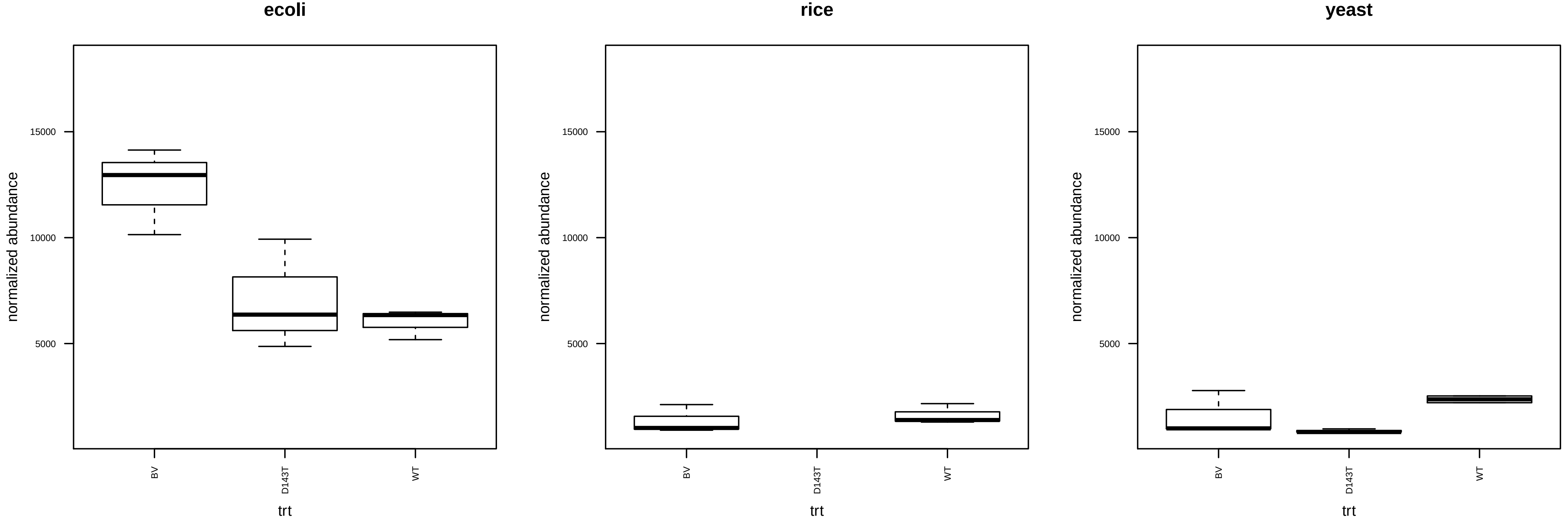

Gluconic acid

rt=335
C2094: MSI conf = 1
notes:

	p−value
spp	0.0285
trt	0.7259
spp:trt	0.3387

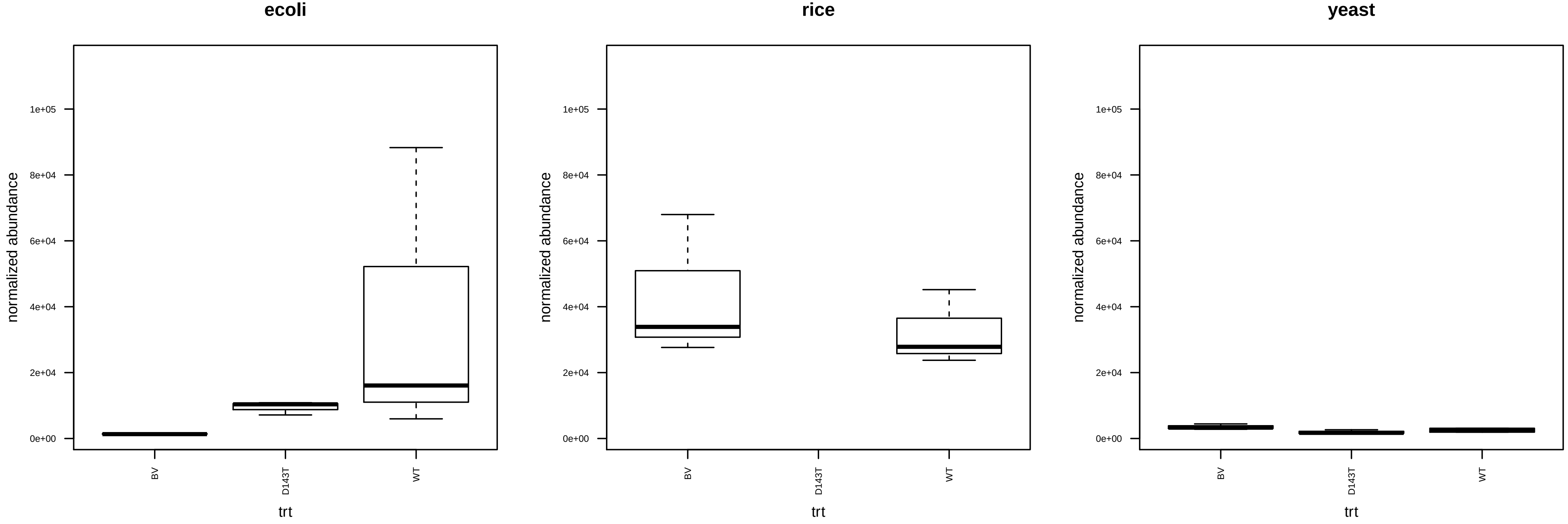

Pantothenic acid

rt=104
C2197: MSI conf = 1
notes:

	p−value
spp	0.007536
trt	0.000618
spp:trt	0.073285

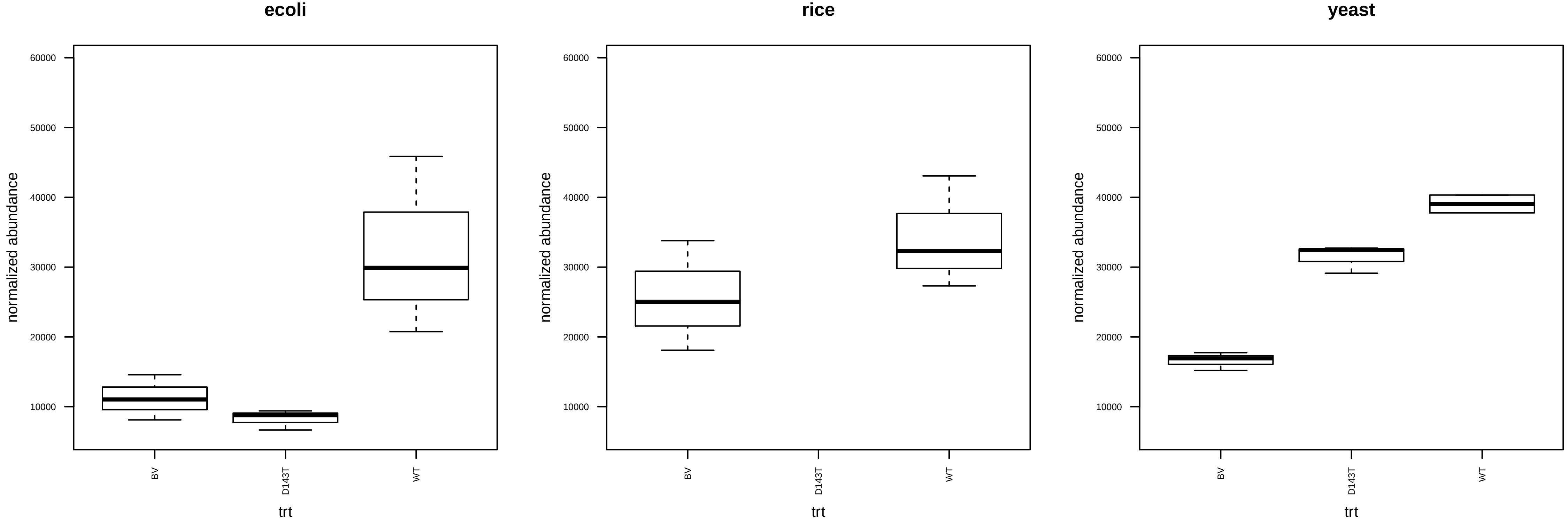

Uracil

rt=195
C2323: MSI conf = 1
notes:

	p−value
spp	0.0188
trt	0.0666
spp:trt	0.0520

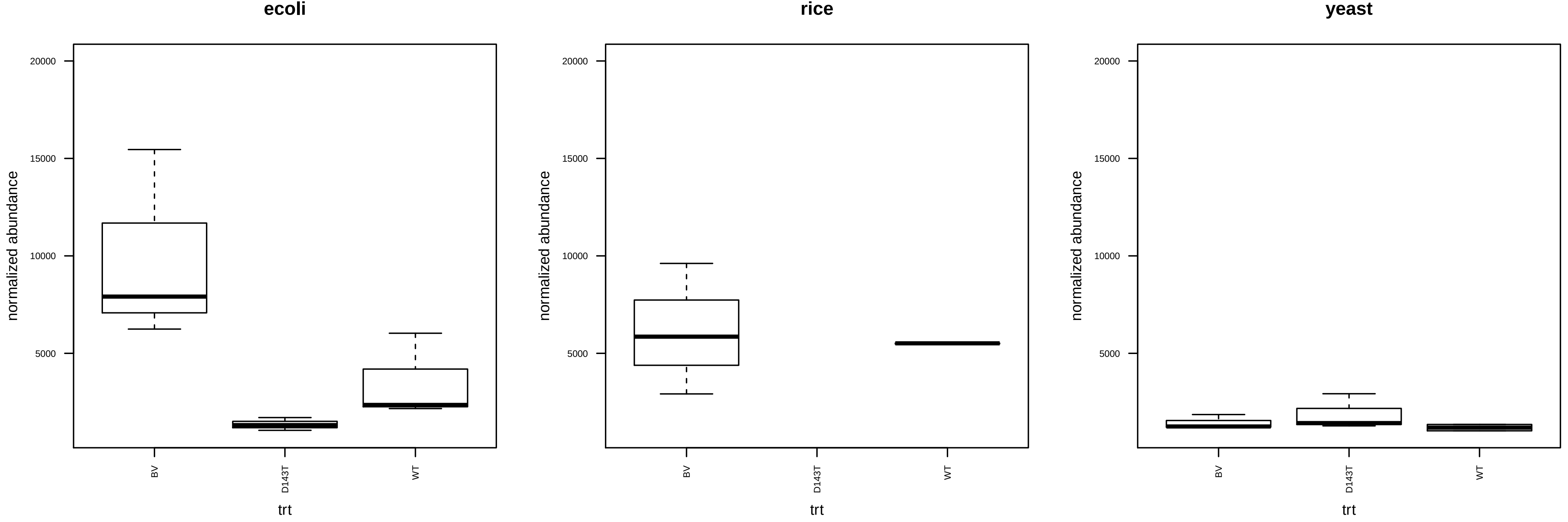

Taurine

rt=402
C2476: MSI conf = 1
notes:

	p−value
spp	0.0195
trt	0.1139
spp:trt	0.7772

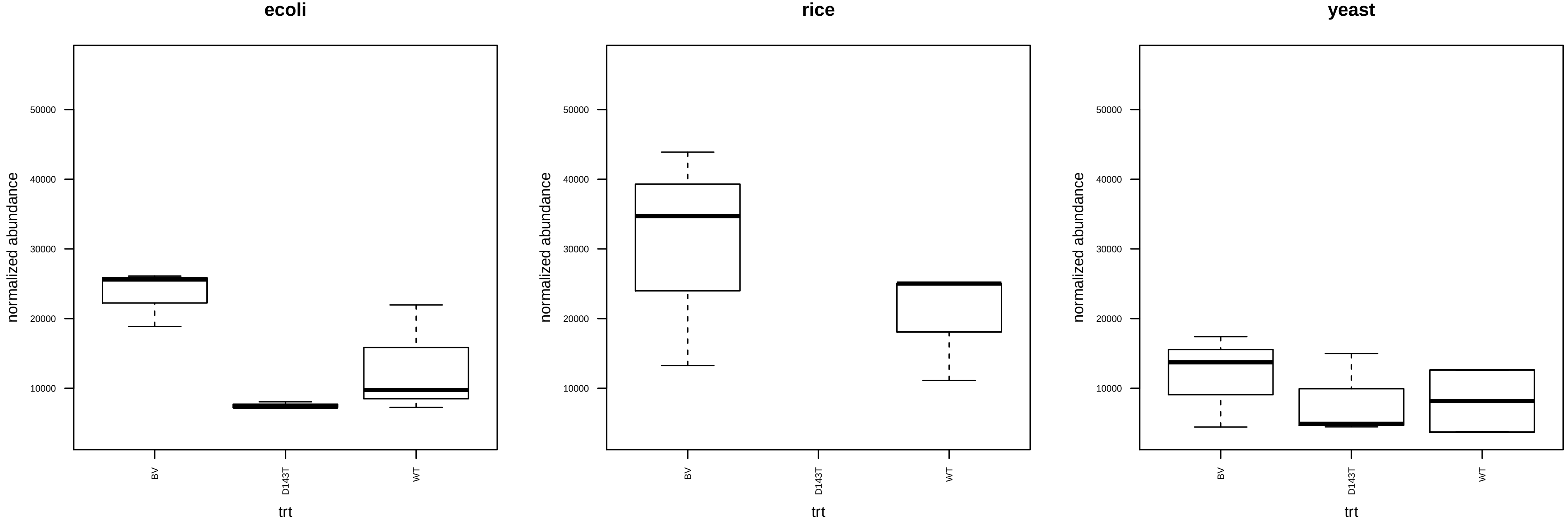

Malic acid

rt=423
C2491: MSI conf = 1
notes:

	p−value
spp	0.00606
trt	0.00999
spp:trt	0.00523

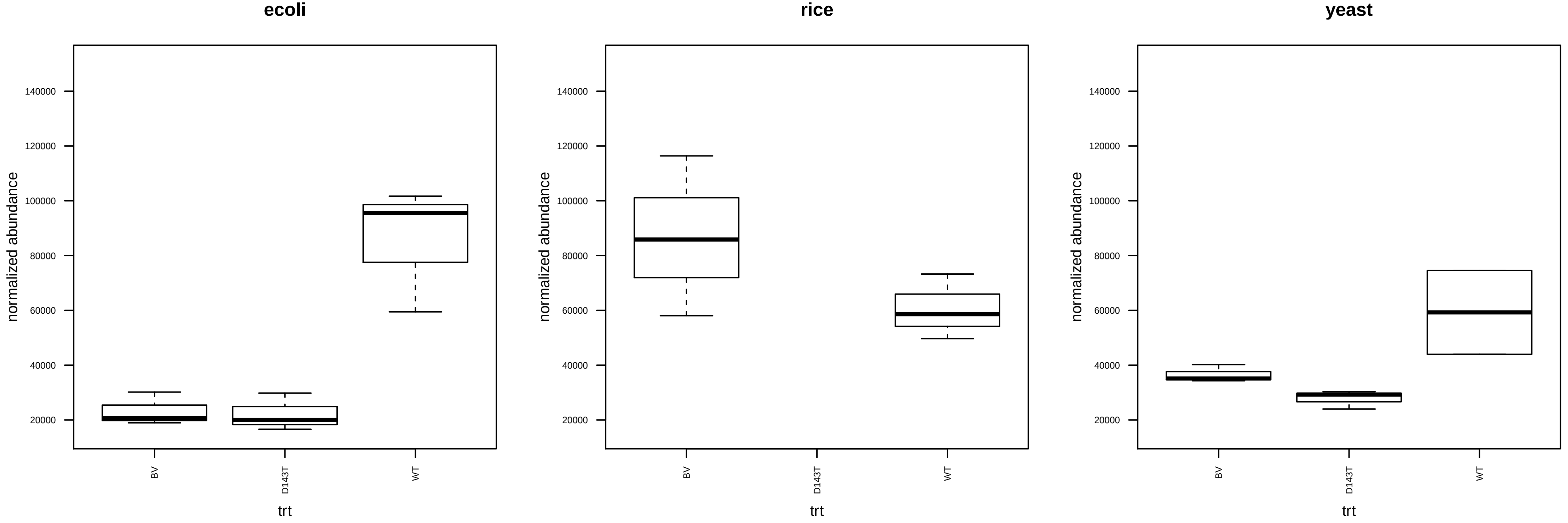

Guanine

rt=367
C2517: MSI conf = 1
notes:

	p−value
spp	1.57e−05
trt	0.059810
spp:trt	0.000927

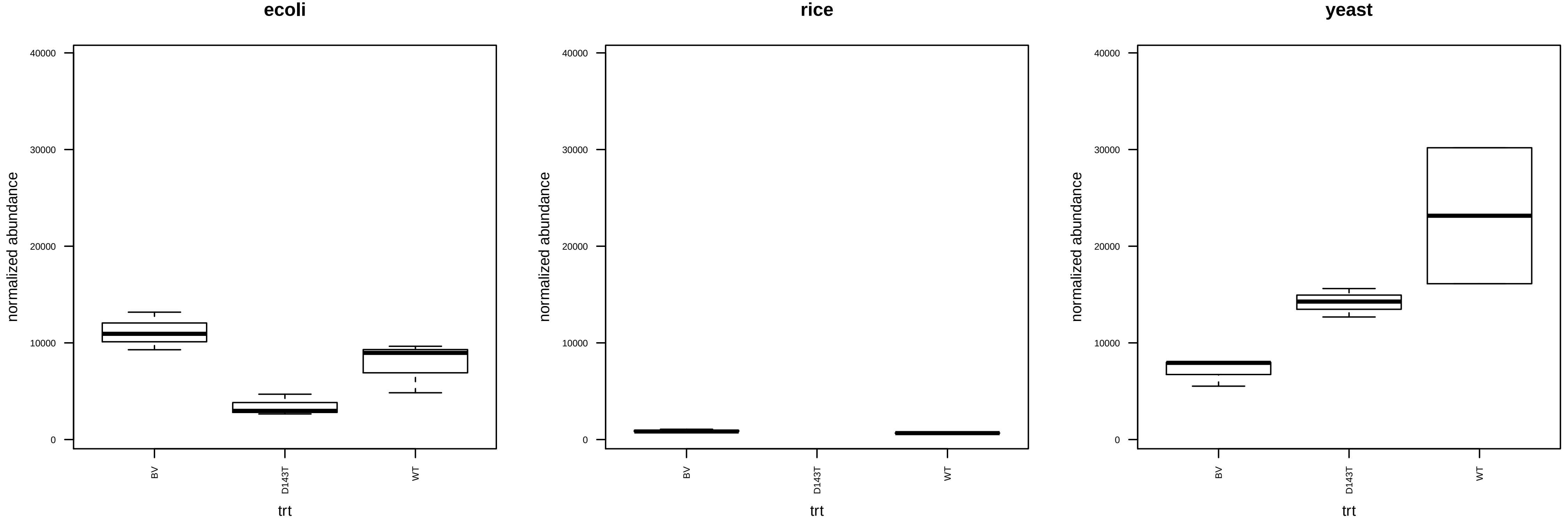

Isocitric acid

rt=601
C2541: MSI conf = 1
notes:

	p−value
spp	2.61e−05
trt	0.979
spp:trt	1.000

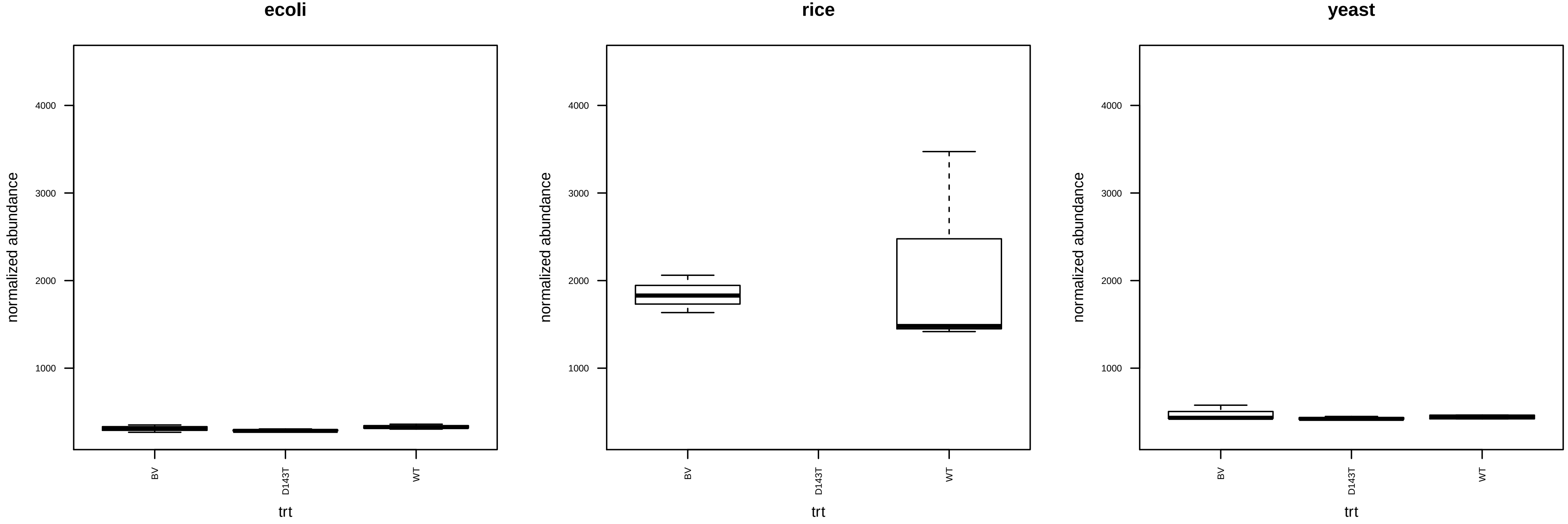

Supplement: S1 File — Boxplots representing the range and standard deviation of the normalized abundance of 79 metabolites upon expression of AvrRxo1, AvrRxo1-D193T, or control in E. coli (ecoli) and S. cerevisiae (yeast), and 48h after infection of rice cv. Kitaake with X. oryzae strain X11-5A expressing avrRxo1 or carrying an empty vector (rice). “BV” denotes pDESTcv in E. coli, pESC-TRP in yeast, and pHM1 in rice. “WT” denotes the AvrRxo1 treatment, and the D193T treatment is here labeled as “D143T” (n = 3 except for the WT treatment in S. cerevisiae, for which n = 2). Anova p-values by species and treatment are shown at the top left, with significant values highlighted in red. (PDF) [file ppat.1006442.s010.pdf]
